# **Hidden diversity in neglected structures**: **A morphological study of the terminalia of *Limnocoris* Stål, 1860 (Hemiptera**: **Heteroptera**: **Naucoridae), with descriptions of two new species from Brazil and taxonomic notes**

**DOI:** 10.1371/journal.pone.0328868

**Published:** 2025-08-13

**Authors:** Rafael P. R. Canejo, Higor D. D. Rodrigues, Felipe F. F. Moreira

**Affiliations:** Laboratório de Entomologia, Instituto Oswaldo Cruz, Fundação Oswaldo Cruz, Rio de Janeiro, RJ, Brazil; Federal University of Espirito Santo: Universidade Federal do Espirito Santo, BRAZIL

## Abstract

*Limnocoris* Stål, 1860 (Hemiptera: Heteroptera: Naucoridae) occurs from the United States to Argentina and is the most speciose genus of Naucoridae, with 75 valid species. Four taxonomic revisions were recently published, which facilitates the identification of described species and the discovery of new ones. Nonetheless, the female terminalia of the genus remains mostly unknown and the male genital capsule has been often ignored due to its uniformity among species. This study aims to fill this gap by providing descriptions and photographs of the female terminalia and male genital capsule for 28 species from Brazil, including two that are described here: *L. curvipenis* **n. sp.** and *L. sitesi* **n. sp.** Both of them display diagnostic characters in the female terminalia, and a distinct male phallosoma is herein recorded for the first time in the genus. The following new synonymies are proposed: *Limnocoris intermedius* Nieser & Lopez-Ruf **n. syn.** and *L. lanemeloi* Nieser & Lopez-Ruf **n. syn.** as junior synonyms of *L. decarloi* Nieser & Lopez-Ruf, and *L. nigropunctatus* Montandon **n. syn.** as junior synonym of *L. pauper* Montandon. The following species are resurrected from synonymy and their species status are restored: *Limnocoris admontandoni* La Rivers **stat. restit.** from synonymy with *L. insignis* Stål, and *L. sattleri* De Carlo **stat. restit.** from synonymy with *L. nigropunctatus*. Our work reveals a notable diversity in the female terminalia of the taxa examined and provides a basis for future taxonomic and systematic studies on this and other genera of Naucoridae.

## Introduction

*Limnocoris* Stål, 1860 is the most speciose genus of Naucoridae (Hemiptera: Heteroptera), with 75 valid species [1]. The genus has undergone extensive revisions in the past decades and its taxonomy is based mainly on the external morphology, especially on the shape of the mesosternal and metasternal carinae, the body pilosity, and the shape of the median lobes of male abdominal tergum VIII [1–4].

Unlike in other taxa of Naucoridae, such as Ambrysinae, the male genital capsule of *Limnocoris* has been shown to be uninformative in species identification and has been rarely described or illustrated, except in De Carlo [5], Lee [6], Nieser & Lopez-Ruf [2] and Sites [7]. In terms of female diagnostic characters, only the shape of the subgenital plate (= abdominal mediosternite VII) is usually employed. Other terminal abdominal structures remain poorly explored within the family and may offer useful information. In studies where such structures were considered in the family, they have been taxonomically useful, as in the case of female abdominal tergum VIII, and the first and second valvulae in *Pelocoris* Stål, 1876 [8–12].

In order to investigate structures that have not been studied yet, males and females of 28 species of *Limnocoris* collected in Brazil were examined, and the structures of the female terminalia and male genital capsule were illustrated, described and compared among congeners in a comments section for each species. 26 of these species represent almost all of the recorded species for the country. The only species recorded for Brazil that were not examined, considering the nomenclatural acts proposed in this study and those that will be published in a forthcoming revision of the *Limnocoris* from southeastern South America, are *L. abbreviatus* La Rivers, 1964, *L. aculabrum* La Rivers, 1974, *L. montandoni* La Rivers, 1974, and *L. saphis* Nieser & Lopez-Ruf, 2001. This effort revealed three new synonymies, the resurrection of two species, and the existence of two new species of the genus, which are described, illustrated and compared with morphologically similar congeners.

## Materials and methods

The material examined here is deposited in the following collections: California Academy of Sciences, San Francisco, United States (CAS); Entomological Collection of the Instituto Oswaldo Cruz, Rio de Janeiro, Brazil (CEIOC); Entomological Collection Adalberto Antônio Varela-Freire, Universidade Federal do Rio Grande do Norte, Natal, Brazil (CEAAVF); Departamento de Parasitologia, Universidade Federal de Minas Gerais, Belo Horizonte, Brazil (DPIC); Entomological Collection José Alfredo Pinheiro Dutra, Universidade Federal do Rio de Janeiro, Rio de Janeiro, Brazil (DZRJ); Invertebrates Collection, Instituto Nacional de Pesquisa da Amazônia, Manaus, Brazil (INPA); Laboratório de Entomologia Aquática, Universidade Estadual do Maranhão, Caxias, Brazil (LEAq); Laboratório de Ecologia e Taxonomia de Invertebrados, Universidade Federal do Oeste do Pará, Santarém, Brazil (LETIA); Museu de Zoologia, Universidade de São Paulo, São Paulo, Brazil (MZUSP); Nieser Collection, Tiel, The Netherlands [private collection] (NCTN); United States National Museum of Natural History, Washington D.C., United States (USNM); and Zoologische Staatssammlung München, Munich, Germany (ZSMC).

Part of the material was obtained during fieldwork conducted in 2023 and 2024, in the Brazilian states of Espírito Santo, Paraná, Rio Grande do Norte, Rio Grande do Sul, São Paulo, and Santa Catarina. Specimens were collected by scraping the bottom of shallow streams with sand and/or gravel substrate using an aquatic entomological D-net. The insects were either pinned or preserved in 96% ethanol and labeled.

Images were obtained using a Leica M205 C stereomicroscope coupled with a Leica DMC 2900 digital camera and captured using the Leica Application Suite version 4.7.1, followed by image preparation with Adobe Photoshop CS6. A distribution map of the new species herein described was produced using QGIS 3.26.1. All measurements are given in millimeters. Abdominal segment numbers are expressed as Roman numerals. The terms brachypterous and macropterous present throughout the text refer to the condition of the hind wing: brachypterous specimens have the hind wing reduced (with various degrees of reduction), whereas macropterous specimens have the hind wing fully developed. We follow the terminology by Snodgrass [13] who refers to the female genital structures as valvifers and valvulae. Part of the male terminalia (abdominal terga VI–VIII) of most of the species of *Limnocoris* studied here were already illustrated and described in recent revision studies [1–4]; therefore, such structures are not treated here.

### Nomenclatural acts

The electronic edition of this article conforms to the requirements of the amended International Code of Zoological Nomenclature, and hence the new names contained herein are available under that Code from the electronic edition of this article. This published work and the nomenclatural acts it contains have been registered in ZooBank, the online registration system for the ICZN. The ZooBank LSIDs (Life Science Identifiers) can be resolved and the associated information viewed through any standard web browser by appending the LSID to the prefix “http://zoobank.org/”. The LSID for this publication is: urn:lsid:zoobank.org:pub: 34DF8859-E893-43C9-8D82-ECADC521F1F5. The electronic edition of this work was published in a journal with an ISSN, and has been archived and is available from the following digital repositories: PubMed Central and LOCKSS.

## Results

### Female terminalia

**Fig 1:**
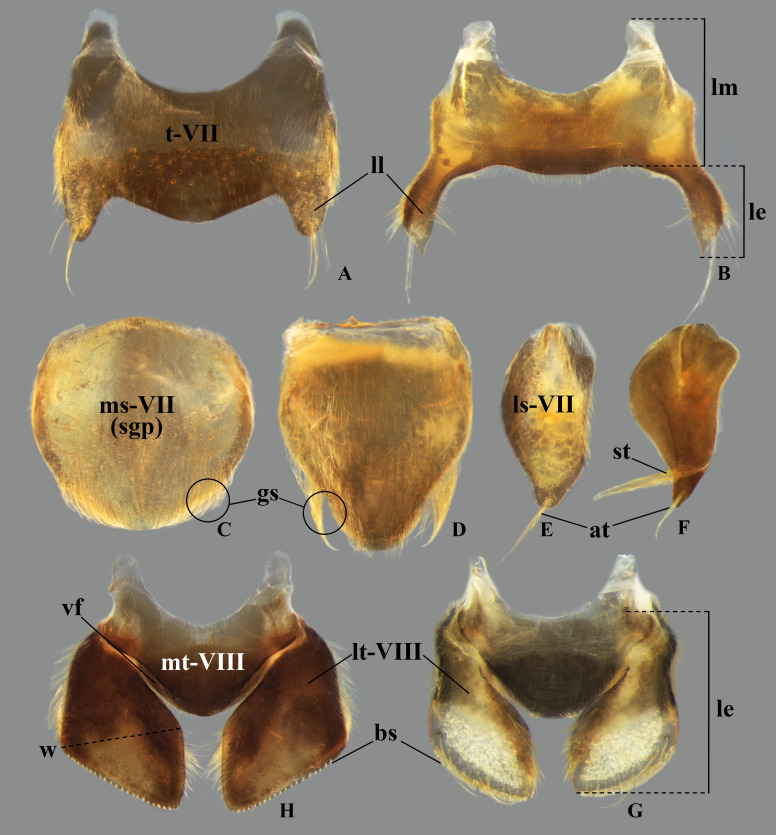
Structures of the female terminalia of *Limnocoris.* **(A–B)** Abdominal tergum VII, **(A)** *L. acutalis*, **(B)** *L. machrisi*. **(C–D)** Mediosternite VII, **(C)** *L. rotundatus*, **(D)** *L. machrisi*. **(E–F)** Laterosternite VII, **(E)** *L. acutalis*, **(F)** *L. burmeisteri*. **(H–G)** Abdominal tergum VIII, **(H)** *L. volxemi*, **(G)** *L. pusillus*. at = apical tuft of long setae, bs = brush-like setae, gs = golden setae, le = length of the structure, ll = lateral lobes, lm = basal half of lateral margin of tergum VIII, ls-VII = laterosternite VII, lt-VIII = laterosternite VIII, ms-VII = mediosternite VII, mt = VIII = mediotergite VIII, sgp = subgenital plate, st = subapical tuft of setae, w = width of the structure, vf = ventral fold.

**Fig 2:**
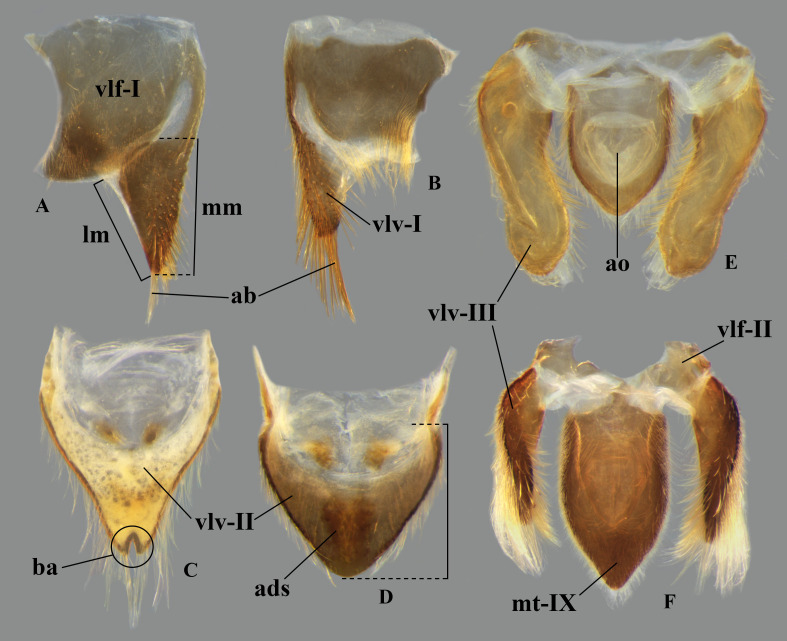
Structures of the female terminalia of *Limnocoris.* **(A–B)** Valvifer I + Valvula I of **(A)** *L. volxemi* and **(B)** *L. curvipenis* **n. sp. (C–D)** Valvulae II of **(C)** *Limnocoris* sp. C and **(D)** *L. siolii*. **(E)** Valvifer II + Valvulae III + Mediotergite IX of **(E)** *L. insignis* and **(F)** *L. machrisi*. ads = apical dark spot, ao = anal operculum, ba = bifurcated apex, lm = lateral margin, mm = median margin, mt-IX = mediotergite IX, vlf-I = valvifer I, vlf-II = valvifer II, vlv-I = valvula I, vlv-II = valvulae II, vlv-III = valvulae III.

In *Limnocoris*, as in other genera of Naucoridae, the female terminalia consist of abdominal segments VII to IX. *Abdominal tergum VII*: rectangular, with distinct lateral lobes at posterolateral margins, bearing a tuft of long setae near apex (Figs 1A–B); shape of lateral lobes and posterior margin usually characteristic of each species, with little intraspecific variation for most (represented in dorsal view in all figures). *Abdominal sternum VII:* divided into a mediosternite (subgenital plate) and a pair of laterosternites; subgenital plate with or without elongate golden setae posterolaterally (Figs 1C–D); laterosternites with apical tuft of long setae, some species also with a second tuft subapically, with setae extending mesad (Figs 1E–F) (represented in ventral view in all figures). *Abdominal tergum VIII*: divided into a mediotergite and a pair of laterotergites; posterolateral margins of mediotergite ventrally folded in some species due to how laterotergites rest over it (Fig 1H); laterotergites serrated at posterior margin, with minute to large brush-like setae between each tooth of the serration (Figs 1H–G) (represented in dorsal view in all figures). *Abdominal sternum VIII*: comprising the ventral portion of the ovipositor with valvifer I + valvula I (= anterior gonapophysis); valvifer I with almost no interspecific variation, except for slight shape differences among mesal margins; valvula I triangular, with basal half concealed by valvifer I, bearing several stout, posteriorly directed setae, these becoming larger towards the apex (Figs 2A–B) (represented in ventral view in all figures). *Abdominal tergum IX*:; mediotergite IX conical or ogival, bearing anal operculum ventrally (Figs 2E–F) (represented in ventral view in all figures). *Abdominal sternum IX*: comprising the dorsal portion of the ovipositor with valvulae II ventrally and valvifer II + valvulae III dorsally (= posterior gonapophysis) valvifer II located at base of valvula III and mediotergite IX (although muscle fibers are concentrated with valvula III), reduced to a small, lightly sclerotized structure, which probably serves only for muscle attachment (Figs 2E–F); valvifer II showed a lot of intraspecific variation and is not described below for each species; valvula III located lateroventrally to valvifer II, its shape and pubescence varying significantly among species (Figs 2E–F); valvulae II usually completely fused medially, partially fused in some specimens, resulting in a bifurcated apex and sometimes a visible suture at midline (Figs 2C–D); posterior region sometimes pigmented only medially near apex, resulting in an apical dark spot (Fig 2D) (represented in ventral view in all figures, unless stated otherwise).

## Male terminalia

**Fig 3:**
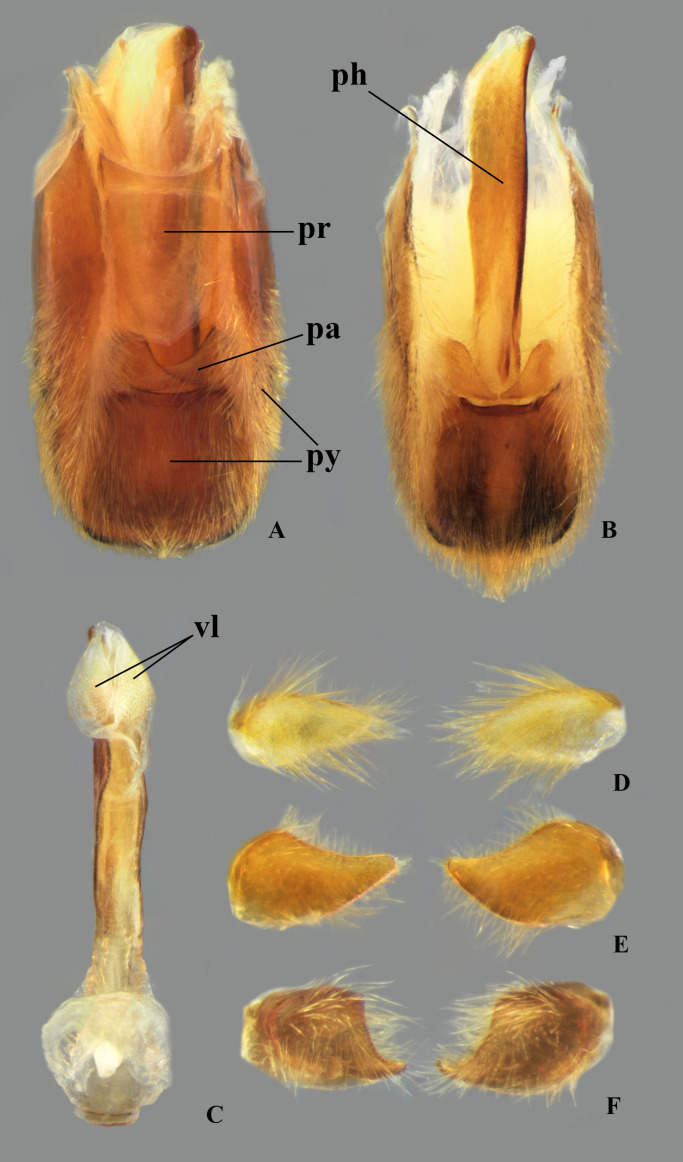
Structures of the male genital capsule of *Limnocoris.* Genital capsule of **(A)** *L. espinolai*, **(B)** *L. melloleitaoi* (proctiger removed), **(C)** phallosoma of *L. melloleitaoi* in ventral view, **(D–F)** dorsal view of parameres of **(D)** *L. decarloi*, **(E)** *L. volxemi* and **(F)** *L. amazonensis*. pa = paramere, ph = phallosoma, pr = proctiger, py = pygophore, vl = ventral lobes of the phallosoma.

The male terminalia consist of abdominal terga VI to X. Male accessory genitalic process of tergum VI present or absent; when present, generally poorly developed. Posterior margin of tergum VII exhibiting variation among species, although sometimes very similar among closely related taxa. Unlike in other naucorid genera, median lobes of tergum VIII asymmetrical and sometimes displaying interspecific variation (see Rodrigues & Sites, [1,3,4]). Male genitalia relatively uniform among species, except one (Fig 35E). Pygophore with sparse elongate setae throughout most of surface, except a dense tuft of setae posteromedially in most species (Figs 3A–B). Phallosoma elongated, slightly rotated dextrally in dorsal view, laterally flattened, surpassing level of lateral margins of genital capsule (Fig 3B). In some species, apex of phallosoma slightly angled dextrally in dorsal view (Fig 3B). Ventral lobes membranous (Fig 3C). Parameres symmetrical (some seem slightly assymetrical in the figures due to positioning), sometimes overlapping at midline, curved ventrally, with sparse, medially directed setae throughout ventral surface (Figs 3D–F).

**Fig 4:**
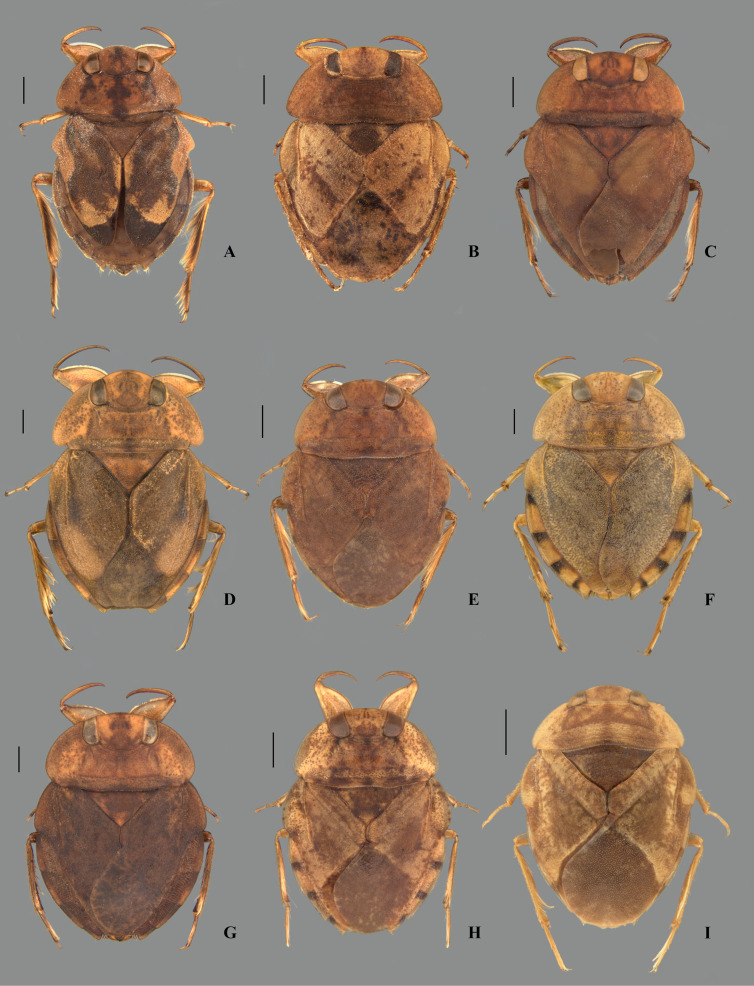
Dorsal habitus of *Limnocoris* spp. **(A)** *Limnocoris acutalis*, **(B)** *L. amazonensis*, holotype, **(C)** *L. asper*, **(D)** *L. brasiliensis*, **(E)** *L. burmeisteri*, **(F)** *L. decarloi*, **(G)** *L. espinolai*, **(H)** *L. fittkaui*, **(I)** *L. illiesi*. Scale bar = 1 mm.

**Fig 5:**
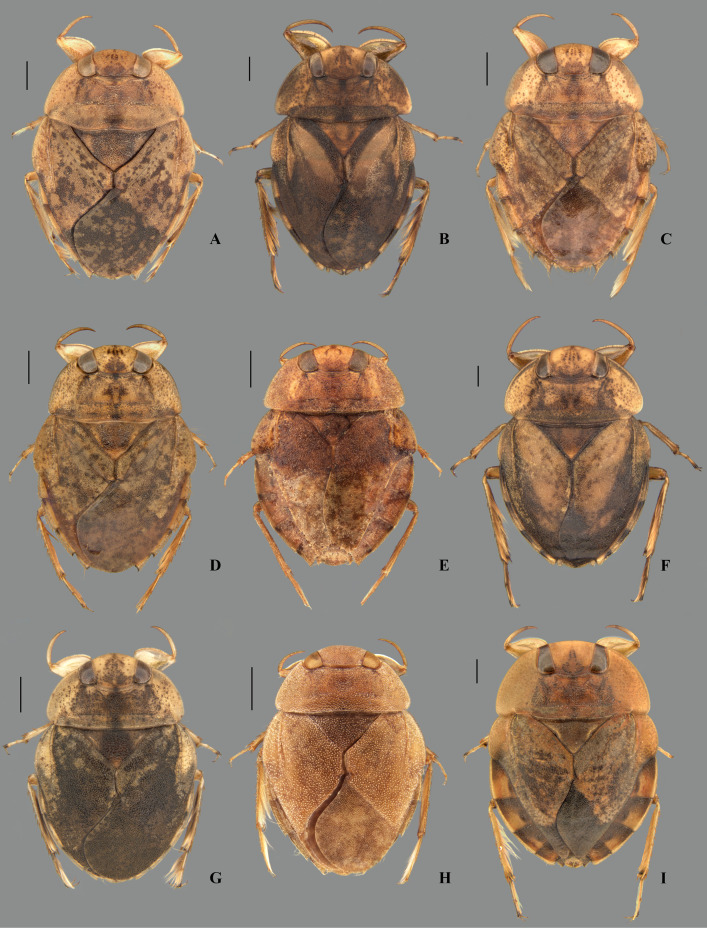
Dorsal habitus of *Limnocoris* spp. **(A)** *Limnocoris machrisi*, **(B)** *L. melloleitaoi*, **(C)** *L. menkei*, **(D)** *L. minutus*, **(E)** *L. moreirai*, paratype, **(F)** *L. pauper*, **(G)** *L. pusillus*, **(H)** *L. reynosoi*, **(I)** *L. rotundatus*. Scale bar = 1 mm.

### Supplemental and original descriptions

***Limnocoris acutalis* La Rivers, 1974**

(Figs 4A, 7A, 9A, 11A, 13A, 15A, 17A, 19A–D, 23A)

*Limnocoris acutalis* La Rivers, 1974: 6–7 (original description).

#### Material examined.

All specimens brachypterous. **BRAZIL, Minas Gerais**: Baependi, Cachoeira de Itaúna, ca. 15 km S of Baependi, 22°02’53.7“S, 44°49’21.1”W, 923 m, 02.xii.2016, H.D.D. Rodrigues col. (2♂, 1♀ brachypterous, CEIOC); Serra do Cipó, [Santana do Riacho], MG-010, Km 101, Rio Cipó, em plantas, [–19.32, –43.61], 27.vii.1972, C.G. Froehlich col. (1♀ brachypterous, CEIOC). **Rio de Janeiro**: 4628, Nova Friburgo, Rio Bonito de Lumiar, Ramalhete, Rio Bonito, 22°24’38.5”S, 42°20’40.7”W, 24.x.2010, J.L. Nessimian col. (1♂ brachypterous, DZRJ); Brasil, RJ, Nova Friburgo, Cascata, Rio Macaé, Ponto RB14, 22°22’03.2”S, 42°15’29.8”W; 08.iii.2009; G.A. Jardim col. (1♂ brachypterous, DZRJ).

#### Female terminalia.

Tergum VII with average width 2.5x length at midline; lateral margin straight or slightly convex, bearing long setae on posterior half; posterior margin strongly convex, reaching halfway or further the length of lateral lobe; lateral lobe as long as wide (Fig 7A). Subgenital plate with uniform pilosity, lateral margins subparallel in basal 1/3, converging to rounded apex in apical 2/3. Laterosternite VII convex on mesal margin, bearing a single tuft of long setae posteriorly; lateral margin slightly sinuous; apex narrowly rounded (Fig 9A). Mediotergite VIII with lateral margin almost straight; posterior margin rounded. Laterotergite VIII length about three times greatest width; apical half of lateral margin weakly serrated, bearing large brush-like setae (Fig 11A). Valvifer I with mesal margin convex. Valvula I bearing robust, posteriorly directed setae, setae becoming longer towards apex; apical robust setae shorter than lateral margin (Fig 13A). Valvulae II slightly wider than long, bearing uniform pilosity on dorsal surface; lateral margin slightly convex; apex broadly rounded, bearing apical dark spot (Fig 15A). Valvula III flattened, with sparse setae basally, becoming denser distally. Mediotergite IX triangular, longer than valvula III, length 1.5x times greatest width; width at base about twice the width of valvula III; lateral margins angulated at mid-length, converging to narrowly rounded apex (Fig 17A).

#### Male genitalia.

Pygophore with elongate setae sparsely distributed over most of surface, with anterior and posterior margins nearly straight. Phallosoma straight (Figs 19A–D). Paramere with anterior margin straight towards mesal angle, mesal angle narrowly rounded (Fig 23A).

#### Comments.

The female terminalia of this species have some structures very similar to those of *L. siolii* (De Carlo, 1966). They have nearly identical subgenital plates, laterosternites VII, valvulae II–III and mediotergite IX. They also share the unique shape of laterotergite VIII, which is narrow and has very large brush-like setae on the posterior half of the lateral margin (Figs 11A, 12E). The female terminalia of both species can be differentiated as follows: tergum VII of *L. acutalis* has the posterior margin convex and lateral lobes rounded (Fig 7A), whereas *L. siolii* has the posterior margin straight and the lateral lobes triangular (Fig 8E); and in *L. acutalis* the posterior part (beyond the posterior margin of valvifer I) of valvula I is longer than wide (Fig 13A), whereas in *L. siolii* it is as wide as long or wider than long (Fig 14E). Regarding the male genitalia, the only difference observed is in the phallosoma, which is narrow and long in *L. acutalis*, with the ventral lobes surpassing the level of the dorsolateral projections of the genital capsule (Figs 19A–D), whereas in *L. siolii* the phallosoma is broad and short, with the apex barely surpassing the level of the dorsolateral projections of the genital capsule (Figs 22I–L).

***Limnocoris admontandoni* La Rivers 1974**

(Figs 25–27)

*Limnocoris admontandoni* La Rivers, 1974 (original description) **stat. restit.**

*Limnocoris admontandoni*: Nieser & Lopez-Ruf 2001: 279 (synonymized with *L. insignis*)

#### Type material examined.

HOLOTYPE, ♀ macropterous: **BRAZIL, São Paulo**: R. Guarau, 1963 Dec., Jacupiranga, 24°45[‘S], 48°02[‘W], F. Plaumann/ *Limnocoris admontandoni* Holotype ♀/ California Academy of Sciences Type No. 13417/ Ira la Rivers Collection, Bequeathed to the California Academy of Sciences - 1978 (CAS); PARATYPES: same data as holotype (1♂ brachypterous, 2♂ macropterous, 2♀ macropterous CAS).

#### Additional material examined.

All specimens brachypterous: **BRAZIL, São Paulo**: Sete Barras, SP14, Rio Preto, 24°11’35.0“S, 47°53’26.0”W, 30 m, 19.xi.2023, L.L. Dumas, J.M.S. Rodrigues & R.P.R. Canejo col. (CEOIC). (6♂, 6♀ CEIOC); Eldorado, CDD09, Rio Batatal, Trilha para a cachoeira do machadinho, 24°39’45.5”S, 48°19’41.7”W, 100 m, 04.vii.2024, E.A. Joaquim, L.L. Dumas, K.O. Souza & R.P.R. Canejo col. (3♂, 1♀ CEIOC).

#### Diagnosis.

Body length 8.00–8.70, body width 6.00–6.70. This species can be distinguished from congeners by the anterior margin of the head in front of the eyes distinctly extended anteriorly, about 24% the head length (Fig 25B); article III of the labium more than twice the length of article IV (Fig 25D); and the female valvula III with a deep transverse indentation on the posterior region of the ventral surface (Fig 25E).

#### Supplemental description.

Overall dorsal coloration yellowish-brown, mottled brown on head, pronotum and hemelytra. Dorsal surface with fine granulation, punctate throughout. Ventral coloration mostly yellowish, except for dark-brown areas at abdominal sterna. Head yellow with brown markings anteriorly and posteriorly, sometimes also parallel to inner margin of eye. Eye not raised above level of vertex or pronotum. Anterior margin between eyes convex, extending anteriorly in front of eyes 24% of head length (Fig 25B). Maxillary plate broad basally, anterior edge triangular. Labrum pentagonal, distal margin tapered. Labium with article III more than twice the length of article IV (Fig 25D); article IV brown. Antenna 4-articulated, exceeding lateral margin of eye; scape bulbous, rounded; pedicel subrectangular, wider distally; flagellomeres slender, with long setae. Postgenal tubercle not reaching level of probasisternal carina. Pronotum yellow; dark-brown markings concentrated at rectangular area behind eyes; shallow sulcus marking anterior border of transverse band at posterior third. Anterior margin slightly concave between eyes; lateral margins convergent anteriorly, evenly convex; posterior margin convex, with shallow concavity medially; posterolateral corner broadly rounded. Prothorax ventrally yellow. Propleuron with shagreened area extended posteriorly along lateral margin, almost reaching posterolateral corner; posterior margin concave at midlength; posteromesal corner near prosternellum flat. Median carina of probasisternum quadrate, extending anteriorly in lateral view. Scutellum wrinkled, punctate, triangular, mostly yellow with small brown markings throughout. Hemelytra brown, lighter at embolium; membrane with small darker markings; lateral margin after embolium narrowed, exposing lateral strip of abdominal terga III–VI. Claval and intraclaval sutures absent on brachypterous specimens. Hind wings of brachypterous specimens reaching posterior margin of abdominal tergum I. Region between mesobasisternum and mesoepisternum without longitudinal row of elongated golden setae. Mesosternal carina with median ridge straight or shallowly concave (Fig 25F); fossa oval, shallow, excavated at posterior margin in lateral view. Metasternal carina oval to teardrop-shaped, depressed medially; posterior margin not excavated in lateral view (Figs 33C, E). Abdomen with lateral margins of terga III–VI exposed; terga III–VI dark-brown anterolaterally, yellow posteriorly; marginal row of short yellow setae and group of trichobothria near posterior third. Sterna yellow, except for dark-brown anterolateral corners. Sterna covered by golden pubescence, without dispersed elongate golden setae; sternum II without irregular patch or row of elongate golden setae.

#### Female terminalia.

Tergum VII width about twice length at midline; lateral margin sinuous, almost straight in basal half; posterior margin convex; lateral lobe quadrate, divergent, length twice width; posterolateral margin bearing longer setae; minute brush-like setae sometimes present (Fig 26A). Subgenital plate with longer setae on posterior margin; lateral margins slightly diverging in basal 1/3, converging in apical 2/3 to broadly rounded posterior margin. Laterosternite VII convex at mesal margin, bearing a single tuft of long setae posteriorly; lateral margin convex, shallowly concave at midlength; apex acuminate (Fig 26B). Mediotergite VIII with lateral margin concave; posterior margin broadly rounded. Laterotergite VIII with length twice greatest width; distal half of lateral margin weakly serrated, bearing minute brush-like setae (Fig 26C). Valvifer I with mesal margin convex; anterior region with few setae; posterior region bearing dense pilosity. Valvula I bearing robust, posteriorly directed setae, becoming longer towards apex; apical robust setae longer than lateral margin (Fig 26D). Valvulae II slightly wider than long, with long dense setae laterally; lateral margin convex; apex acuminate, without dark spot (Fig 26E). Valvula III thickened, halter-shaped, with dense, long pubescence basally; apex truncate, bearing dense tuft of setae directed messaly; posterior region of ventral surface with deep transverse indentation. Mediotergite IX ogival, shorter than valvula III; lateral margins converging to rounded apex; length 1.6x greatest width; at base as wide as valvula III (Fig 26F).

#### Male terminalia.

Sternum V asymmetrical, with posterior margin displaced sinistrally. Mediotergite VI with small, undeveloped accessory genitalic process, posterior margin excavated on right side. Posterior margin of mediotergite VII broadly convex; laterotergite VII slender, not surpassing level of laterotergite VI (Fig 27A). Lateral lobe of tergum VIII with lateral margin straight in anterior 3/4. Left medial lobe angled posterolaterally, posteromesal corner rounded; right medial lobe twisted laterally in apical half (Fig 27B). Pygophore with elongate golden setae densely distributed throughout surface (Figs 27D–E). Phallosoma straight, wider distally at left margin; ventral lobes membranous (Figs 27D–G). Parameres symmetrical, broad, with apical half of anterior margin straight; mesal angle narrowly rounded; setae evenly distributed throughout dorsal surface (Fig 27C).

#### Comparative notes.

*Limnocoris admontandoni* is similar to *L. insignis*, *L. rotundatus* and *L. sattleri* in general body color, shape and size. They also have similar mesosternal and metasternal carinae, and have the posterolateral angles of the pronotum slightly projected posteriorly, although in different degrees. However, in *L. admontandoni* the anterior margin of the head is strongly extended anteriorly (Fig 25B) and the female valvula III has a deeper transverse indentation at the ventral region near the posterior margin, folding over the posterior margin of the subgenital plate (Figs 25E, 26F). In the other species, the anterior margin of the head is weakly extended anteriorly and the female valvula III has a moderate to shallow diagonal indentation at the ventral region near the posterior margin (Figs 17J; 18C, D), which does not fold over the posterior margin of the subgenital plate.

#### Discussion.

La Rivers [14] described this species based on macropterous and brachypterous specimens from Jacupiranga, southeastern Brazil, and compared it with *L. montandoni* and *L. submontandoni*, which were described in the same study. He distinguished the former from the latter two by the body size, labrum shape, and abdominal structures. Nieser & Lopez-Ruf [2] examined paratypes of *L. admontandoni* and proposed the synonymy of this species with *L. insignis*, but did not provide any explanation to justify this nomenclatural act. After examining specimens collected near the type locality of *L. admontandoni*, as well as its holotype and paratypes, we found a unique combination of characteristics for this species: anterior margin of the head strongly extended anteriorly, article III of the labium more than twice the length of article IV (Fig 25D), and female valvula III with a deep transverse indentation at the ventral region near the posterior margin, causing it to fold over the posterior margin of the subgenital plate (Figs 25E, 26F), visible in undissected specimens. Therefore, *L. admontandoni* is here resurrected as a valid species.

#### Distribution.

This species is known only from a few localities in southern state of São Paulo.

***Limnocoris amazonensis* Rodrigues & Sites, 2023**

(Figs 4B, 7B, 9B, 11B, 13B, 15B, 17B, 19E–H, 23B)

*Limnocoris amazonensis* Rodrigues & Sites 2023: 47–51 (original description).

#### Type material examined.

All specimens brachypterous. HOLOTYPE: **BRAZIL, Roraima**: Caracaraí, Serra da Mocidade, Base II, Igarapé na Ilha do Pico, 01°42’23“N, 61°47’08”W, 01.ii.2016, J.M.C. Nascimento. (1♀, INPA); PARATYPE: same data as holotype (1♂, INPA).

#### Female terminalia.

Tergum VII with average width 3.5x length at mid line; lateral margin sinuous, distinctly convex in basal half; posterior margin sinuous, concave medially; lateral lobe longer than wide, bearing dense row of setae in apical half of lateral margin (Fig 7B). Subgenital plate with tuft of long setae at mid-length of lateral margin; lateral margins parallel in basal 1/3; converging to rounded apex in apical 2/3. Laterosternite VII convex on mesal margin, bearing two tufts of long setae posteriorly; proximal tuft as long as distal tuft; lateral margin slightly concave basally; apex acuminate (Fig 9B). Mediotergite VIII with lateral margin straight in apical 2/3, bearing row of dense setae, folded ventrally; posterior margin distinctly concave. Laterotergite VIII length more than twice greatest width; apical half of lateral margin serrated, bearing minute brush-like setae (Fig 11B). Valvifer I with mesal margin convex. Valvula I bearing robust, posteriorly directed setae, setae becoming longer towards apex; apical robust setae shorter than lateral margin (Fig 13B). Valvulae II slightly wider than long; lateral margin straight, with long setae; apex bearing large, faint apical dark spot (Fig 15B). Valvula III sickle-shaped, twisted in apical 2/3, with long, dense pubescence in apical half; apex narrowly rounded (in lateral view). Mediotergite IX ogival, slightly longer than valvula III; lateral margins parallel in basal half, converging in apical half to narrowly rounded apex; length twice greatest width; width at base about twice width of valvula III (Fig 17B).

#### Male genitalia.

Pygophore with elongate setae sparsely distributed over most of surface, except for a dense tuft on median region of posterior margin; anterior margin shallowly concave, posterior margin convex. Phallosoma straight (Figs 19E–H). Paramere with anterior margin straight in basal half, concave in apical half, mesal angle acute, curved ventrally and anteriorly (Fig 23B).

#### Comments.

This species has the female terminalia most similar to *L. machrisi* Nieser & López-Ruf, 2001 and *L. volxemi* (Lethierry, 1877). However, *Limnocoris amazonensis* is the only species examined in which the posterior margin of mediotergite VIII is concave (Figs 11B). It also differs from *L. machrisi* and *L. volxemi* by the absence of a cluster of short stout setae on the mesal margin of valvula I (Fig 13B). As for the male genitalia, *L. amazonensis* differs from all other species by the apical half of the paramere anterior margin distinctly concave, with the mesal angle curved ventrally and anteriorly (Fig 23B).

***Limnocoris asper* Nieser & Lopez-Ruf, 2001**

(Figs 4C, 7C, 9C, 11C, 13C, 15C, 17C, 19I–L, 23C)

*Limnocoris asper* Nieser & Lopez Ruf, 2001: 5, 7 (original description).

#### Material examined. BRAZIL, Minas Gerais.

Serra do Cipó, Travessão, C[ardeal] M[ota], [–19.33, –43.52], 02.x.1998, G.J.C. Vianna col. (2♂, 2♀ brachypterous, 1♀ macropterous, CEIOC); [Santana do Riacho], Serra do Cipó, MG-010, Km 121, [–19.26, –43.55], 07.x.1975, C.G. Froehlich col. (1♀ brachypterous, CEIOC); Santana do Riacho, Lapinha da Serra, Cachoeira do Paraíso, 19°06’28“S, 43°39’59”W, 29.i.2011, H.D.D. Rodrigues col. (1♂ macropterous, CEIOC).

#### Female terminalia.

Tergum VII with average width 3x length at midline; posterior margin sinuous; lateral margin sinuous, convex in basal half; lateral lobes longer than wide, slightly divergent (Fig 7C). Subgenital plate with uniform pilosity; lateral margins slightly diverging in basal half, converging in apical half to broadly rounded apex. Laterosternite VII convex at mesal margin, bearing single tuft of long setae posteriorly; lateral margin sinuous; apex acuminate (Fig 9C). Mediotergite VIII with lateral margin convex; posterior margin rounded. Laterotergite VIII length twice greatest width; lateral margin angulated at mid-length, apical half of lateral margin moderately serrated, bearing minute brush-like setae (Fig 11C). Valvifer I with mesal margin convex. Valvula I bearing robust, posteriorly directed setae, setae becoming longer towards apex; apical robust setae shorter than lateral margin (Fig 13C). Valvulae II slightly wider than long; lateral margin straight in apical 2/3, bearing short setae; apex narrowly rounded, bearing apical dark spot (Fig 15C). Valvula III flattened, sickle-shaped, with dense pubescence on dorsal surface; apical 2/3 medially twisted; apex narrow (in lateral view). Mediotergite IX ogival, length equal to valvula III; lateral margins convex, converging to rounded apex; length 1.5x greatest width; width at base less than twice width of valvula III (Fig 17C).

#### Male genitalia.

Pygophore with elongate setae sparsely distributed over most of surface, except for a dense tuft on median region of posterior margin; anterior margin slightly concave, posterior margin slightly convex. Phallosoma straight (Figs 19I–L). Paramere with anterior margin slightly concave in apical half; mesal angle narrowly rounded (Fig 23C).

#### Comments.

This species is most similar to *L. espinolai* Nieser & López-Ruf, 2001, with which it co-occurs at Serra do Cipó and Serra do Caraça, state of Minas Gerais. Both male and female terminalia are very similar between these species, differing only slightly in the shape of the paramere and abdominal tergum VIII of both sexes. We still refrain from synonymizing these species because they were recovered in separate clades in a recent molecular phylogeny, although with poor support [6].

***Limnocoris brasiliensis* De Carlo, 1941**

(Figs 4D, 7D, 9D, 11D, 13D, 15D, 17D, 19M–P, 24D, 28)

*Limnocoris brasiliensis* De Carlo, 1941: 37–38 (original description).

*Limnocoris bergi* De Carlo, 1941: 39–40 (original description) (synonymized by Nieser & Melo, 1999: 1235).

#### Material examined. BRAZIL, Espírito Santo.

Iúna, PARNA [Parque Nacional do] Caparaó, Trilha Pico da Bandeira, Córrego da Araucária, CAP6, 20°18’49.2“S, 41°49’33.2”W, 2109 m, 13.iv.2024, A.P. Pinto, B. Clarkson, L.H. Gil-Azevedo, L. Hoehne & N.O. Paiva col. (1♂ brachypterous, CEIOC); Iúna, PARNA Caparaó, Vale Encantado, R.[io] José Pedro, CAP1, 20°24’42.5”S, 41°49’56.7”W, 2028 m, 12.iv.2024, A.P. Pinto, B. Clarkson, L.H. Gil-Azevedo, L. Hoehne & N.O. Paiva col. (2♂, 1♀ brachypterous, CEIOC); Iúna, PARNA Caparaó, R. José Pedro, Cach.[oeira] Bonita CAP2, 20°24’21.8”S, 41°50’12.6”W, 1791 m, 16.iv.2024, A.P. Pinto, B. Clarkson, L.H. Gil-Azevedo, L. Hoehne & N.O. Paiva col. (1♂, 1♀ brachypterous, CEIOC); PN. [Parque Nacional do] Caparaó, Cach. Aurélio, CAP32, [–20.482, –41.837], 02.xii.2022, M.S.L. Alexandre col. (1♂, 4♀ brachypterous, CEIOC). **Minas Gerais**: Alto Caparaó, PARNA Caparaó, Abrigo Macieira, Cach. Sete Pilões, CAP13, 20°28’55.6”S, 41°52’51.2”W, 1880 m, 15.iv.2024, A.P. Pinto, B. Clarkson, L.H. Gil-Azevedo, L. Hoehne & N.O. Paiva col. (1♀ brachypterous, CEIOC); Alto Caparaó, PARNA Caparaó, Vale Verde, R. Caparaó, CAP3, 20°25’09.6”S, 41°50’40.4”W, 1282 m, 16.iv.2024, A.P. Pinto, B. Clarkson, L.H. Gil-Azevedo, L. Hoehne & N.O. Paiva col. (3♂, 1♀ brachypterous, CEIOC); Alto Caparaó, PARNA Caparaó, Afl.[uente] R. Caparaó, 20°25’07.0”S, 41°50’45.4”W, 1308 m, 12.iv.2024, A.P. Pinto, B. Clarkson, L.H. Gil-Azevedo, L. Hoehne & N.O. Paiva col. (5♂, 1♀ brachypterous, CEIOC); Alto Caparaó, Cach. da Farofa, 20°28’17.5”S, 41°49’40.1”W, 1950 m, 15.iv.2024, A.P. Pinto, B. Clarkson, L.H. Gil-Azevedo, L. Hoehne & N.O. Paiva col. (2♀ brachypterous, CEIOC); same data, except 30.xi.2022, A.F.A. & P.H.M.C. col. (1♂, 3♀ brachypterous, CEIOC). **Rio de Janeiro**: Itatiaia, PARNA de Itatiaia, Lago Azul, 22°16’14.1”S, 44°22’05.8”W, 801 m, 04.vi.2024, M.S.L. Alexandre col. (1♂, 5♀ brachypterous, 1♂, 2♀ macropterous, CEIOC); Itatiaia, Rio Campo Belo, 11.xii.2016, (3♀ brachypterous, 3♀ macropterous, CEIOC); Teresópolis, PARNA da Serra dos Órgãos, Rio Paquequer/ponte, 22°16’23.9”S, 42°35’41.6”W, 1135 m, 14–16.vi.2023, (1♂ brachypterous, CEIOC); Teresópolis, Parque Nacional da Serra dos Órgãos, Rio Paquequer, braço direito, 22°27’02”S, 42°59’48”W, 28.xii.2014, I.R.S. Cordeiro, F.F.F. Moreira & F.S. Motta col. (2♀ brachypterous, CEIOC).

#### Female terminalia.

Tergum VII with average width 3x length at midline, bearing long setae posteriorly; posterior margin sinuous; lateral margins sinuous, convex in basal half; lateral lobes divergent, distinctly longer than wide (Fig 7D). Subgenital plate with longest setae on lateral margin; lateral margins parallel in basal 1/3, converging in apical 2/3 to rounded apex. Laterosternite VII convex at mesal margin, bearing two tufts of long setae posteriorly; lateral margins slightly sinuous; apex narrow, with weak serration at lateral margin (Fig 9D). Mediotergite VIII with lateral margin straight in apical 2/3; posterior margin rounded. Laterotergite VIII length twice greatest width; apical half of lateral margin weakly serrated (Fig 11D). Valvifer I with mesal margin almost straight. Valvula I bearing robust, posteriorly directed setae; setae on apex about the same length as others, and shorter than lateral margin (Fig 13D). Valvulae II slightly wider than long; lateral margin slightly concave at mid length, bearing short setae; apex broadly rounded, bearing apical dark spot (Fig 15D). Valvula III flattened, sickle-shaped, with long dense pubescence on surface; apical half medially twisted; apex narrow. Mediotergite IX ogival, length equal to valvula III; lateral margins convex, converging to broadly rounded apex; length 1.5x greatest width; width at base less than twice width of valvula III (Fig 17D).

#### Male genitalia.

Pygophore with elongate setae sparsely distributed over most of surface, except for a dense tuft on median region of posterior margin; anterior margin straight, posterior margin slightly convex. Phallosoma straight (Figs 19M–P). Paramere with anterior margin straight in apical half; mesal angle rounded (Fig 23D).

#### Comments.

Specimens from Itatiaia, state of Rio de Janeiro (RJ) (Fig 28), exhibited significant variation in a few structures of the female terminalia when compared to those from Serra do Caparaó, states of Espírito Santo (ES) and Minas Gerais (MG). The subgenital plate of females from RJ have a small, rounded lobe at the posterolateral margin (Fig 28B), and the apical half of the lateral margin of laterotergite VIII is perpendicular to the central axis of the segment (Fig 28C); whereas in specimens from ES and MG, the posterolateral margin of the subgenital plate is only slightly convex (Fig 9D), and the apical half of the lateral margin of laterotergite VIII is oblique to the central axis of the segment (Fig 11D).

***Limnocoris burmeisteri* De Carlo, 1967**

(Figs 4E, 7E, 9E, 11E, 13E, 15E, 17E, 19Q–T, 23E)

*Limnocoris burmeisteri* De Carlo, 1967: 197–198 (original description).

*Limnocoris bachmanni* De Carlo, 1967: 198–199 (original description) (synonymized by Nieser & Lopez-Ruf 2001: 320).

*Limnocoris lautereri* Nieser, Chen & Melo, 2013: 336–341 (original description) (synonymized by Rodrigues & Sites 2023: 53).

#### Material examined.

All specimens brachypterous. **BRAZIL, Amazonas**: Barcelos, Serra do Aracá, Igarapé da Anta, [0.8791, –63.4540], 11.viii.2009 (2♂, 2♀, CEIOC); [Presidente Figueiredo], “Planga”, L9, Pitinguinha, [–0.78, –60.07], 22.iv.2001, D.L.V. Pereira col. (1♂, CEIOC). **Maranhão**: Caxias, Ponte, (00041)00087, [–4.9, –43.4], (1♂, LEAq). **Mato Grosso do Sul**: Bacia do Rio Iguatemi, Córrego Doradão, P3-PE (peneirão), [–23.8231, –54.3967], ii.2007, C.F.B. Floriano col. (1♂, CEIOC). **Pará**: Santarém, Igarapé do laranjal, 2°32’47.2”S, 54°55’27.9”W, 31.i.2024; (1♂, 3♀, LETIA); Oriximiná, Cachoeira [do] Jatuarana, [–1.653, –55.708], 11.vi.2019, L.A. Oliveira col. (3♂, 6♀, LETIA). **Roraima**: [Amajari], Parque Indígena Auaris, Igarapé do Posto, [4.00, –64.49], 05.iii.1994, V. Py-Daniel, U. Barbosa & Orlando col. (1♂, 1♀, CEIOC).

#### Female terminalia.

Tergum VII with average width 3x length at midline; lateral margins sinuous, convex in basal half; posterior margin sinuous; lateral lobes much longer than wide, diverging at base, converging at apex, bearing dense row of setae in basal 2/3 of lateral margin; mesal margin bearing longer sparse setae (Fig 7E). Subgenital plate with tufts of long setae in apical 2/3 of lateral margin; lateral margins parallel in basal 1/4, apical 3/4 converging to rounded apex. Laterosternite VII convex on mesal margin, bearing two tufts of long setae posteriorly; proximal tuft longer than distal tuft; lateral margins slightly sinuous; apex narrow, acute (Fig 9E). Mediotergite VIII with lateral margin convex, ventrally folded; posterior margin broadly rounded. Laterotergite VIII length less than twice its width; apical half of lateral margin moderately serrated, converging posteriorly, bearing minute brush-like setae (Fig 11E). Valvifer I with mesal margin convex. Valvula I bearing robust, posteriorly directed setae, setae becoming longer towards apex; apical robust setae shorter than lateral margin (Fig 13E). Valvulae II slightly wider than long; lateral margin slightly convex, bearing long setae; apex broadly rounded, apical dark spot sometimes present (Fig 15E). Valvula III flattened, sickle-shaped; apical 1/3 medially reflected, with long, dense pubescence on apical half; apex rounded. Mediotergite IX ogival, longer than valvula III; lateral margins convex, converging to narrowly rounded apex; length twice greatest width; width at base less than twice width of valvula III (Fig 17E).

#### Male genitalia.

Pygophore with elongate setae sparsely distributed over most of surface, except for a dense tuft on median region of posterior margin; anterior margin slightly concave, posterior margin slightly convex. Phallosoma straight, with apex slightly bent dextrally in dorsal view (Figs 19Q–T). Paramere with anterior margin straight in apical half; mesal angle broadly rounded (Fig 23E).

#### Comments.

This species shares some similarities in the female terminalia with those of *L. machrisi*, *L. melloleitaoi* De Carlo, 1951, *L. menkei* La Rivers, 1962, *L. minutus* De Carlo, 1951 and *L. volxemi*, such as: the posterior margin of tergum VII sinuous; the subgenital plate with long golden setae on the lateral margin; and valvulae III with long dense pubescence on the apical half. However, it can be distinguished from the others by the lateral lobes of tergum VII converging apically (Fig 7E), and valvulae II wider than long (Fig 17E). As for the male genitalia, no structure is modified enough to distinguish *L. burmeisteri* from any other species.

***Limnocoris decarloi* Nieser & Lopez-Ruf, 2001**

(Figs 4F, 7F, 9F, 11F, 13F, 15F, 17F, 19U–X, 23F, Fig 29, Fig 30)

*Limnocoris sattleri* De Carlo, 1966: 113 (original description) (paratypes from Rio Grande do Sul, partim).

*Limnocoris decarloi* Nieser & Lopez-Ruf, 2001: 303–305 (original description).

*Limnocoris intermedius* Nieser & Lopez-Ruf, 2001: 308–309 (original description) (**new synonym**).

*Limnocoris lanemeloi* Nieser & Lopez-Ruf, 2001: 309–311 (original description) (**new synonym**).

#### Type material examined.

All specimens brachypterous. HOLOTYPE of *L. decarloi*, ♂ (ZSMC), [**BRAZIL**], Brasilien, Cipo, **Rio Grande do Sul**, 10.9–8.12.1960, C. Ribeiro leg./ *Limnocoris sattleri* De Carlo, Museo Argentino de Ciencias Naturales/ Paratypus ♂/ *Limnocoris decarloi* Nsr & L-Ruf, holotype ♂/ Sammlg. H. Weber. PARATYPES of *L. decarloi*: same data as holotype, except: *Limnocoris sattleri* De Carlo det. De Carlo/ Paratypus/ *Limnocoris decarloi* Nsr. L-Ruf, allotype ♀ (1♀ ZSMC); same data as holotype, except: J.T. Polhemus Collection 2014, C.J. Drake Accession/ *Limnocoris decarloi* Nsr. & L. Ruf, paratype (1♂, 2♀ USNM); same data as holotype, except: *Limnocoris sattleri* De Carlo, Museo Argentino de Ciencias Naturales/ J.T. Polhemus Collection 2014, C.J. Drake Accession/ *Limnocoris decarloi* Nsr. & L. Ruf, paratype (1♂ USNM); [**BRAZIL**, **Santa Catarina**], Rio Piurras, 27°48[’S], 49°55[’W], 700 m, Bocaina, Dez. 1962, F. Plaumann/ *Limnocoris decarloi* Nsr. & L. Ruf, paratype (1♂ MLP, 2944). HOLOTYPE of *L. intermedius*, ♀ (NCTN), [**BRAZIL**, **Paraná**], Rio Capivari, 25°15[S], 49°07[W], 1000 m, Bocaiuva, V.1964, (PA.), F. Plaumann/ Holotype ♀ 2001 *Limnocoris intermedius* Nieser & Lopez-Ruf/ NCTN/ RMHS.INS. 1485658. HOLOTYPE of *L. lanemeloi*, ♂ (DPIC), [**BRAZIL**, **Minas Gerais**], Brasil, MG, S. Roque de Minas, Rio do Peixe [20°14’35”S, 46°22’13W], 27/ III.1996, leg. Nieser N9636, mountain stream intown/ *Limnocoris lanemeloi* Nsr & LR, Holotype ♂/ DPIB/ 3561. PARATYPES of *L. lanemeloi*: same data, except: *Limnocoris lanemeloi* Nsr & LR, Paratype (1♀ ‘allotype’ DPIC; 1♂, 1♀ MLP: 2940/1, 2940/2); same data as holotype, except: J.T. Polhemus Collection 2014, C.J. Drake Accession (1♂, 1♀ USNM).

#### Additional material examined. BRAZIL, Minas Gerais.

São Roque de Minas, Rio do Peixe, rock & gravel riffles, 20°14.607’S, 46°22.070’W, 29.xi.2016, H.D.D. Rodrigues col. (1♂, 1♀ brachypterous, CEIOC). **Paraná**: Morretes, Rio Nhundiaquara, 25°27’28.9“S, 48°5004.8”W, 28.x.2023, 14 m, R.P.R. Canejo, J.M.S. Rodrigues, M.S.L. Alexandre & L.D. Pereira col. (42♂, 49♀ brachypterous, 4♂, 2♀ macropterous, CEIOC); same data, except 18.vii.2015, P. Camelier col. (1♂, 1♀ brachypterous, CEIOC). **São Paulo**: Ribeirão Grande, SP39, Rio das Almas, cachoeira, 24°09’26.6”S, 48°21’07.6”W, 699 m, 05.xii.2023, L.L. Dumas, J.M.S. Rodrigues, L. Nery & L. Hoehne col. (8♂, 11♀ brachypterous, CEIOC); Iporanga, PETAR [Parque Estadual Turístico do Alto Ribeira], Bairro da Serra, Nascente do Rio Areias, 24°39’51.7”S, 48°40’15.9”W, 193 m, 13.vi.2024, A.C. Passos, E.A.J. Joaquim, M.M.A. Laurindo & N.O. Paiva col. (1♀ macropterous, CEIOC).

#### Female terminalia.

Tergum VII with average width 2.5x length at midline; lateral margin sinuous, convex in basal half; posterior margin convex; lateral lobes diverging, slightly longer than wide (Fig 7F). Mediosternite VII (subgenital plate) with uniform pilosity; lateral margins parallel in basal 1/3; apical 2/3 slightly convex at mid length, converging to rounded apex. Laterosternite VII convex at mesal margin, bearing single tuft of long setae posteriorly; lateral margins slightly sinuous; apex acute (Fig 9F). Mediotergite VIII with lateral margin straight; posterior margin rounded. Laterotergite VIII length less than twice its width; apical half of lateral margin weakly serrated, converging posteriorly, bearing minute brush-like setae (Fig 11F). Valvifer I with mesal margin convex. Valvula I bearing robust, posteriorly directed setae, setae becoming longer towards apex; apical robust setae longer than lateral margin (Fig 13F). Valvulae II slightly wider than long; lateral margin convex, bearing long setae, converging posteriorly to narrowly rounded apex; apical dark spot absent (Fig 15F). Valvula III thickened, sickle-shaped, with long, dense pubescence on surface; posterior half medially twisted; apex narrowly rounded. Mediotergite IX ogival, shorter than valvula III; lateral margins convex, converging to acute apex; length 1.4x greatest width; width at base equal to width of valvula III (Fig 17F).

#### Male genitalia.

Pygophore with elongate setae sparsely distributed over most of surface, except for a dense tuft on median region of posterior margin; anterior and posterior margins straight. Phallosoma straight (Figs 19U–X). Paramere with anterior margin straight in apical half; mesal angle rounded (Fig 24F).

#### Comments.

*Limnocoris decarloi*, *L. intermedius* Nieser & Lopez-Ruf, 2001 and *L. lanemeloi* Nieser & Lopez-Ruf, 2001 were described in the same study by Nieser & Lopez-Ruf [2] and are morphologically very similar. However, only *L. lanemeloi* and *L. intermedius* were briefly compared to each other in the original description, differing only in body length and by minor variations in the morphology of the hind femur and female subgenital plate. Also, some of the specimens mentioned in the original description of *L. decarloi* were not included in the type series because they presented longer body lengths (mean of 9.3 mm for males and 9.4 mm for females), measurements close to the mean recorded for *L. intermedius* (9.5 mm for both sexes), and came from the type locality of the latter. We recently examined specimens of *L. decarloi* collected in the state of São Paulo, which showed a darker overall coloration and longer mean length (9.3 mm for males; 9.5 mm for females) compared to specimens from other localities (Fig 30D). After examining type specimens of the three species, including the holotype, and studying the terminalia of additional specimens collected near the type localities, we found no significant morphological differences to justify the recognition of *L. intermedius* and *L. lanemeloi* as distinct species. Therefore, both are herein proposed as junior synonyms of *L. decarloi*.

The male and female terminalia of *L. decarloi* share a few similarities with other species, such as *L. curvipenis* **n. sp.**, *L. sitesi* **n. sp.** and *L. submontandoni* La Rivers, 1974. The shape of female abdominal tergum VII and all structures of the male terminalia of *L. decarloi* are very similar to those of *L. sitesi* **n. sp.**. However, they can be distinguished by the presence of a fringe of golden setae on the posterolateral margins of the subgenital plate in the latter (Fig 37B) (absent in the former), and the apex of valvulae III asymmetrical in the latter (Fig 37E) (symmetrical in the former). Valvula III of this species is conical, as in *L. curvipenis* **n. sp.** (Fig 34F) and *L. submontandoni* (Fig 18F); however, they can be distinguished by the shape of the female abdominal tergum VII and the subgenital plate.

**Fig 6:**
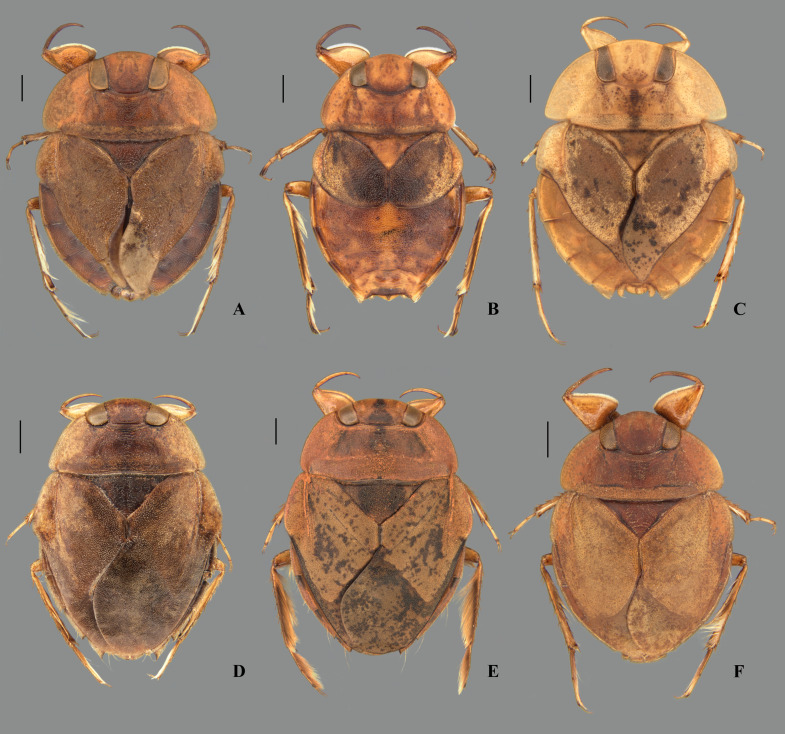
Dorsal habitus of *Limnocoris* spp. **(A)** *Limnocoris sattleri*, **(B)** *L. siolii*, **(C)** *L. submontandoni*, **(D)** *L. surinamensis*, **(E)** *L. volxemi*, **(F)** *L. yanomami*. Scale bar = 1 mm.

**Fig 7:**
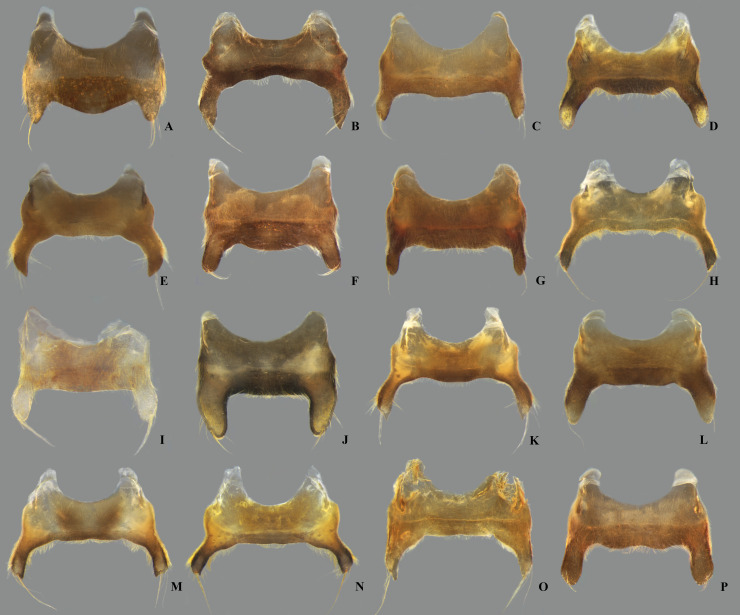
Dorsal view of female abdominal tergum VII of *Limnocoris.* **(A)** *Limnocoris acutalis*, **(B)** *L. amazonensis*, holotype, **(C)** *L. asper*, **(D)** *L. brasiliensis*, **(E)** *L. burmeisteri,* **(F)** *L. decarloi*, **(G)** *L. espinolai*, **(H)** *L. fittkaui*, **(I)** *L. illiesi*, **(J)** *L. insignis*, **(K)** *L. machrisi*, **(L)** *L. melloleitaoi*, **(M)** *L. menkei*, **(N)** *L. minutus*, **(O)** *L. moreirai* and **(P)** *L. pauper*.

**Fig 8:**
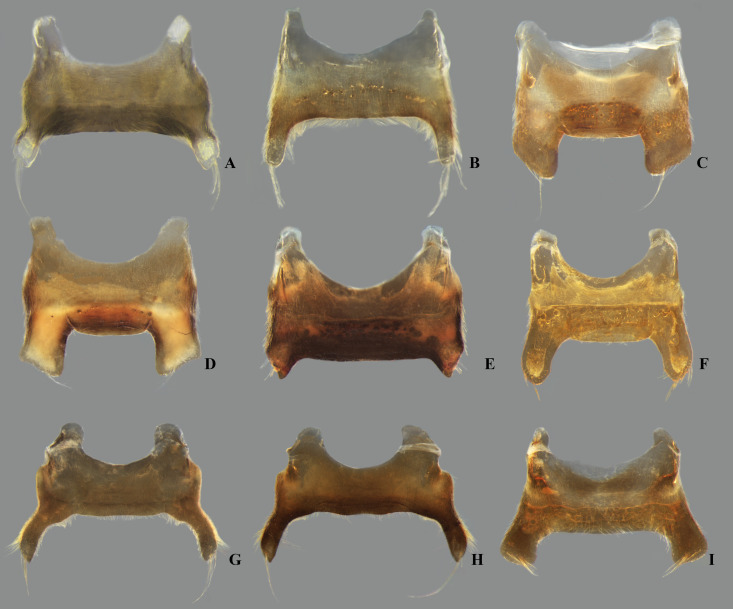
Female abdominal tergum VII of *Limnocoris.* **(A)** *Limnocoris pusillus*, **(B)** *L. reynosoi*, **(C)** *L. rotundatus*, **(D)** *L*. *sattleri*, **(E)** *L. siolii*, **(F)** *L. submontandoni*, **(G)** *L. surinamensis*, **(H)** *L. volxemi*, **(I)** *L. yanomami*.

**Fig 9:**
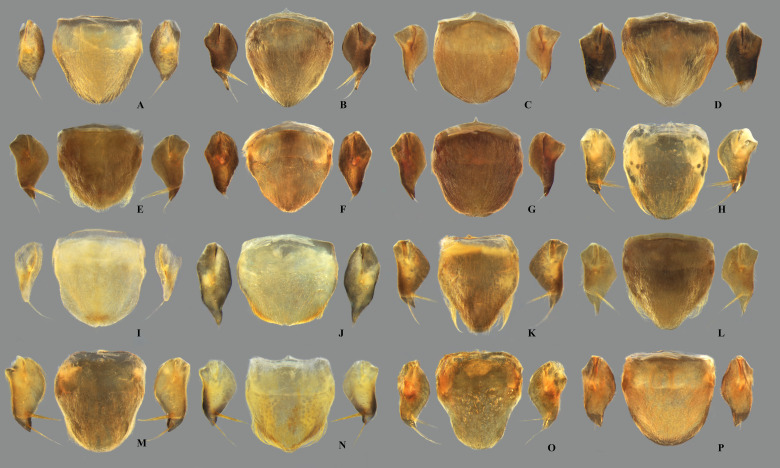
Female abdominal mediosternite (subgenital plate) and laterotergites VII of *Limnocoris.* **(A)** *Limnocoris acutalis*, **(B)** *L. amazonensis*, holotype **(C)** *L. asper*, **(D)** *L. brasiliensis*, **(E)** *L. burmeisteri*, **(F)** *L. decarloi*, **(G)** *L. espinolai*, **(H)** *L. fittkaui*, **(I)** *L. illiesi*, **(J)** *L. insignis*, **(K)** *L. machrisi*, **(L)** *L. melloleitaoi*, **(M)** *L. menkei*, **(N)** *L. minutus*, **(O)** *L. moreirai*, paratype and **(P)** *L. pauper*.

**Fig 10:**
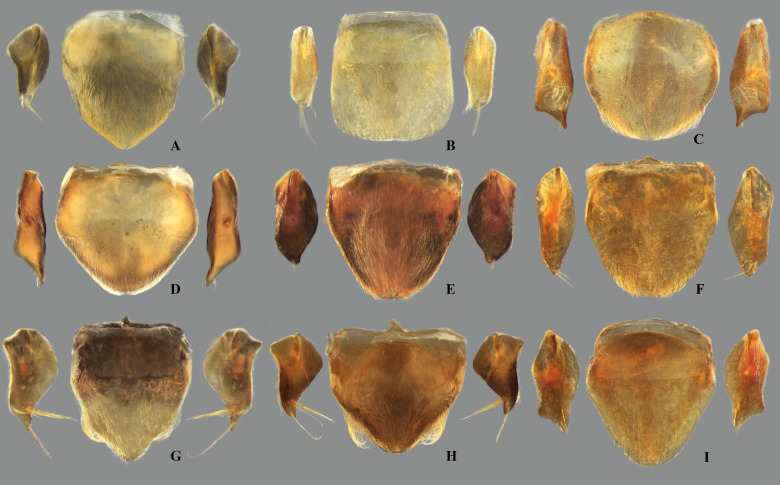
Female abdominal mediosternite (subgenital plate) and laterotergites VII of *Limnocoris.* **(A)** *L. pusillus*, **(B)** *L. reynosoi*, **(C)** *L. rotundatus*, **(D)** *L*. *sattleri*, **(E)** *L. siolii*, **(F)** *L. submontandoni*, **(G)** *L. surinamensis*, **(H)** *L. volxemi*, **(I)** *L. yanomami*.

**Fig 11:**
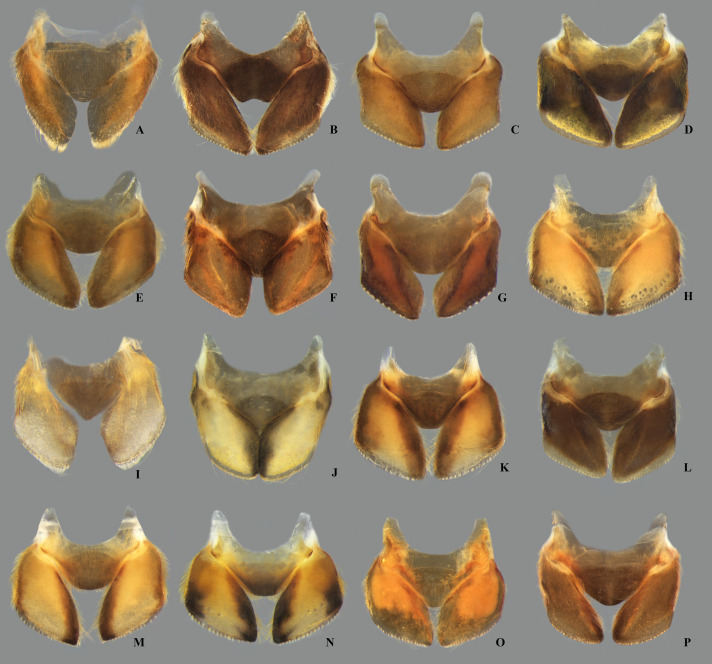
Female abdominal tergum VIII of *Limnocoris.* **(A)** *Limnocoris acutalis*, **(B)** *L. amazonensis*, holotype, **(C)** *L. asper*, **(D)** *L. brasiliensis*, **(E)** *L. burmeisteri*, **(F)** *L. decarloi*, **(G)** *L. espinolai*, **(H)** *L. fittkaui*, **(I)** *L. illiesi*, **(J)** *L. insignis*, **(K)** *L. machrisi*, **(L)** *L. melloleitaoi*, **(M)** *L. menkei*, **(N)** *L. minutus*, **(O)** *L. moreirai*, paratype and **(P)** *L. pauper*.

**Fig 12:**
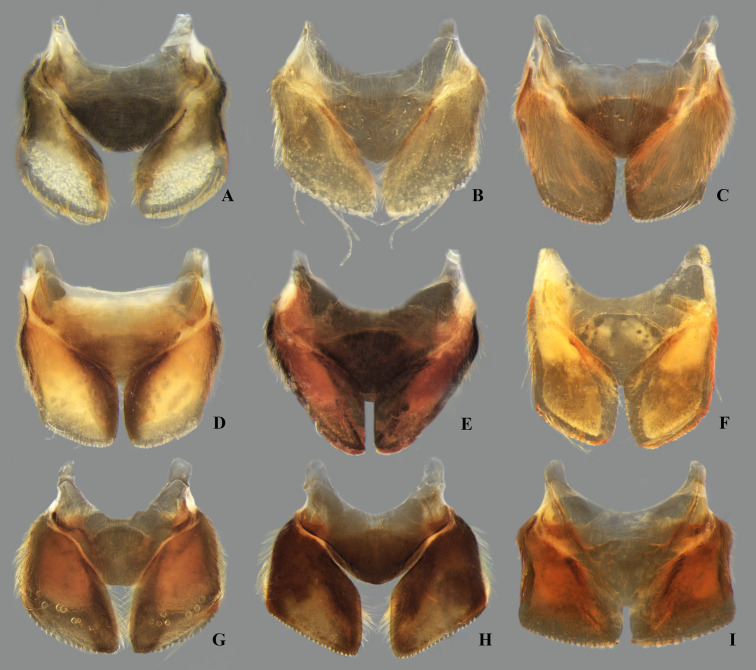
Female abdominal tergum VIII of *Limnocoris.* **(A)** *Limnocoris pusillus*, **(B)** *L. reynosoi*, **(C)** *L. rotundatus*, **(D)** *L*. *sattleri*, **(E)** *L. siolii*, **(F)** *L. submontandoni*, **(G)** *L. surinamensis*, **(H)** *L. volxemi*, **(I)** *L. yanomami*.

**Fig 13:**
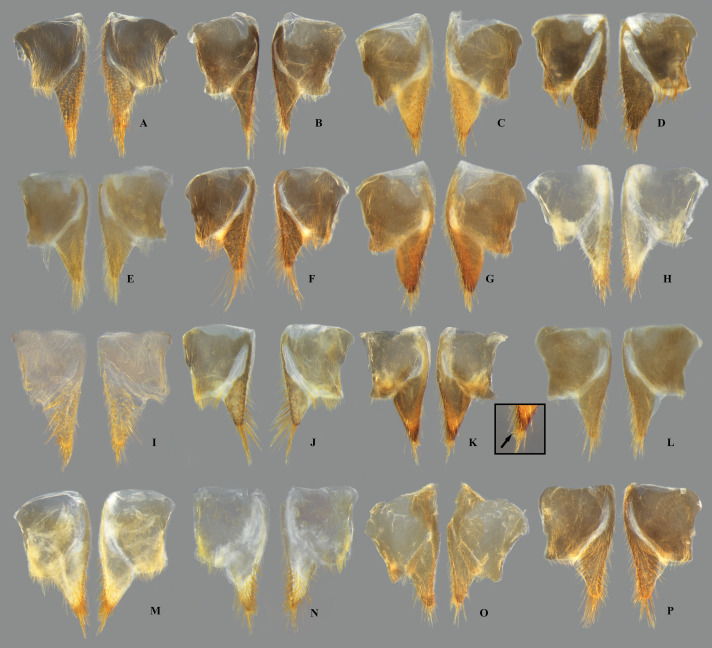
Female valvifers I + valvulae I of *Limnocoris.* **(A)** *Limnocoris acutalis*, **(B)** *L. amazonensis*, holotype, **(C)** *L. asper*, **(D)** *L. brasiliensis*, **(E)** *L. burmeisteri*, **(F)** *L. decarloi*, **(G)** *L. espinolai*, **(H)** *L. fittkaui*, **(I)** *L. illiesi*, **(J)** *L. insignis*, **(K)** *L. machrisi*, arrow in inset showing cluster of short setae, **(L)** *L. melloleitaoi*, **(M)** *L. menkei*, **(N)** *L. minutus*, **(O)** *L. moreirai*, paratype and **(P)** *L. pauper*.

**Fig 14:**
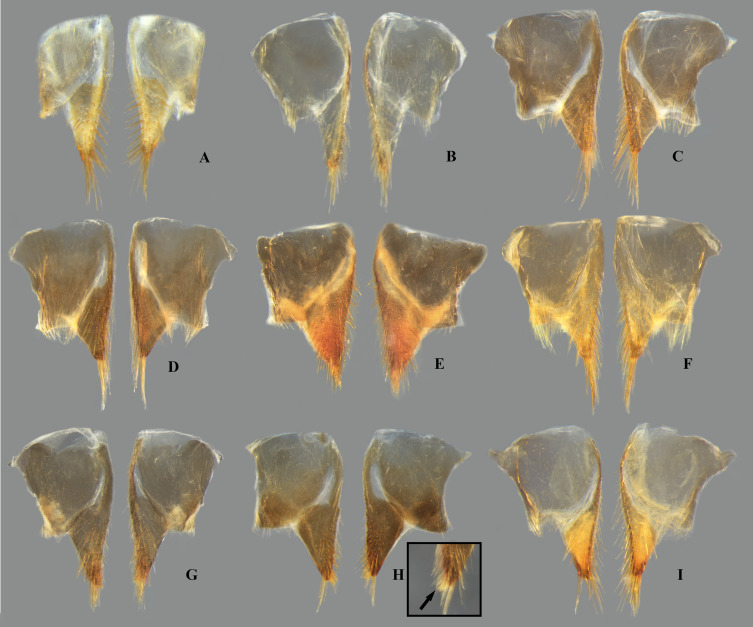
Female valvivers I + valvulae I of *Limnocoris.* **(A)** *Limnocoris pusillus*, **(B)** *L. reynosoi*, **(C)** *L. rotundatus*, **(D)** *L*. *sattleri*, **(E)** *L. siolii*, **(F)** *L. submontandoni*, **(G)** *L. surinamensis*, **(H)** *L. volxemi*, arrow in inset showing cluster of short setae, **(I)** *L. yanomami*.

**Fig 15:**
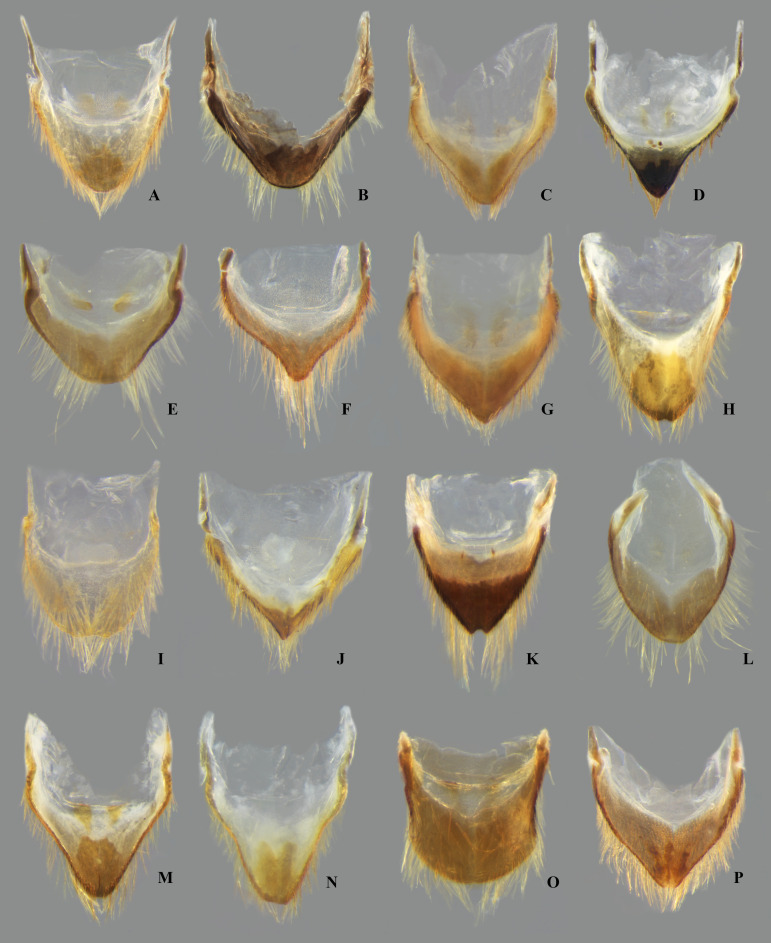
Female valvulae II of *Limnocoris.* **(A)** *Limnocoris acutalis*, **(B)** *L. amazonensis*, holotype, **(C)** *L. asper*, **(D)** *L. brasiliensis*, **(E)** *L. burmeisteri*, **(F)** *L. decarloi*, **(G)** *L. espinolai*, **(H)** *L. fittkaui*, **(I)** *L. illiesi*, **(J)** *L. insignis*, **(K)** *L. machrisi*, **(L)** *L. melloleitaoi*, **(M)** *L. menkei*, **(N)** *L. minutus*, **(O)** *L. moreirai*, paratype and **(P)** *L. pauper*.

**Fig 16:**
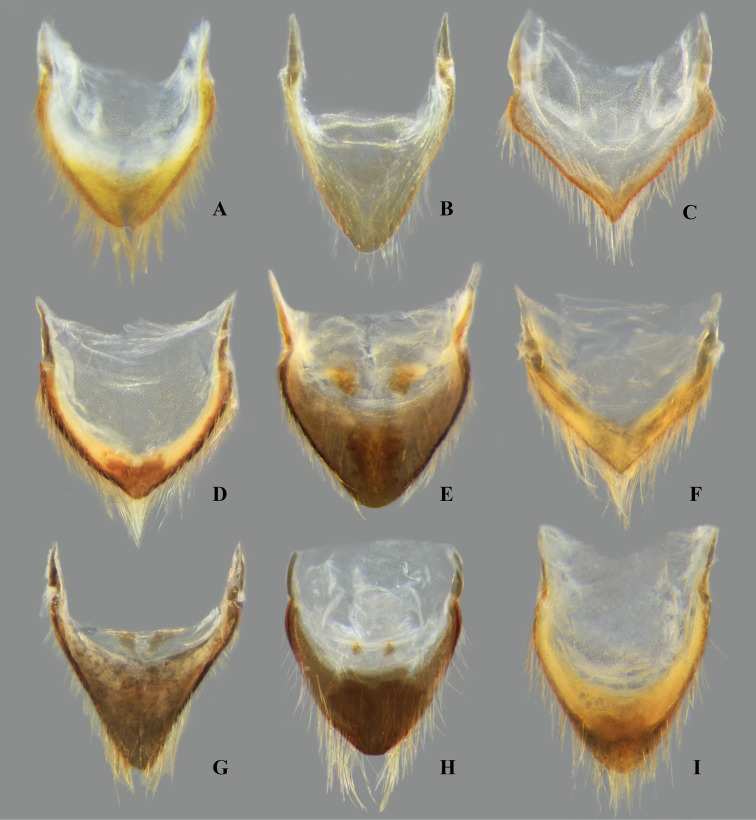
Female valvulae II of *Limnocoris.* **(A)** *Limnocoris pusillus*, **(B)** *L. reynosoi*, **(C)** *L. rotundatus*, **(D)** *L*. *sattleri*, **(E)** *L. siolii*, **(F)** *L. submontandoni*, **(G)** *L. surinamensis*, **(H)** *L. volxemi*, **(I)** *L. yanomami*.

**Fig 17:**
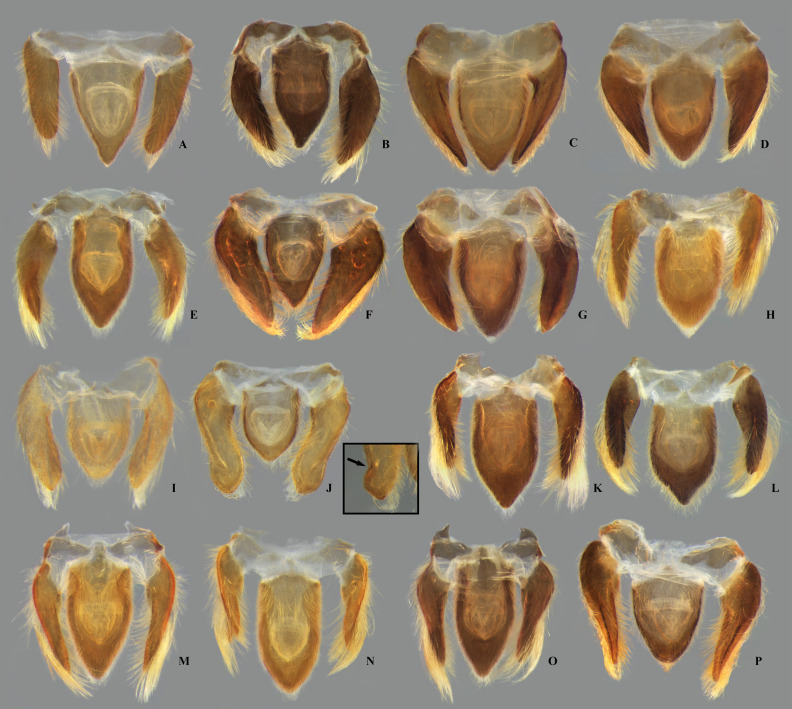
Female valvifers II + valvulae III + mediotergite IX of *Limnocoris.* **(A)** *Limnocoris acutalis*, **(B)** *L. amazonensis*, holotype, **(C)** *L. asper*, **(D)** *L. brasiliensis*, **(E)** *L. burmeisteri*, **(F)** *L. decarloi*, **(G)** *L. espinolai*, **(H)** *L. fittkaui*, **(I)** *L. illiesi*, **(J)** *L. insignis*, arrow in inset showing indentation, **(K)** *L. machrisi*, **(L)** *L. melloleitaoi*, **(M)** *L. menkei*, **(N)** *L. minutus*, **(O)** *L. moreirai*, paratype and **(P)** *L. pauper*.

**Fig 18:**
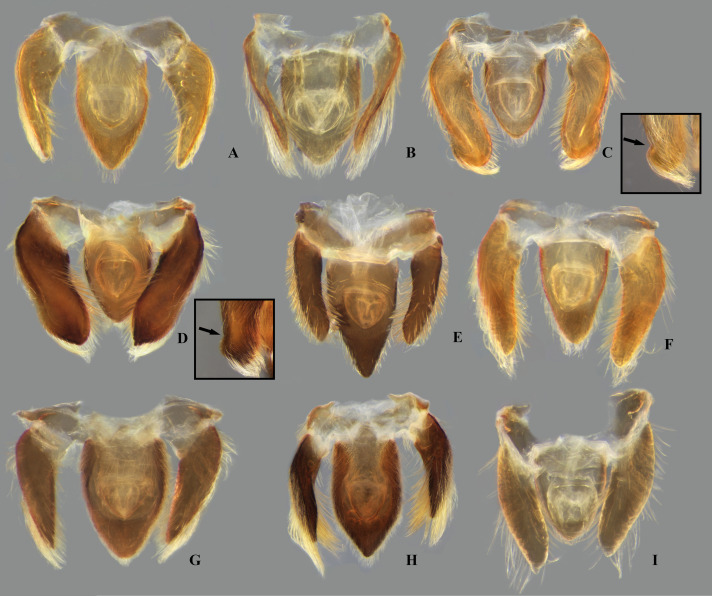
Female valvifers II + valvulae III + mediotergite IX of *Limnocoris.* **(A)** *Limnocoris pusillus*, **(B)** *L. reynosoi,* **(C)** *L. rotundatus*, arrow in inset showing indentation, **(D)** *L*. *sattleri*, arrow in inset showing shallow indentation, **(E)** *L. siolii*, **(F)** *L. submontandoni*, **(G)** *L. surinamensis*, **(H)** *L. volxemi*, **(I)** *L. yanomami*.

**Fig 19:**
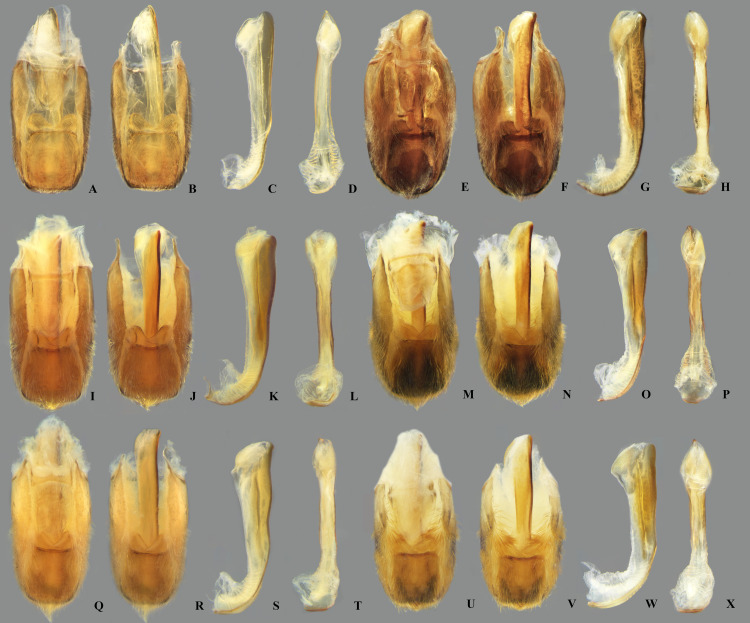
Structures of the male genital capsule of *Limnocoris.* Genital capsule with proctiger, genital capsule with proctiger removed, phallosoma in lateral view and phallosoma in ventral view of **(A–D)** *L. acutalis*, **(E–H)** *L. amazonensis*, holotype, **(I–L)** *L. asper*, **(M–P)** *L. brasiliensis*, **(Q–T)** *L. burmeisteri* and **(U–X)** *L. decarloi*.

**Fig 20:**
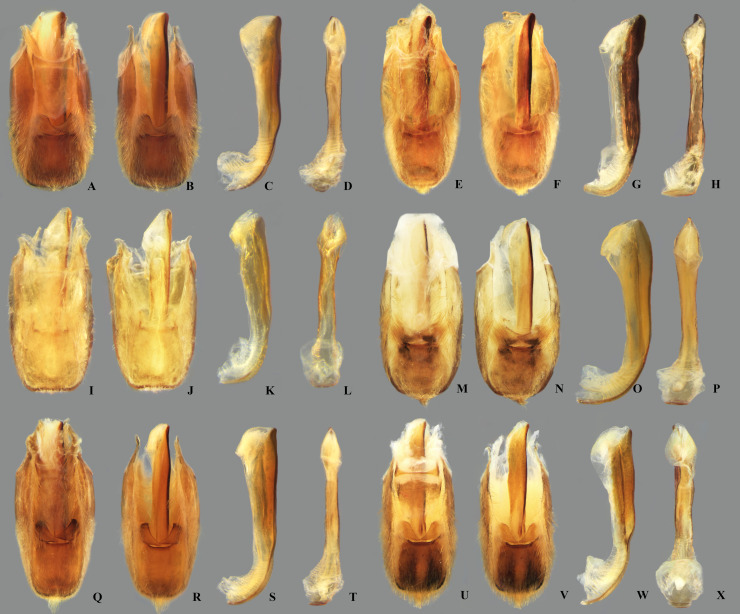
Structures of the male genital capsule of *Limnocoris.* Genital capsule with proctiger, genital capsule with proctiger removed, phallosoma in lateral view and phallosoma in ventral view of **(A–D)** *L. espinolai*, **(E–H)** *L. fittkaui*, **(I–J)** *L. illiesi*, **(M–P)** *L. insignis* **(Q–T)**, *L. machrisi* and **(U–X)** *L. melloleitaoi*.

**Fig 21:**
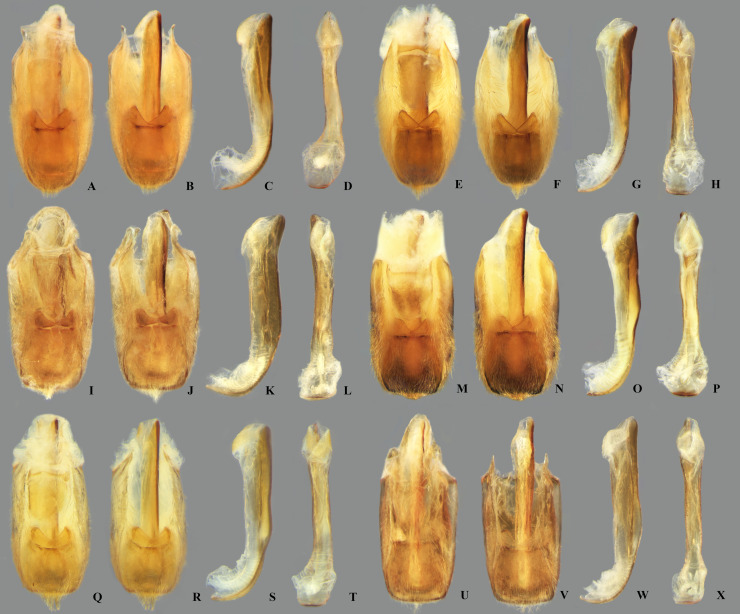
Structures of the male genital capsule of *Limnocoris.* Genital capsule with proctiger, genital capsule with proctiger removed, phallosoma in lateral view and phallosoma in ventral view of **(A–D)** *L. menkei*, **(E–H)** *L. minutus*, **(I–L)** *L. moreirai*, paratype, **(M–P)** *L. pauper*, **(Q–T)** *L. pusillus* and **(U–X)** *L. reynosoi*.

**Fig 22:**
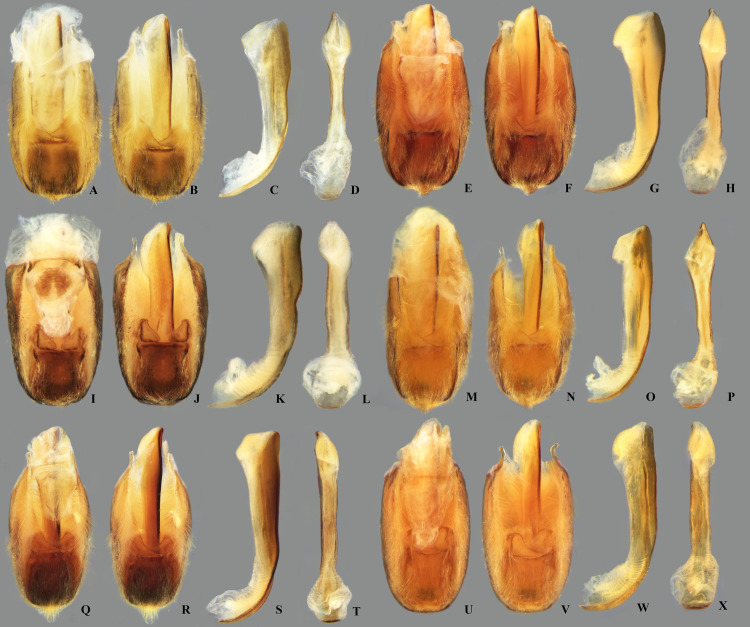
Structures of the male genital capsule of *Limnocoris.* Genital capsule with proctiger, genital capsule with proctiger removed, phallosoma in lateral view and phallosoma in ventral view of **(A–D)** *L. rotundatus*, **(E–H)** *L. sattleri*, **(I–L)** *L. siolii*, **(M–P)** *L. submontandoni*, **(Q–T)** *L. volxemi* and **(U–X)** *L. yanomami*.

**Fig 23:**
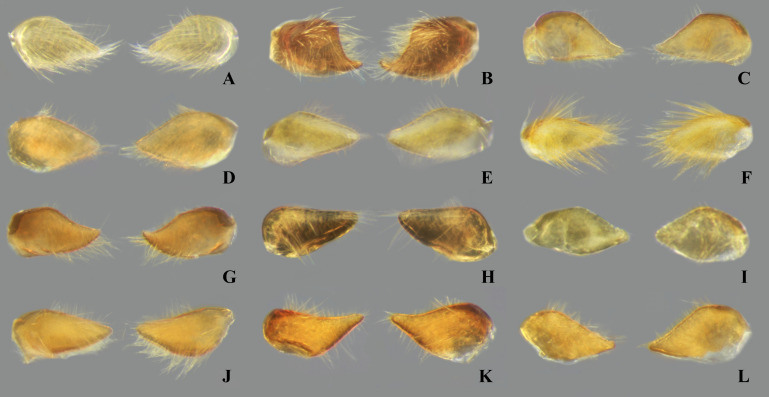
Dorsal view of parameres of *Limnocoris.* **(A)** *Limnocoris acutalis*, **(B)** *L. amazonensis*, holotype, **(C)** *L. asper*, **(D)** *L. brasiliensis*, **(E)** *L. burmeisteri*, **(F)** *L. decarloi*, **(G)** *L. espinolai*, **(H)** *L. fittkaui*, **(I)** *L. illiesi*, **(J)** *L. insignis*, **(K)** *L. machrisi* and **(L)** *L. melloleitaoi*.

**Fig 24:**
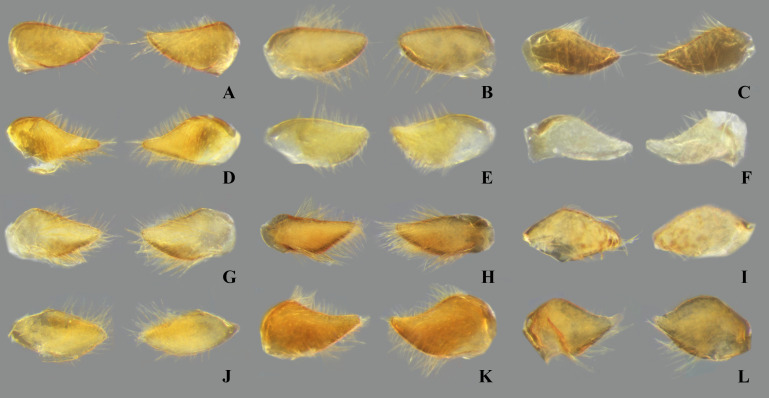
Dorsal view of parameres of *Limnocoris.* **(A)** *Limnocoris menkei*, **(B)** *L. minutus*, **(C)** *L. moreirai*, paratype, **(D)** *L. pauper*, **(E)** *L. pusillus*, **(F)** *L. reynosoi*, **(G)** *L. rotundatus*, **(H)** *L*. *sattleri*, **(I)** *L. siolii*, **(J)** *L. submontandoni*, **(K)** *L. volxemi*, **(L)** *L. yanomami*.

**Fig 25:**
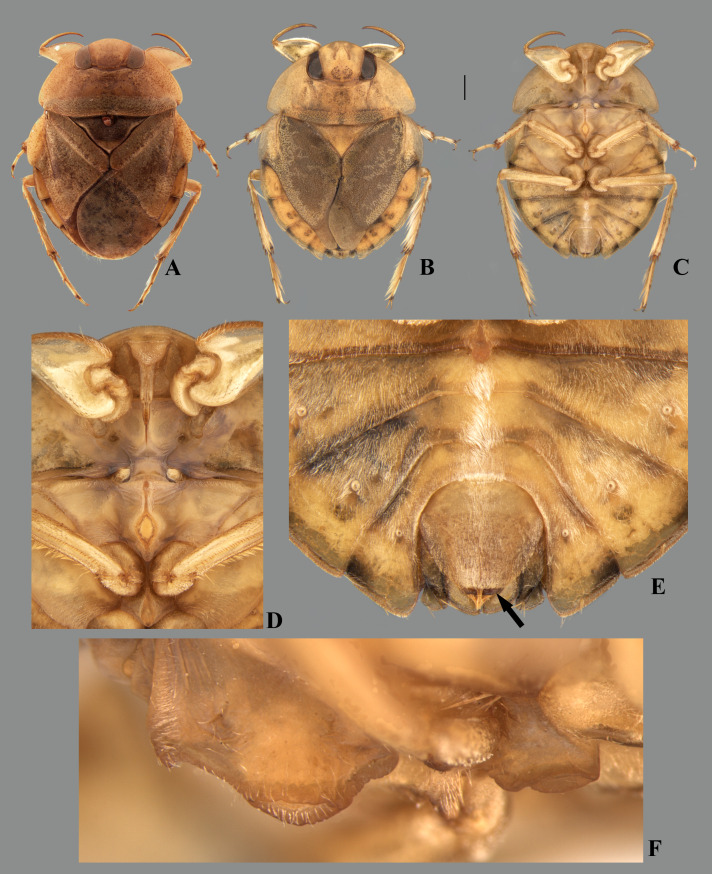
*Limnocoris admontandoni* La Rivers. stat. restit. **(A)** Dorsal habitus of female paratype, hind wing macropterous (CAS) **(B)** dorsal habitus of female, hind wing brachypterous, **(C)** ventral habitus of female, hind wing brachypterous **(D)** ventral view of meso- and metasternal carinae, **(E)** ventral view of apex of female abdomen. Arrow indicates protuding apex of valvulae III folding over the posterior margin of subgenital plate, and **(F)** lateral view of meso- and metasternal carinae. Images not to scale. Scale bar = 1 mm, for B and C only.

**Fig 26:**
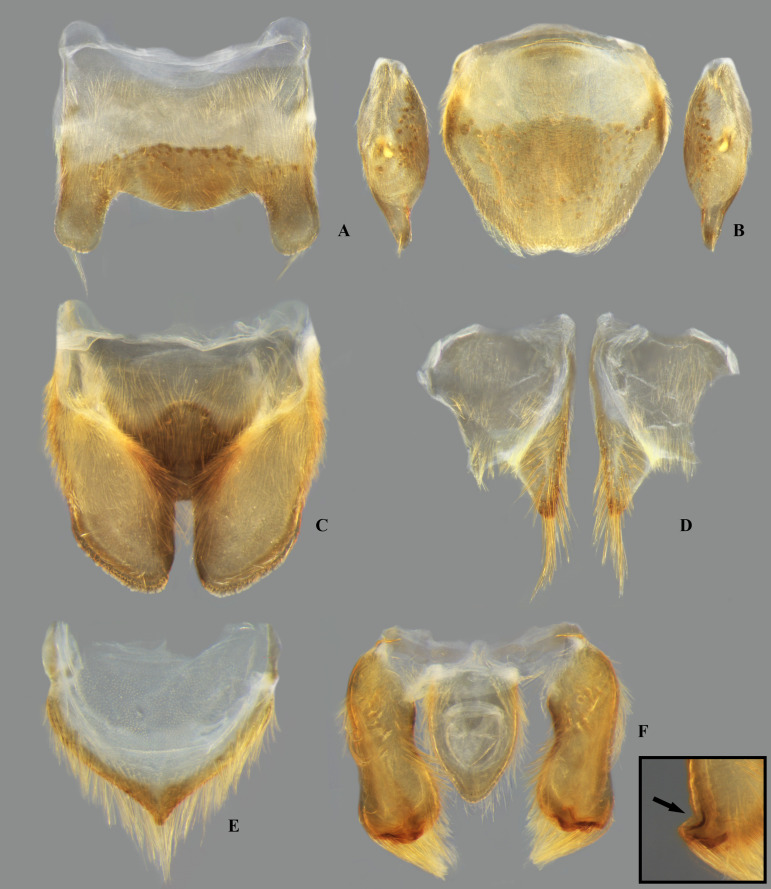
*Limnocoris admontandoni* La Rivers. stat. restit. Female terminalia. **(A)** Abdominal tergum VII, **(B)** abominal mediosternite VII (subgenital plate) + laterosternites VII, **(C)** abdominal tergum VIII, **(D)** Valvifer I + Valvulae I, **(E)** Valvulae II, **(F)** Valvifer II + Valvulae III + abdominal mediotergite IX, inset showing apex of valvula III in lateral view, arrow indicates deep indentation. Images not to scale.

**Fig 27:**
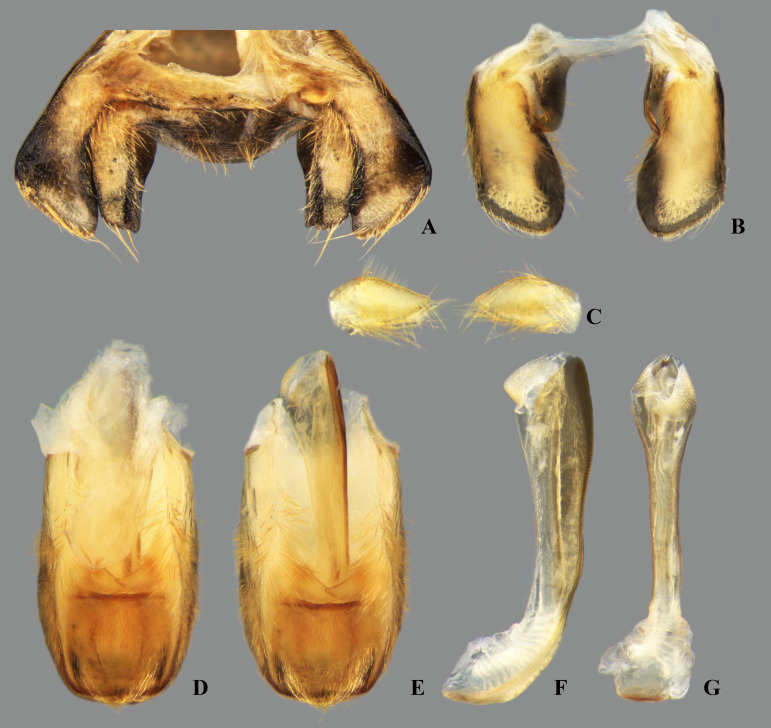
*Limnocoris admontandoni* La Rivers. stat. restit. Male terminalia. **(A)** Abdominal terga VI + VII, **(B)** abdominal tergum VIII, **(C)** parameres, **(D)** genital capsule, **(E)** genital capsule (proctiger removed) showing phallosoma in dorsal view, **(F)** phallosoma in lateral view and **(G)** phallosoma in ventral view. Images not to scale.

**Fig 28:**
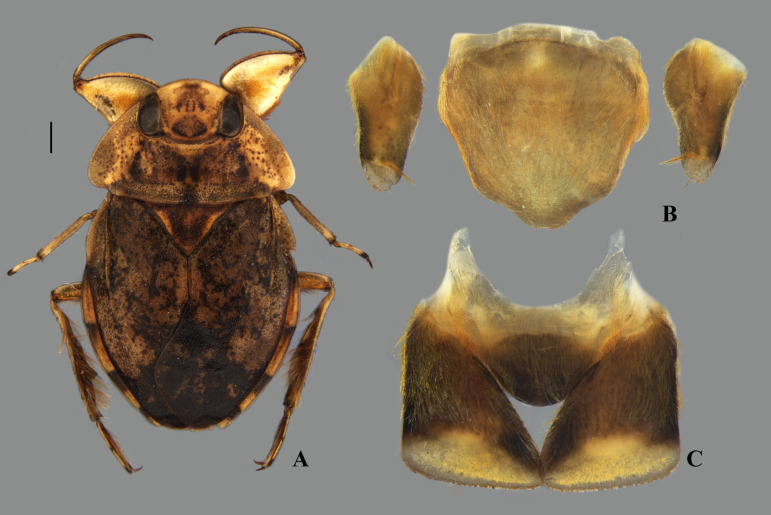
*Limnocoris brasiliensis* from Itatiaia National Park, state of Rio de Janeiro. **(A)** Dorsal habitus, **(B)** abominal mediosternite VII (subgenital plate) + laterosternites VII and **(C)** abdominal tergum VIII. Scale bar = 1 mm, for (A) only.

**Fig 29:**
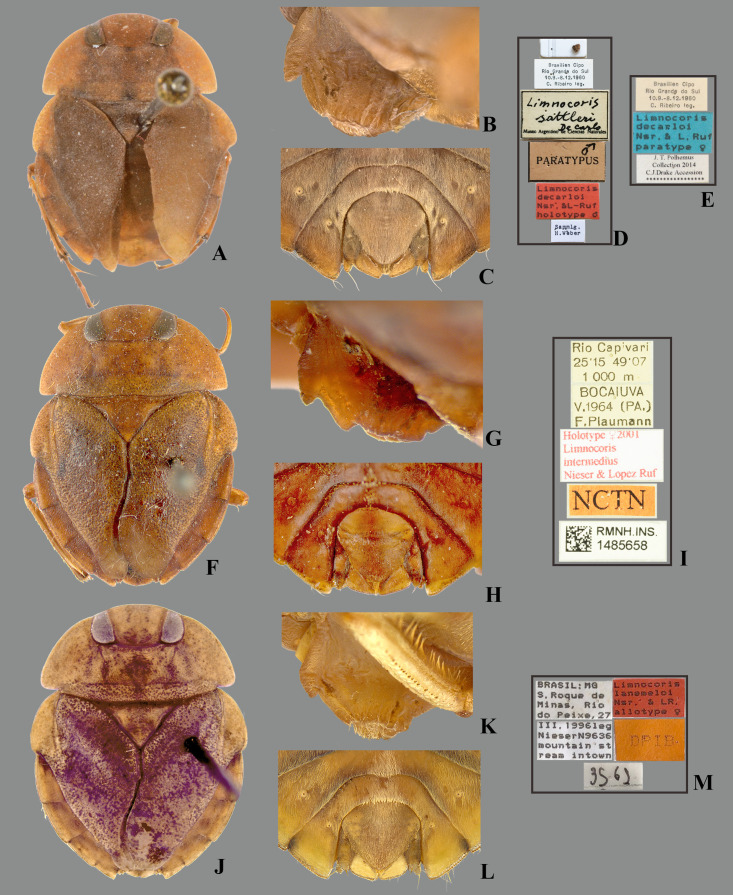
*Limnocoris decarloi* Nieser & López-Ruf. **(A)** Dorsal habitus of male holotype, hind wing brachypterous (ZSMC), **(B–C)** female paratype, hind wing brachypterous (USNM) **(B)** lateral view of mesosternal carina of female paratype, **(C)** terminal abdominal sterna of female paratype, **(D)** holotype labels, **(E)** paratype labels; **(F–H)** *Limnocoris intermedius* Nieser & López-Ruf, female holotype, hind wing brachypterous **n. syn.** (NCTN), **(F)** dorsal habitus, **(G)** lateral view of mesosternal carina, **(H)** terminal abdominal sterna, **(J–K)** *Limnocoris lanemeloi* Nieser & López-Ruf **n. syn.** **(J)** dorsal habitus of female ‘allotype’ (DPIC), **(K)** lateral view of mesosternal carina of female topotype, **(L)** terminal abdominal sterna of female topotype, **(M)** ‘allotype’ labels.

**Fig 30:**
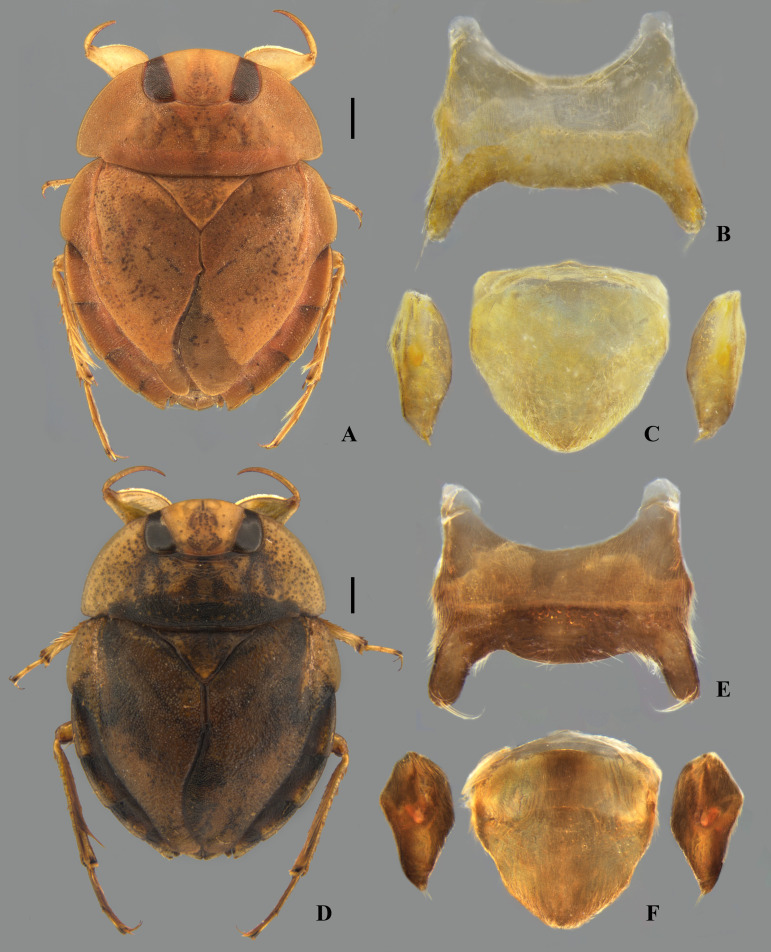
Limnocoris decarloi. **(A–C)** female collected at the type locality of *L. lanemeloi*. **(A)** Dorsal habitus, **(B)** abdominal tergum VII and **(C)** abdominal mediosternite VII (subgenital plate) + laterosternites VII, **(D–F)** female from population with greater body length from São Paulo, **(D)** Dorsal habitus, **(E)** abdominal tergum VII and **(F)** abdominal mediosternite VII (subgenital plate) + laterosternites VII.

**Fig 31:**
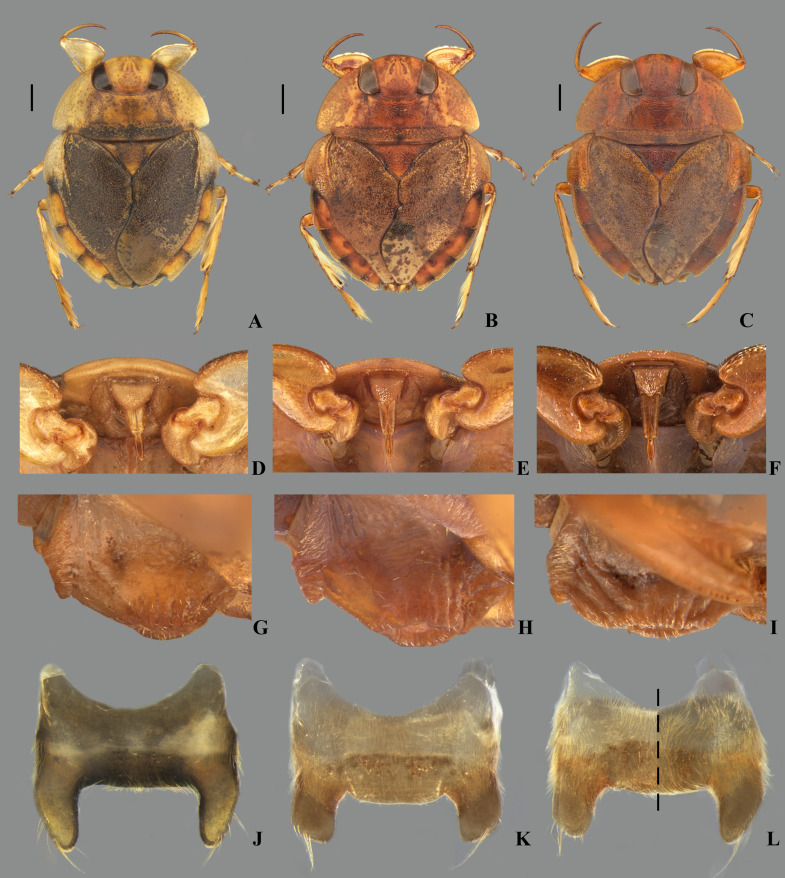
Morphotypes of *Limnocoris insignis.* Specimens from **(A, D, G, J)** Cachoeiras de Macacu, state of Rio de Janeiro, **(B, E, H, K)** Domingos Martins, state of Espírito Santo and **(C, F, I, L)** Baependi, state of Minas Gerais. **(A–C)** Dorsal habitus, **(D–F)** head in ventral view, **(G–I)** mesosternal carina in lateral view and **(J–L)** female abdominal tergum VII. **(L)** Brachypterous specimen on left of dotted line, macropterous specimen on right. Scale bar = 1 mm.

**Fig 32:**
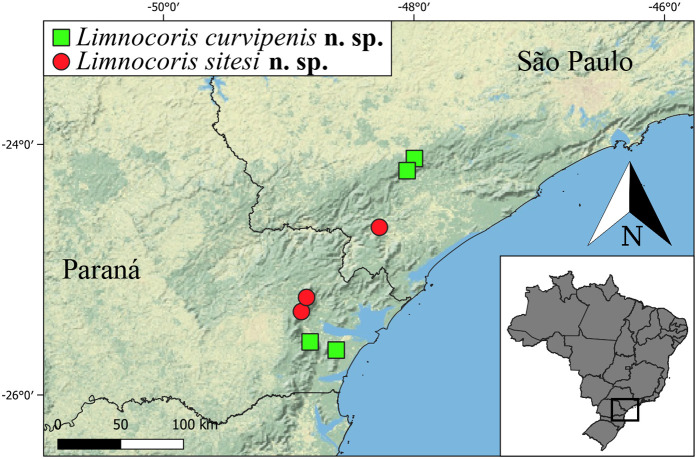
Geographic distribution of the two new species of *Limnocoris* described in this study. Basemap by ESRI®.

**Fig 33:**
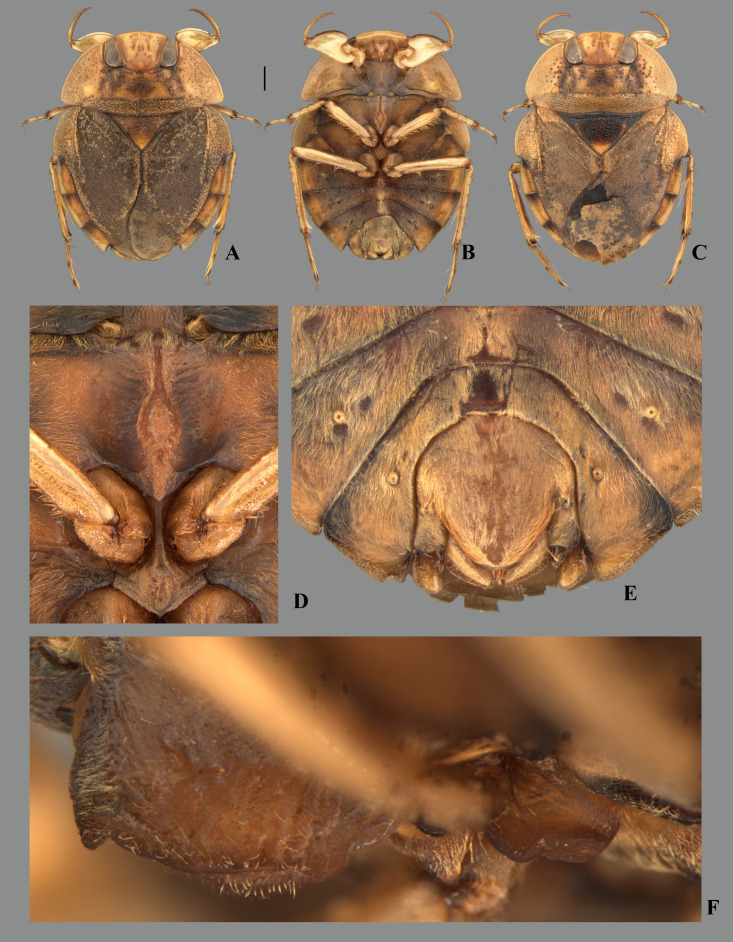
*Limnocoris curvipenis* Canejo, Rodrigues & Moreira n. sp. **(A)** Dorsal habitus and **(B)** ventral habitus of brachypterous male holotype, **(C)** dorsal habitus of macropterous female paratype, **(D)** ventral view of meso- and metasternal carinae, **(E)** ventral view of apex of female abdomen and **(F)** lateral view of meso- and metasternal carinae. Images not to scale. Scale bar = 1 mm, for A–C only.

**Fig 34:**
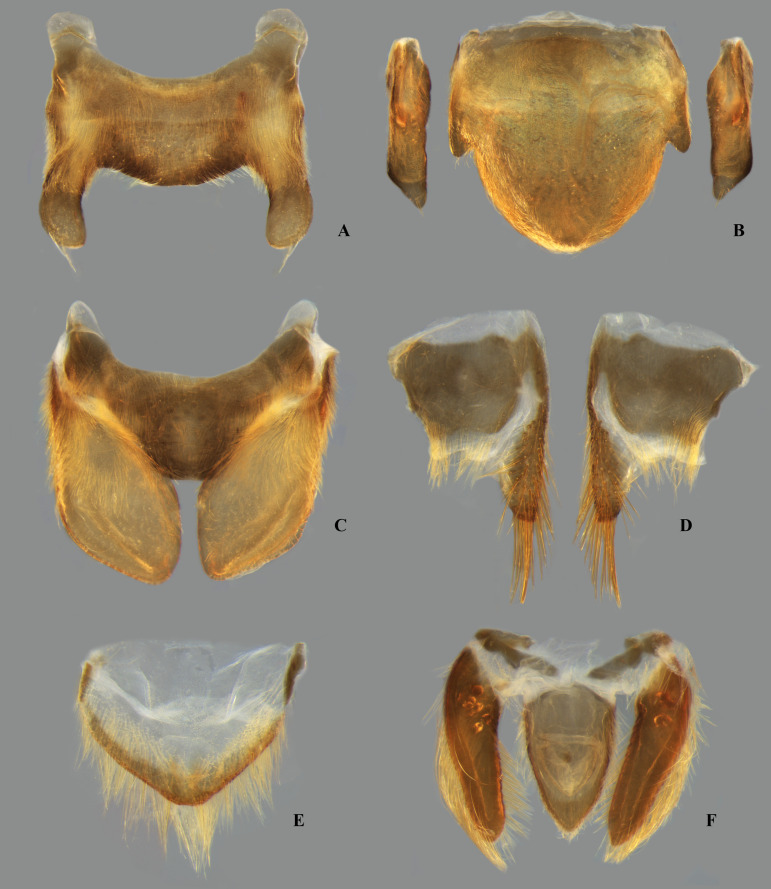
*Limnocoris curvipenis* Canejo, Rodrigues & Moreira n. sp. Female terminalia. **(A)** Abdominal tergum VII, **(B)** abominal mediosternite VII (subgenital plate) + laterosternites VII, **(C)** abdominal tergum VIII, **(D)** Valvifer I + Valvulae I, **(E)** Valvulae II and **(F)** Valvifer II + Valvulae III + abdominal mediotergite IX**.** Images not to scale.

**Fig 35:**
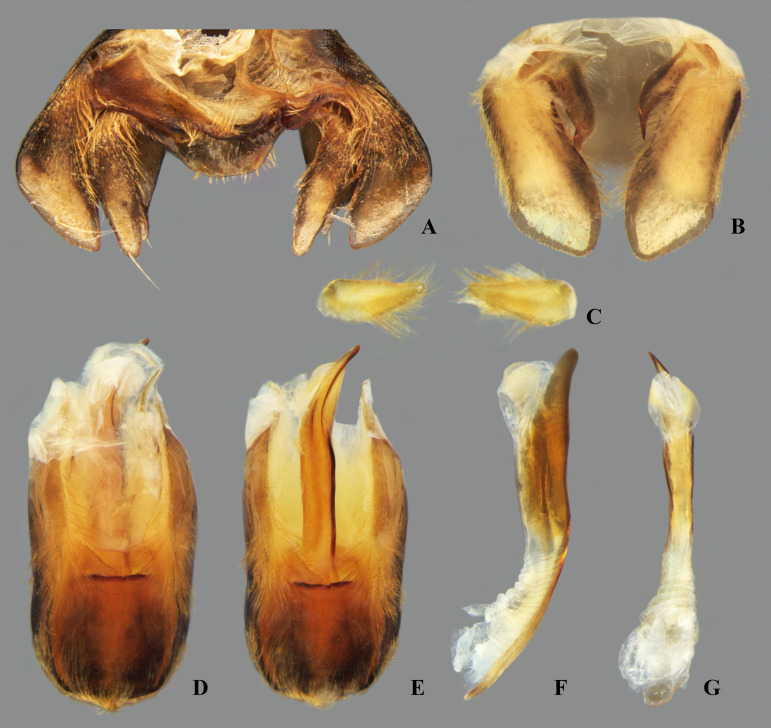
*Limnocoris curvipenis* Canejo, Rodrigues & Moreira n. sp. Male terminalia. **(A)** Abdominal terga VI + VII, **(B)** abdominal tergum VIII, **(C)** parameres, **(D)** genital capsule, **(E)** genital capsule (proctiger removed) showing phallosoma in dorsal view, **(F)** phallosoma in lateral view and **(G)** phallosoma in ventral view. Images not to scale.

**Fig 36:**
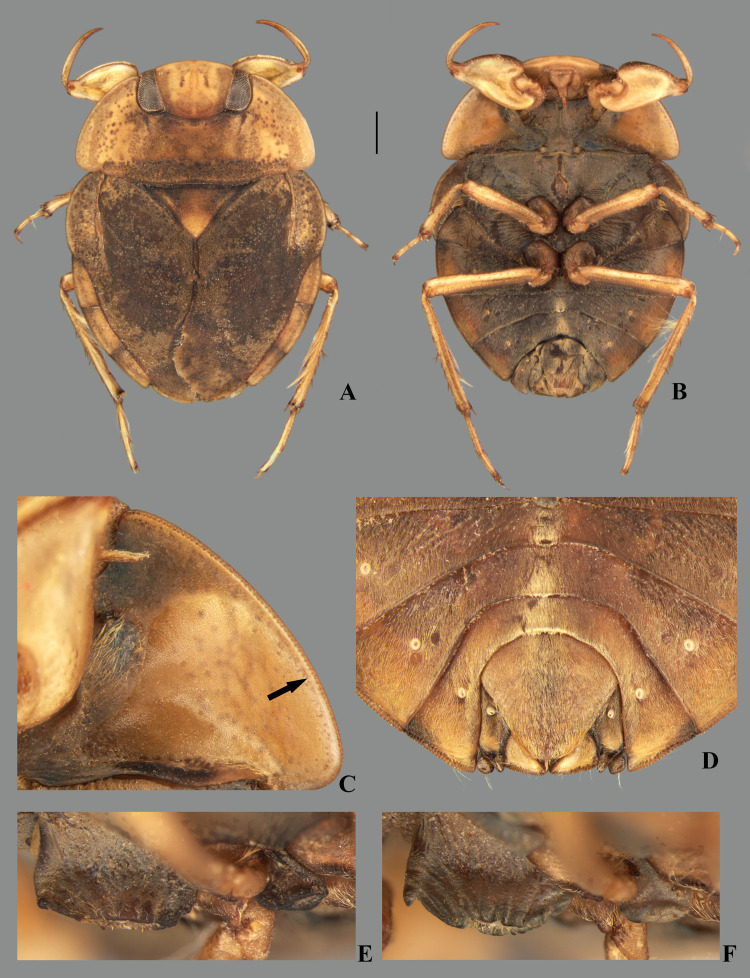
*Limnocoris sitesi* Canejo, Rodrigues & Moreira n. sp. **(A)** Dorsal habitus and **(B)** ventral habitus of brachypterous male holotype, **(C)** propleuron, arrow indicates apex of pruinose area, **(D)** ventral view of apex of female abdomen, **(E** and **F)** meso- and metasternal carinae in lateral view, showing variations in the median ridge and anterior projection of mesosternal carina. Scale bar = 1 mm, for A–B only.

***Limnocoris espinolai* Nieser & Lopez-Ruf, 2001**

(Figs 4G, 7G, 9G, 11G, 13G, 15G, 17G, 20A–D, 23G)

*Limnocoris espinolai* Nieser & Lopez-Ruf, 2001: 305, 307–308 (original description).

#### Material examined.

All specimens brachypterous. **BRAZIL, Minas Gerais**: [Morro do Pilar], Serra do Cipó, Córrego Indaiá, [–19.26, –43.52], ix.1999, G.J.C. Vianna col. (2♂, 2♀, CEIOC); Santuário do Caraça, L-1970, 20°5.834’S, 43°29.397’W, 26.xi.2016, R.W. Sites & H.D.D. Rodrigues col. (1♂, 1♀, CEIOC).

#### Female teminalia.

Tergum VII with average width 3x length at midline; lateral margins sinuous, convex in basal half; posterior margin sinuous; lateral lobes longer than wide, almost parallel (Fig 7G). Subgenital plate with uniform pilosity; lateral margins slightly diverging in basal half, converging in apical half to broadly rounded apex. Laterosternite VII convex at mesal margin, bearing single tuft of long setae posteriorly; lateral margin sinuous; apex acute (Fig 9G). Mediotergite VIII with lateral margin convex; posterior margin rounded. Laterotergite VIII with length twice greatest width; apical half of lateral margin serrated, bearing minute brush-like setae (Fig 11G). Valvifer I with mesal margin convex. Valvula I bearing robust, posteriorly directed setae, setae becoming longer towards apex; apical robust setae shorter than lateral margin (Fig 13G). Valvulae II slightly wider than long; lateral margin almost straight, bearing short setae; apex acuminate, bearing apical dark spot (Fig 15G). Valvula III flattened, sickle-shaped, with dense pubescence on surface; apical half slightly twisted mesally. Mediotergite IX ogival, longer than valvula III; lateral margin convex, apex rounded; length 1.4x greatest width; width at base less than twice width of valvula III (Fig 17G).

#### Male genitalia.

Pygophore with elongate setae sparsely distributed over most of surface, except for a tuft on median region of posterior margin; anterior margin slightly concave, posterior margin slightly convex. Phallosoma straight, with apex slightly bent dextrally in dorsal view (Figs 20A–D). Paramere with anterior margin concave in apical half; mesal angle narrowly rounded and curved anteriorly (Fig 23G).

#### Comments.

This species is most similar to *L. asper*, with which it was collected in sympatry in Serra do Cipó and Serra do Caraça, state of Minas Gerais. Both species have very similar structures in both the male and female terminalia, differing only slightly in the shape of the paramere and abdominal tergum VIII of both sexes. We still refrain from synonymizing these species because they were recovered in separate clades in a recent molecular phylogeny, although with poor support [6].

***Limnocoris fittkaui* De Carlo, 1967**

(Figs 4H, 7J, 9J, 11J, 13J, 15J, 17J, 20E–H, 23H)

*Limnocoris fittkaui* De Carlo, 1967: 195–196 (original description).

#### Material examined.

All specimens brachypterous. **BRAZIL, Amazonas**: Careiro da Várzea [sic, Careiro], Ramal do Purupuru, [–3.37, –59.85], 21.vi.2011, H.D.D. Rodrigues col. (1♀, CEIOC); Manaus, PDBFF, ZF3, Km 21, Faz.[enda] Esteio, Igarapé (R.[io] P.[reto] da Eva), mata contínua, barranco, raízes, [–2.4342, –59.9044], 12.ii.2001, J.L. Nessimian col. (1♂, CEIOC).

#### Female terminalia.

Tergum VII with average width 3x length at midline; lateral margins sinuous, convex approximately at mid length; posterior margin sinuous; lateral lobe much longer than wide, nearly straight, divergent, bearing dense row of setae in basal 2/3 of mesal and lateral margins (Fig 7J). Subgenital plate with tuft of long setae on apical 2/3 of lateral margin; lateral margin straight in basal 1/4, apical 3/4 converging to rounded apex. Laterosternite VII convex at mesal margin, bearing two tufts of long setae posteriorly; proximal tuft slightly shorter than distal tuft; lateral margins sinuous; apex acute (Fig 9J). Mediotergite VIII with lateral margin convex, ventrally folded; posterior margin broadly rounded. Laterotergite VIII length less than twice its width; apical half of lateral margin moderately serrated, converging posteriorly, bearing minute brush-like setae (Fig 11J). Valvifer I with mesal margin convex. Valvula I bearing robust, posteriorly directed setae, setae becoming slightly longer towards apex; apical robust setae shorter than lateral margin (Fig 13J). Valvulae II slightly longer than wide; lateral margin convex, with long setae; apex broadly rounded, sometimes with small concavity medially, apical dark spot present (Fig 15J). Valvula III flattened, sickle-shaped, with long, dense pubescence on surface; apical half slightly twisted mesally; apex narrowly rounded. Mediotergite IX ogival, slightly longer than valvula III; lateral margins parallel in basal 2/3, converging in apical 1/3 to rounded apex; uniform pilosity throughout surface; length 1.5x greatest width; width at base twice width of valvula III (Fig 17J).

#### Male genitalia.

Pygophore with elongate setae sparsely distributed over most of surface, except for a dense tuft on median region of posterior margin; anterior margin concave, posterior margin convex. Phallosoma straight, with apex slightly bent dextrally in dorsal view (Figs 20E–H). Paramere with anterior margin straight or slightly concave in apical half; mesal angle rounded (Fig 23H).

#### Comments.

This species has the female terminalia very similar to those of *L. menkei* and *L. minutus*. They share the posterior margin of abdominal tergum VII sinuous, with the lateral lobes divergent; and valvulae II longer than wide, with a conspicuous dark spot at the apex. However, *L. fittkaui* can be distinguished by valvula III with dense pubescence over its entire surface (Fig 17J), whereas in *L. menkei* and *L. minutus* it is restricted to the apical half (Figs 17M–N).

***Limnocoris illiesi* De Carlo, 1967**

(Figs 4I, 7K, 9K, 11K, 13K, 15K, 17K, 20I–J, 23I)

*Limnocoris illiesi* De Carlo, 1967: 192–193 (original description).

#### Material examined. BRAZIL, Minas Gerais.

Luz, Ribeirão Jorge Grande, [–19.6707, –45.6103], 13.iii.2010, H.D.D. Rodrigues & G.J.C.Vianna col. (1♀ brachypterous, CEIOC); Luz, Rib.[eirão] Jorjão [Jorge Grande], [–19.6707, –45.6103], 20.ii.2010, H.D.D. Rodrigues col. (2♂ brachypterous, CEIOC). **Rondônia**: Jaci-Paraná, #38, Rio Branco, Jaci-Paraná, 9°23’09.9“S, 64°22’50.7”W, 76 m, 26.vi.2014, N. Hamada & J.M.C. Nascimento (1♂ brachypterous, CEIOC). **Roraima**: [Amajari], 1636, Rio Uraricoera, Aldeia Parimiu, [3.24, –62.78], 24.v.1987, V. Py-Daniel & U. Barbosa col. (1♀ macropterous, CEIOC).

#### Female terminalia.

Tergum VII with average width 2.5x length at midline; lateral margin straight; posterior margin almost straight; lateral lobe longer than wide, broadly rounded posteriorly, slightly divergent (Fig 7K). Subgenital plate with uniform pilosity; lateral margin parallel in basal 1/3, concave at mid length, converging to broadly rounded posterior margin. Laterosternite VII convex at mesal margin, bearing a single tuft of long setae posteriorly; lateral margin straight; apex rounded (Fig 9K). Mediotergite VIII with lateral margin straight; posterior margin narrowly rounded. Laterotergite VIII with length about twice greatest width; apical half of lateral margin weakly serrated, converging posteriorly, bearing large brush-like setae (Fig 11K). Valvifer I with mesal margin nearly straight; pilosity uniform. Valvula I bearing robust, posteriorly directed setae; apical robust setae shorter than lateral margin (Fig 13K). Valvulae II as wide as long; lateral margin convex, with long setae; apex slightly concave at midline, without apical dark spot (Fig 15K). Valvula III thickened, sickle-shaped, with long, dense pubescence near apex; apical 2/3 twisted mesally; broadly rounded. Mediotergite IX ogival, shorter than valvula III; lateral margins convex, converging to broadly rounded apex; uniform pilosity throughout surface; length 1.5x greatest width; width at base less than twice width of valvula III (Fig 17K).

#### Male genitalia.

Pygophore with elongate setae sparsely distributed over most of its surface; anterior margin concave, posterior margin nearly straight. Phallosoma straight (Figs 20M–P). Paramere with anterior margin straight in apical half; mesal angle rounded (Fig 24J).

#### Comments.

The female terminalia of this species are similar to those of *L. pusillus* Montandon, 1897. However, *L. illesi* differs from it by the following characteristics: the posterior margin of the subgenital plate is broadly rounded, the lateral margin of laterosternite VII is nearly straight (Fig 9K), the posterior margin of mediotergite VIII is narrowly rounded, and the basal half of the lateral margin of laterotergite VIII is straight (Fig 11K) In *L. pusillus*, however, the posterior margin of the subgenital plate converges to a narrowly rounded apex, the lateral margin of laterosternite VII is concave (Fig 10C), the posterior margin of mediotergite VIII is broadly rounded, and the basal half of the lateral margin of laterotergite VIII is concave (Fig 12C).

***Limnocoris insignis* Stål, 1860**

(Figs 7J, 9J, 11J, 13J, 15J, 17J, 20M–P, 23J, 31)

*Limnocoris insignis* Stål, 1860: 83 (original description).

*Limnocoris uhleri* Montandon, 1910: 440–442 (original description) (synonymized by Nieser & Lopez-Ruf, 2001: 279).

#### Material examined. BRAZIL, Espírito Santo.

Domingos Martins, Alto Galo, 20°17’15.0“S, 40°38’30.0”W, H.D.D. Rodrigues col. (5♂, 5♀ brachypterous, CEIOC); Santa Teresa, Nova Lombardia, Capitel de Santo Antonio, Córrego Grande, Pinguela, Cascalho, 19°52’16.0”S, 40°31’43.1”W, 18.ii.2008, (3♂, 2♀ brachypterous, CEIOC). **Minas Gerais**: Baependi, Cachoeira de Itaúna, ca. 15 km SE. of Baependi, 22°02’32.2”S, 44°49’12.7”W, 923 m, 02.xii.2016, H.D.D. Rodrigues col. (4♂, 4♀ brachypterous, 3♂, 3♀ macropterous, CEIOC); Brumadinho, Cachoeira da Ostra, 20°5.710’S, 44°0.957’W, 989 m, 04.xii.2016, H.D.D. Rodrigues col. (1♂ brachypterous, CEIOC); Luminárias, Ribeirão da Ponte, 2 km E. of Luminárias, 21°31’00.5”S, 44°52’16.1”W, 966 m, 03.xii.2016, (2♂, 3♀ brachypterous, CEIOC); Piranguçu, Cachoeira São Bernardo, 22°34’41.15S, 45°29’13.9”W, 01.x.2017, F.F.F. Moreira, I.R.S. Cordeiro, F.S. Motta & T.S. Martins col. (5♂, 1♀ brachypterous, 1♀ macropterous, CEIOC). **Paraná**: Paranaguá, P17, Riacho, 25°35’17.6”S, 48°38’04.6”W, 21 m, 30.x.2023, R.P.R. Canejo, J.M.S. Rodrigues, M.S.L. Alexandre & L.D. Pereira col. (5♂, 8♀ brachypterous, 6♂, 3♀ macropterous, CEIOC); Paranaguá, PR15, Parque Nacional Saint Hilaire/Lange, Cachoeira Quintilha, 25°38’28.9”S, 48°37’12.5”W, 125 m, 30.x.2023, R.P.R. Canejo, J.M.S. Rodrigues, M.S.L. Alexandre & L.D. Pereira col. (1♂ brachypterous, 1♂ macropterous, CEIOC); Morretes, Bairro América de Cima, [–25.48, –48.87], 16.xi.2019, B.R. Araujo col. (1♂, 3♀ brachypterous, CEIOC). **Rio de Janeiro**: Cachoeiras de Macacu, PE [Parque Estadual] dos Três Picos, Boca do Mato, Rio Macacu, Poço das Borboletas, PETP12, 22°24’44.3”S, 42°36’41.0”W, 274 m, 24.v.2024, C.C.D. Correia, I.C. Gonçalves, M.D. Duarte, M.V.O. de Almeida, N.O. Paiva & R.P.R. Canejo col. (3♂, 3♀ brachypterous, 12♂, 3♀ macropterous, CEIOC); same data, except: Próximo ao PE dos Três Picos, Castália, Rio Castália, Barracão das Bananas, PETP09, 22°25’07.5”S, 42°39’26.2”W, 338 m, 22.v.2024 (1♀ brachypterous, 14♂, 9♀ macropterous, CEIOC); same data, except: Boca do Mato, Rio Macacu, PETP03, 22°24’49.6”S, 42°36’50.8”W, 298 m, 22.v.2024 (1♀, 1♀ brachypterous, CEIOC); same data, except: Boca do Mato, Rio Macacu, Ponte Velha, PETP06, 22°24’54.5”S, 42°36’56.1”W, 277 m, 21.v.2024 (7♂, 7♀ brachypterous, 12♂, 5♀ macropterous, CEIOC); Nova Friburgo, APA [Área de Proteção Ambiental de] Macaé de Cima, Mury, Rio Macaé, APA Macaé 01, 22°23’30.2”S, 42°29’06.7”W, 927 m, 23.v.2024, C.C.D. Correia, I.C. Gonçalves, M.D. Duarte, M.V.O. de Almeida, N.O. Paiva & R.P.R. Canejo col. (11♂, 10♀ brachypterous, CEIOC); same data, except: Lumiar, Encontro dos Rios Bonito e Macaé, APA Macaé 03, 22°23’16.8”S, 42°18’32.2”W, 560 m, 23.v.2024 (10♂, 10♀ brachypterous, CEIOC). **São Paulo**: Eldorado, Cachoeira do Sapatú, CDD11, 24°39’53.2”S, 48°19’46.9”W, 145 m, 04.VII.2024, E.A. Joaquim, L.L. Dumas, K.O. Souza & R.P.R. Canejo col. (9♂ brachypterous, CEIOC); Eldorado, Rio da Madre-Ribeirão Martins, CDD06, 24°39’40.8”S, 48°16’32.0”W, 73 m, 03.VII.2024, E.A. Joaquim, L.L. Dumas, K.O. Souza & R.P.R. Canejo col. (17♂, 5♀ brachypterous, CEIOC); Eldorado, Rio Batatal, Trilha para a cachoeira do Machadinho, CDD11, 24°39’45.5”S, 48°19’41.7”W, 100 m, 04.VII.2024, E.A. Joaquim, L.L. Dumas, K.O. Souza & R.P.R. Canejo col. (8♂,9♀ brachypterous, CEIOC); Eldorado, Rio Ribeira do Iguape, CDD16, 24°35’42.2”S, 48°23’35.5”W, 48 m, 05–06.VII.2024, E.A. Joaquim, L.L. Dumas & K.O. Souza, R.P.R. Canejo col. (5♂ brachypterous, CEIOC); Eldorado, Rio da Madre, trecho com mais correnteza, CDD08, 24°39’41.9”S, 48°16’35.8”W, 73 m, 03–04.VII.2024, E.A. Joaquim, L.L. Dumas, K.O. Souza & R.P.R. Canejo col. (1♀ brachypterous, CEIOC); Eldorado, Ribeirão das Pedras, CDD10, 24°41’13.6”S, 48°18’40.6”W, 163 m, 04.VII.2024, E.A. Joaquim, L.L. Dumas, K.O. Souza & R.P.R. Canejo col. (11♂,1♀ brachypterous, CEIOC); [Sete Barras], Paranapiacaba, Ponto 3, Bananal, [–24.2919, –48.1081], vii.2013 (2♂, 4♀ brachypterous, CEIOC); same data, except x.2013 (1♂, 2♀ brachypterous, CEIOC); same data, except xi.2013 (11♂, 18♀ brachypterous, CEIOC); [Sete Barras], Paranapiacaba, Ponto 6, Pós-Bananal, [–24.30, –48.10], vii.2013 (2♂, 1♀ brachypterous, CEIOC); same data, except viii.2013 (1♀ brachypterous, CEIOC); same data, except ix.2013 (1♂, 1♀ brachypterous, 1♂ macropterous, CEIOC); same data, except xi.2013 (1♂, 1♀ brachypterous, CEIOC); [Sete Barras], Paranapiacaba, Ponto 1, Área Preservada, [–24.2919, –48.1081], vii.2013 (3♂, 7♀ brachypterous, CEIOC); same data, except viii.2013 (4♂, 6♀ brachypterous, 2♂, 1♀ macropterous, CEIOC); same data, except ix.2013 (1♂, 2♀ brachypterous, CEIOC); [Sete Barras], Paranapiacaba, Ponto 2, Transição, [–24.2731, –48.1075], viii.2013 (2♂ brachypterous, CEIOC); same data, except Intermediário, xi.2013 (1♀ brachypterous, CEIOC); same data, except vii.2013 (3♀ brachypterous, 1♂ macropterous, CEIOC); São Miguel Arcanjo, SP3, Reserva Particular do Patrimônio Natural Parque Taquaral da Mata Atlântica, Rio Taquaral, 24°04’24.5”S, 47°59’51.2”W, 707 m, 16.xi.2023, L.L. Dumas, J.M.S. Rodrigues & R.P.R. Canejo col. (18♂, 6♀ brachypterous, CEIOC); São Miguel Arcanjo, SP5, Parque Estadual Carlos Botelho, Rio Taquaral, bica, 24°03’43.4”S, 47°59’58.4”W, 721 m, 16.xi.2023, L.L. Dumas, J.M.S. Rodrigues & R.P.R. Canejo col. (2♂, 2♀ brachypterous, CEIOC); Capão Bonito, SP6, Parque Estadual Carlos Botelho, rio na trilha para a Cachoeira do Muriqui, 24°06’32.9”S, 47°59’13.3”W, 756 m, 17.xi.2023, L.L. Dumas, J.M.S. Rodrigues & R.P.R. Canejo col. (4♂, 3♀ brachypterous, CEIOC); Capão Bonito, SP7, Parque Estadual Carlos Botelho, Cachoeira do Muriqui, 24°06’44.8”S, 47°59’44.0”W, 802 m, 17.xi.2023, L.L. Dumas, J.M.S. Rodrigues & R.P.R. Canejo col. (1♂, 1♀ brachypterous, CEIOC); Capão Bonito, SP9, Parque Estadual Carlos Botelho, Rio Taquaral, prainha, 24°03’52.4”S, 47°59’59.0”W, 710 m, 17.xi.2023, L.L. Dumas, J.M.S. Rodrigues & R.P.R. Canejo col. (48♂, 22♀ brachypterous, CEIOC); Sete Barras, SP10, Parque Estadual Carlos Botelho, açude, 24°11’39.2”S, 47°55’13.7”W, 98 m, 18.xi.2023, L.L. Dumas, J.M.S. Rodrigues & R.P.R. Canejo col. (1♀ macropterous, CEIOC); Sete Barras, SP11, Parque Estadual Carlos Botelho, Trilha da Figueira, rio e riachos, 24°11’45.6”S, 47°55’32.7”W, 76 m, 18.xi.2023, L.L. Dumas, J.M.S. Rodrigues & R.P.R. Canejo col. (2♂, 1♀ brachypterous, CEIOC); Sete Barras, SP14, Rio Preto, 24°11’35.0”S, 47°53’26.0”W, 30 m, 19.xi.2023, L.L. Dumas, J.M.S. Rodrigues & R.P.R. Canejo col. (1♀ brachypterous, 4♂, 9♀ macropterous, CEIOC); Sete Barras, SP42, Parque Estadual Carlos Botelho, Cachoeira do Quilombo, 24°12’34.6”S, 48°03’14.8”W, 147 m, 07.xii.2023, L.L. Dumas, J.M.S. Rodrigues, L. Nery & L. Hoehne col. (17♂, 5♀ brachypterous, 1♂, 2♀ macropterous, CEIOC); Ribeirão Grande, SP39, Rio das Almas, cachoeira, 24°09’26.6”S, 48°21’07.6”W, 699 m, 05.xii.2023, L.L. Dumas, J.M.S. Rodrigues, L. Nery & L. Hoehne col. (1♂ brachypterous, 1♀ macropterous, CEIOC). **Santa Catarina**: PARNA [Parque Nacional da] Serra do Itajaí, Parque das Nascentes, Rio Garcia, [–27.06, –49.09], iv.2017, C.B. Floriano col. (6♂, 7♀ brachypterous, CEIOC); Águas Mornas [Santo Amaro da Imperatriz/ Águas Mornas], Rio Braço da Forquilha, [–27.6927, –48.8334], x.2016, C.F.B. Floriano, (7♀ brachypterous, CEIOC).

#### Female terminalia.

Tergum VII with average width 2.5x length at midline; lateral margins sinuous, concave in basal half; posterior margin straight or convex; lateral lobes longer than wide, rounded posteriorly, posterolateral margin bearing longer sparse setae; brush-like setae sometimes present (Fig 7J). Subgenital plate with row of golden setae on posterolateral margin; lateral margins parallel in basal 1/3; apical 2/3 converging to broadly rounded, sometimes truncate, apex. Laterosternite VII convex at mesal margin, bearing single tuft of long setae posteriorly; lateral margin slightly sinuous; apex acute (Fig 9J). Mediotergite VIII with lateral margin concave; posterior margin broadly rounded. Laterotergite VIII length more than twice width; apical half of lateral margin weakly serrated, converging posteriorly, bearing minute brush-like setae (Fig 11J). Valvifer I with mesal margin convex. Valvula I bearing robust, posteriorly directed setae, setae becoming longer towards apex; apical robust setae longer than lateral margin (Fig 13J). Valvulae II slightly wider than long; lateral margin almost straight, bearing short setae; apex acuminate, without apical dark spot (Fig 15J). Valvula III thickened, baton-shaped, with long, dense pubescence basally; apical half slightly twisted; apex truncate; posterior region of ventral surface with shallow diagonal indentation. Mediotergite IX ogival, shorter than valvula III; lateral margin convex, converging to narrowly rounded apex; uniform pilosity throughout its surface; length 1.5x greatest width; width at base less than twice width of valvula III (Fig 17J).

#### Male genitalia.

Pygophore with elongate setae sparsely distributed over most of surface, except for a dense tuft on median region of posterior margin; anterior margin straight, posterior margin convex. Phallosoma straight (Figs 20M–P). Paramere with anterior margin straight or shallowly concave in apical half; mesal angle narrowly rounded (Fig 24J).

#### Comments.

Specimens from the municipality of Baependi, state of Minas Gerais (Fig 31C), have consistent differences in relation to most other populations of *L. inisgnis* that we examined. Individuals from Baependi are larger on average (8.8 mm). They also display dimorphism in the length of labial article III between brachypterous and macropterous specimens: it is about twice the length of article IV in the brachypterous form (Fig 31F), but about 1.5x or less the length of article IV in the macropterous form. This was not observed in most other populations of *L. insignis* (Fig 31D). The median ridge of the mesosternal carina is convex in specimens from Baependi (Fig 31I), whereas it is straight in all other populations (Figs 31G–H). The female terminalia also have dimorphism in the shape of the lateral lobes of abdominal tergum VII related to wing development: in brachypterous specimens the lateral lobe is quadrate (Fig 31L, left of dotted line), whereas in macropterous specimens it is more elongate and convergent on the lateral margin (Fig 31L, right of dotted line), which is the shape found in other populations (as in Fig 31J). Although these differences were consistent in Baependi, specimens from Domingos Martins, state of Espírito Santo, have characteristics of both morphotypes (Fig 31B). They have about the same average length as the most common morphotype (8.0 mm), as well as the median ridge of the mesosternal carina straight (Fig 31H). However, they also have labial article III twice as long as article IV (Fig 31D), and the lateral lobes of tergum VII quadrate (Fig 31K). We did not examine macropterous specimens to confirm if this population presents the same dimorphism observed in the material from Baependi. Furthermore, no consistent differences in male terminalia were found among morphotypes. Since the macropterous forms of these populations are almost indistinguishable, and the specimens from Domingos Martins appear to be intermediate, we identified all morphotypes as *L. insignis*. Future studies may confirm or refute our hypothesis.

The female of *L. insignis* shares the following feature with *L. admontandoni*, *L. rotundatus* De Carlo, 1951 and *L. sattleri* De Carlo, 1966: the posterior region of valvula III is indented ventrally (Figs 17J, 18C–D, 26F). It can be distinguished from *L admontandoni* by the head not distinctly projected anteriorly, even in specimens with an elongated labial article III (Figs 31A–F). Also, in *L. admontandoni*, the valvula III is strongly indented and folded over the posterior margin of the subgenital plate (Figs 25E, 26F), whereas in *L. insignis* the indentation is not as deep (Fig 17J). *Limnocoris insignis* can be distinguished from *L. rotundatus* by the laterosternite VII not strongly projected posterolaterally (Fig 9J), which is found in the latter (Fig 10C). Finally, it can be distinguished from *L. sattleri* by the lateral margin of the subgenital plate convex (Fig 9J) (nearly straight in *L. sattleri*) (Fig 10D), and valvula III with a deeper indentention (Fig 17J) (shallow in *L. sattleri*) (Fig 18D).

***Limnocoris machrisi* Nieser & Lopez-Ruf, 2001**

(Figs 5A, 7K, 9K, 11K, 13K, 15K, 17K, 20Q–T, 23K)

*Limnocoris machrisi* Nieser & Lopez-Ruf, 2001: 313–314 (original description).

#### Material examined.

All specimens macropterous. **BRAZIL, Mato Grosso**: Chap.[ada] dos Guimarães, C.[órrego] Independência, [–15.42, –55.84], 09.xi.2013, C. Floriano col. (1♀, CEIOC); 5149, Cuiabá [sic, Chapada dos Guimarães], P.N. [Parque Nacional da] Chapada dos Guimarães, Córrego Independência, Degraus/prainha, 15°24’58.70“S, 55°50’33.70”W, 578 m, 20.VII.2013, A.L.H. Oliveira, B.H.L. Sampaio, B. Clarkson & N. Ferreira Jr. col. (1♂, 3♀, DZRJ); Chap. dos Guimarães, Cach.[oeira] do Pulo, [–15.416, –55.842], 529 m, 19.vii.2013, (1♂, CEIOC).

#### Female terminalia.

Tergum VII with average width 2.5x the length at midline; lateral margins sinuous, convex in basal 1/3; lateral lobes much longer than wide, divergent at base, convergent at apex, bearing row of dense setae in basal 2/3 of median and lateral margins; posterior margin sinuous, poorly convex at mid-length (Fig 7K). Subgenital plate with tufts of long setae on apical half of lateral margin; lateral margins parallel in basal half; apical half slightly concave, converging to broadly rounded apex. Laterosternite VII distinctly convex at mesal margin, bearing two tufts of long setae posteriorly; proximal tuft as long as distal tuft; lateral margins sinuous; apex sharply acuminate, medially directed (Fig 9K). Mediotergite VIII with lateral margin convex, ventrally reflected; posterior margin broadly rounded to truncate. Laterotergite VIII length less than twice its width; apical half of lateral margin moderately serrated, converging posteriorly, and bearing minute brush-like setae (Fig 11K). Valvifer I with mesal margin convex. Valvula I bearing robust, posteriorly directed setae, becoming longer towards apex; apical robust setae shorter than lateral margin, including cluster of short, curved setae (Fig 13K). Valvulae II as long as wide; lateral margin convex, with long setae becoming longer towards apex; apex narrowly rounded or notched medially; apical dark spot sometimes present (Fig 15K). Valvula III flattened, sickle-shaped, with long, dense pubescence on apical half, which is medially twisted; apex rounded. Mediotergite IX ogival, longer than valvula III; lateral margins parallel in basal 2/3, converging to narrowly rounded apex in apical 1/3; length 1.7x the greatest width; width at base twice width of valvula III (Fig 17K).

#### Male genitalia.

Pygophore with elongate setae sparsely distributed over most of surface, except for a dense tuft on median region of posterior margin; anterior margin concave, posterior margin convex. Phallosoma straight in dorsal view, with apex slightly bent dextrally (Figs 20U–X). Paramere with anterior margin concave in apical half; mesal angle narrowly rounded, slightly curved anteriorly (Fig 23K).

#### Comments.

The female terminalia of this species are nearly identical to those of *L. volxemi*, which has a very similar general morphology, except for the body size and shape of the mesosternal carina. They also share an exclusive feature, which is the apex of valvula I with a cluster of short, stout, curved setae on the mesal margin (Figs 13K, 14H). No consistent differences were found on the male or female genitalia between these two species.

***Limnocoris melloleitaoi* De Carlo, 1951**

(Figs 5B, 7L, 9L, 11L, 13L, 15L, 17L, 20U–X, 23L)

*Limnocoris melloleitaoi* De Carlo, 1951: 46–47 (original description).

#### Material examined. BRAZIL, Paraná.

Morretes, PR12, Cachoeira do Jajá, 25°34’34.8“S, 48°49’43.6”W, 244 m, 30.x.2023, R.P.R. Canejo, J.M.S. Rodrigues, M.S.L. Alexandre & L.D. Pereira col. (11♂, 9♀ brachypterous, 2♂ macropterous, CEIOC); Campina Grande do Sul, Parque Estadual Pico do Paraná, Cachoeira do Arco-Íris, antes da queda, 25°13’16.8”S, 48°51’26.0”W, 960 m, 31.x.2023, R.P.R. Canejo, J.M.S. Rodrigues, M.S.L. Alexandre & L.D. Pereira col. (6♂, 13♀ brachypterous, 1♂ macropterous, CEIOC). **São Paulo**: Capão Bonito, SP7, Parque Estadual Carlos Botelho, Cachoeira do Muriqui, 24°06’44.8”S, 47°59’44.0”W, 802 m, L.L. Dumas, J.M.S. Rodrigues & R.P.R. Canejo col. (3♂, 4♀ brachypterous, CEIOC); Sete Barras, SP12, Parque Estadual Carlos Botelho, Ponte Aristides Manoel, Água da Vaca, cachoeira, 24°09’53.1”S, 47°58’54.6”W, 719 m, 18.xi.2023, L.L. Dumas, J.M.S. Rodrigues & R.P.R. Canejo col. (12♂, 12♀ brachypterous, 1♂ macropterous, CEIOC); Sete Barras, SP15, Parque Estadual Carlos Botelho, riacho, 24°07’39.3”S, 47°59’33.4”W, 773 m, 19.xi.2023, L.L. Dumas, J.M.S. Rodrigues & R.P.R. Canejo col. (2♀ brachypterous, 1♂ macropterous, CEIOC); Santo André, Paranapiacaba, Serra do Mar, trilha p/[ara] cach.[oeira] da Fumaça, 23°47’15.6”S, 46°21’43.5”W, 746 m, 24.i.2015, H.D.D. Rodrigues col. (1♂brachypterous, CEIOC); Santo André, Paranapiacaba, Serra do Mar, Rio Vermelho, 23°47’17.7”S, 46°22’36.0”W,761 m, 24.i.2015, H.D.D. Rodrigues col. (1♀ brachypterous, CEIOC).

#### Female terminalia.

Tergum VII with average width 3x length at midline; lateral margin sinuous, convex in basal half; posterior margin sinuous; lateral lobe longer than wide, divergent (Fig 7L). Subgenital plate with tufts of long setae on apical half of lateral margin; lateral margins convex in basal half, straight and converging to a rounded apex in apical half. Laterosternite VII convex and angulated at mesal margin, bearing two tufts of long setae posteriorly; proximal tuft longer than distal tuft; lateral margins sinuous; apex sharply acuminate (Fig 9L). Mediotergite VIII with lateral margin convex, ventrally folded; posterior margin broadly rounded. Laterotergite VIII length less than twice its width; apical half of lateral margin weakly serrated, bearing minute brush-like setae, converging posteriorly (Fig 11L). Valvifer I with mesal margin convex. Valvula I bearing robust, posteriorly directed setae, setae becoming longer towards apex; apical robust setae shorter than lateral margin (Fig 13L). Valvulae II slightly longer than wide; lateral margin convex, with long setae; apex broadly rounded, bearing apical dark spot (Fig 15L). Valvula III flattened, sickle-shaped, with long, dense pubescence on apical half, which is medially twisted; apex rounded. Mediotergite IX with lateral margin convex in basal 3/4; apical 1/4 concave, converging to narrowly rounded apex; length equal to valvula III; pilosity on surface becoming denser distally; length 1.6x greatest width; width at base less than twice width of valvula III (Fig 17L).

#### Male genitalia.

Pygophore with elongate setae sparsely distributed over most of surface, except for dense tuft on median region of posterior margin; anterior margin concave, posterior margin straight to slightly convex. Phallosoma straight in dorsal view, with apex slightly bent dextrally (Figs 20U–X). Paramere with anterior margin concave in apical half; mesal angle narrowly rounded, slightly curved anteriorly in some specimens (Fig 24L).

#### Comments.

Among the species examined in this study, *L. melloleitaoi* has the female terminalia morphologically similar to those of *L. amazonensis*, *L. burmeisteri*, *L. fittkaui*, *L. machrisi*, *L. menkei*, *L. minutus* and *L. volxemi*. These species share the posterior margin of tergum VII sinuous, the lateral margins of the subgenital plate with long curved setae, the lateral margins of mediotergite VIII ventrally reflected (Fig 1H); and mediotergite IX longer than valvula III. *Limnocoris melloleitaoi*, however, can be distinguished from congeners by the rounded apex of the lateral lobe of abdominal tergum VII (Fig 7L), which is angulated (Figs 8K, L) or sharply acuminate (Figs 7B, E, H, K, M, N, 8H) in the other species. Also, mediotergite IX is convex in the basal 3/4 and concave in the apical 1/4 of the lateral margin (Fig 17L), whereas in the other species it has the lateral margin straight (Figs 17B, E, H, K, M, N). No consistent differences were found in the male genitalia to distinguish *L. melloleitaoi* from these similar species.

***Limnocoris menkei* La Rivers, 1962**

(Figs 5C, 7M, 9M, 11M, 13M, 15M, 17M, 21A–D, 24A)

*Limnocoris menkei* La Rivers, 1962: 195–196 (original description).

*Limnocoris birabeni* De Carlo, 1967: 193–194 (original description) (synonymized by Rodrigues & Sites, 2023: 65).

*Limnocoris bruchi* De Carlo, 1967: 194–195 (original description) (synonymized by Rodrigues & Sites, 2023: 63).

#### Material examined. BRAZIL, Pará.

Pará, Santarém, Ig.[arapé] Ponte do Juá, gramíneas, [–2.4446, –54.7892], 10.x.2020, (1♂, 1♀ brachypterous, LETIA); same data, except 25.i.2023, S.E. Santos col. (10♂, 10♀ brachypterous, LETIA); Itaituba, Miritituba, [–4.2961, –55.9711], 10.ii.2022, M. Vieira col. (6♂, 3♀ macropterous, LETIA).

#### Female terminalia.

Tergum VII with average width 3.3x length at midline; lateral margin sinuous, basal half convex; posterior margin sinuous, bearing row of setae extending to halfway of mesal margins of lateral lobes; lateral lobe distinctly longer than wide, diverging at base, almost parallel distally, bearing row of setae on lateral margin (Fig 7M). Subgenital plate with tufts of long setae on apical 2/3 of lateral margins; lateral margins convex in basal 1/3; apical 2/3 convex, converging to broadly rounded, almost straight posterior margin. Laterosternite VII convex at mesal margin, bearing two tufts of long setae posteriorly; proximal tuft as long as distal tuft; lateral margins sinuous; apex narrow, acute (Fig 9M). Mediotergite VIII with lateral margin convex, ventrally folded; posterior margin broadly rounded to truncate. Laterotergite VIII with length less than twice greatest width; posterior half of lateral margin serrated, bearing minute brush-like setae, converging posteriorly (Fig 11M). Valvifer I with mesal margin convex. Valvula I bearing robust, posteriorly directed setae, setae becoming longer towards apex; apical robust setae shorter than lateral margin (Fig 13M). Valvulae II slightly longer than wide; lateral margin slightly concave, with short setae; apex broadly rounded; with apical dark spot (Fig 15M). Valvula III flattened, sickle-shaped, with long dense pubescence on apical half, which is medially twisted; apex narrowly rounded. Mediotergite IX ogival, longer than valvula III; lateral margins parallel in basal 1/3; apical 2/3 converging to narrowly rounded apex; length twice greatest width; width at base twice width of valvula III (Fig 17M).

#### Male genitalia.

Pygophore with elongate setae sparsely distributed over most of surface, except for a dense tuft on median region of posterior margin; anterior margin concave, posterior margin convex; Phallosoma straight (Figs 21A–D). Paramere with anterior margin concave in apical half; mesal angle narrowly rounded (Fig 24A).

#### Comments.

The female terminalia of this species have very similar structures to those of *L. burmeisteri, L. fittkaui* and *L. minutus*. However, the mesal margins of the lateral lobes of tergum VII are nearly parallel posteriorly (Fig 7M), whereas they are converging in *L. burmeisteri* (Fig 7E) and divergent in *L. fittkaui* (Fig 7H) and *L. minutus* (Fig 7N). Also, the subgenital plate is broadly rounded (Fig 9M) or almost straight in some specimens of *L. menkei*, whereas it is rounded or narrowly rounded in the other species. No consistent differences were found in the male genitalia to distinguish *L. menkei* from these similar species.

***Limnocoris minutus* De Carlo, 1951**

(Figs 5D, 7N, 9N, 11N, 13N, 15N, 17N, 21E–H, 24B)

*Limnocoris minutus* De Carlo, 1951: 49–50 (original description).

#### Material examined. BRAZIL, Maranhão.

Caxias, Água Sumida, P5, 00080, [−4.9731, −43.0729], 11.ix.2021, (1♂ brachypterous, LEAq); Caxias, Estiva, P04, 00089, [−4.9281, −43.2444], 02.vii.2021, (4♂, 1♀ brachypterous, LEAq); same data, except P05, 00081 (1♀ brachypterous). Timon, Estrada Portal da Amazônia, 05°03’03.3“S, 43°01’52.2”W, 09.vi.2011, N. Hamada, P.V. Cruz & R.B. Querino col. (1♂ brachypterous, LEAq). **Mato Grosso**: Querência, Fazenda Tanguro, 12°54’S, 52°22’W, 2007, E. Wanzeler col. (1♀ brachypterous, CEIOC). **Mato Grosso do Sul**: BB-P2-PE, [−23.8165, −54.4453], vi.2008, C.F.B. Floriano col. (17♂, 13♀ brachypterous, CEIOC). **Rio Grande do Norte**: Pedro Velho, Rio Piquiri, Balneario, 6°25’04.2”S, 35°13’50.8”W, 21.vi.2023, R.P.R. Canejo col. (7♂, 15♀ brachypterous, 1♂ macropterous, CEAAVF).

#### Female terminalia.

Tergum VII with average width 3.7x length at midline; lateral margin sinuous, convex in basal half, constricted at base of lateral lobes; lateral lobes much longer than wide, strongly diverging at base, slightly converging at apex, bearing row of setae on lateral margin; posterior margin sinuous, bearing row of setae extending to halfway of mesal margins of lateral lobes (Fig 7N). Subgenital plate with tufts of long setae on apical 2/3 of lateral margins; lateral margins convex in basal 1/3; apical 2/3 convex, converging to narrowly rounded apex. Laterosternite VII convex at mesal margin, bearing two tufts of long setae posteriorly; proximal tuft as long as distal tuft; lateral margins sinuous; apex narrow, acute (Fig 9N). Mediotergite VIII with lateral margin convex, ventrally folded; posterior margin broadly rounded to truncate. Laterotergite VIII with length less than twice greatest width; apical half of lateral margin moderately serrated, bearing minute brush-like setae, converging posteriorly (Fig 11N). Valvifer I with mesal margin convex. Valvula I bearing robust, posteriorly directed setae, setae becoming longer towards apex; apical robust setae shorter than lateral margin (Fig 13N). Valvulae II slightly longer than wide; lateral margin slightly concave, with short setae; apex broadly rounded; with apical dark spot (Fig 15N). Valvula III flattened, sickle-shaped, with long dense pubescence on apical half, which is medially twisted; apex rounded. Mediotergite IX ogival, longer than valvula III; lateral margins parallel in basal 2/3; apical 1/3 converging to broadly rounded apex; length twice greatest width; width at base twice width of valvula III (Fig 17N).

#### Male genitalia.

Pygophore with elongate setae sparsely distributed over most of surface, except for a dense tuft on median region of posterior margin; anterior margin concave, posterior margin straight; Phallosoma straight in dorsal view, with apex slightly bent dextrally (Figs 21E–H). Paramere with anterior margin straight in apical half; mesal angle broadly rounded (Fig 24B).

#### Comments.

The record of *L. menkei* from the state of Rio Grande do Norte provided by Rodrigues & Sites [1] actually corresponds to *L. minutus*, according to what we describe here for these species. *Limnocoris minutus* is most similar to *L. burmeisteri, L. fittkaui* and *L. menkei*. The female terminalia of this species differ in the shape of the subgenital plate and mediotergite IX. The subgenital plate has the posterior margin distinctly narrowly rounded in *L. minutus* (Fig 9N), and not on the other species. The lateral margins of mediotergite IX are parallel in the basal half in *L. burmeisteri* (Fig 17E) and *L. menkei* (Fig 17M), and parallel in the basal 2/3 in *L. fittkaui* and *L. minutus*. However, mediotergite IX is about 1.5x as long as wide in the former (Fig 17H), whereas it is twice as long as wide in the latter (Fig 17N). The male genitalia of *L. minutus* differ from these other species only by the paramere with the mesal angle broadly rounded (Fig 24B).

***Limnocoris moreirai* Rodrigues & Sites, 2023**

(Figs 5E, 7O, 9O, 11O, 13O, 115O, 17O, 21I–L, 24C)

*Limnocoris moreirai* Rodrigues & Sites, 2023: 65–68, 72 (original description).

#### Type material examined.

All specimens brachypterous. PARATYPES: **BRAZIL, Amazonas**: São Gabriel da Cachoeira, Igarapé Muiá, 00º06’N, 66º52’W, 25.VIII.2011, R.L. Ferreira-Keppler, P.V. Cruz, A. Fernandes & E.A. Reis col. (1♂, 1♀, INPA).

#### Female terminalia.

Tergum VII with average width 3x the length at midline; lateral margin sinuous, slightly convex in basal half; posterior margin sinuous; lateral lobes much longer than wide, diverging at base, nearly parallel at apex, basal half of mesal margin bearing row of setae (Fig 7O). Subgenital plate with uniform pilosity; lateral margins parallel in basal 1/3; apical 2/3 concave, converging to broadly rounded apex. Laterosternite VII convex at mesal margin, bearing two tufts of long setae posteriorly; proximal tuft slightly shorter than distal tuft; lateral margins sinuous; apex acute (Fig 9O). Mediotergite VIII with lateral margin convex, ventrally folded; posterior margin broadly rounded to truncate. Laterotergite VIII with length less than twice the greatest width; apical half of lateral margin serrated, bearing minute brush-like setae, converging posteriorly (Fig 11O). Valvifer I with mesal margin convex. Valvula I bearing robust, posteriorly directed setae, setae becoming longer towards apex; apical robust setae shorter than lateral margin (Fig 13O). Valvulae II as long as wide; lateral margin slightly concave, with long setae; posterior margin distinctly broad; without apical dark spot (Fig 15O). Valvula III flattened, sickle-shaped, with long dense pubescence on apical half, which is medially twisted; apex rounded. Mediotergite IX ogival, longer than valvula III; lateral margins parallel in basal half; apical half converging to narrowly rounded apex; length twice greatest width; width at base twice the width of valvula III (Fig 17O).

#### Male genitalia.

Pygophore with elongate setae sparsely distributed over most of surface, except for a dense tuft on median region of posterior margin; anterior margin sinuous, posterior margin straight. Phallosoma straight, with apex slightly bent dextrally (Figs 21I–L). Paramere with anterior margin straight in apical half; mesal angle broadly rounded (Fig 24C).

#### Comments.

This species has the general aspect very similar to *L. burmeisteri*, *L. fittkaui* and *L. menkei* (see Rodrigues & Sites [1]), as well as the male and female terminalia. However, *L. moreirai* differs from these species by the absence of tufts of long golden setae on the posterolateral margins of the female subgenital plate (Fig 9O), and the shape of valvulae II, which has the posterior margin distinctly broad (Fig 15O).

***Limnocoris pauper* Montandon, 1897**

(Figs 5F, 7P, 9P, 11P, 13P, 15P, 17P, 21M–P, 24D)

*Limnocoris pauper* Montandon, 1897: 5 (original description).

*Limnocoris nigropunctatus* Montandon, 1909: 49–51 (original description) (**new synonym**).

*Limnocoris plaumanni* La Rivers, 1973: 4–7 (original description) (synonymized by Nieser & Lopez-Ruf, 2001: 289).

#### Type material examined.

All specimens brachypterous. HOLOTYPE of *L. pauper*, ♀ (BMNH), [**BRAZIL**], Brésil du Nord., Cumbase???, Limnocoris pauper Montand., type [18]97. HOLOTYPE of *L. nigropunctatus*, ♂ (UZMH), [**BRAZIL**, **Santa Catarina**], Brasilia, Blumenau, Limnocoris nigropunctatus, Montandon, type 1908, Mus. Zool. H: fors Spec. typ. No 7795, Limnocoris nigropunctatus Montandon, Mus. Hels. HOLOTYPE of *L. plaumanni*, ♀ (CAS), **BRAZIL**, [**Rio Grande do Sul**], Arroio Corneto, 29’45, 50’15 [= 29°45’S, 50°15’W], 800 meters, Fritz Plaumann, Apr 1959, Tainhas, R.G. do Sul, F. Plaumann, Limnocoris plaumanni Holotype ♀, Ira La Rivers Collection bequeathed to the California Academy of Sciences – 1978, California Academy of Sciences Type No. 13423. PARATYPES of *L. plaumanni*: Brazil, Arroio Corneto, 29’45, 50’15, 800 meters, Fritz Plaumann, Apr. 1959, Limnocoris plaumanni Paratype (4♂ including ‘allotype’, 4♀ CAS); Tainhas, R.G. do Sul, F. Plaumann, Arroio Corneto, 29’45, 50’15, 800 m (1♀ CAS).

#### Additional material examined. BRAZIL, Paraná.

Paranaguá, PR15, Parque Nacional Saint Hilaire/Lange, Cachoeira Quintilha, 25°38’28.9“S, 48°37’12.5”W, 125 m, 30.x.2023, R.P.R. Canejo, J.M.S. Rodrigues, M.S.L. Alexandre & L.D. Pereira col. (5♂, 11♀ brachypterous, 1♂, 1♀ macropterous, CEIOC); Morretes, PR12, Cachoeira do Jajá, 25°34’34.8”S, 48°49’43.6”W, 244 m, 30.x.2023, R.P.R. Canejo, J.M.S. Rodrigues, M.S.L. Alexandre & L.D. Pereira col. (6♂, 3♀ brachypterous, 1♂ macropterous, CEIOC); Tunas do Paraná, PR9, Cachoeirinha Tunas, 25°00’14.1”S, 49°01’30.1”W, 852 m, 27.x.2023, R.P.R. Canejo, J.M.S. Rodrigues, M.S.L. Alexandre & L.D. Pereira col. (9♂, 9♀ brachypterous, CEIOC); Campina Grande do Sul, PE [Parque Estadual] Pico do Paraná, Cachoeira do Arco Íris, antes da queda, 25°13’16.8”S, 48°51’26.0”W, 960 m, 31.x.2023, R.P.R. Canejo, J.M.S. Rodrigues, M.S.L. Alexandre & L.D.Pereira col. (12♂, 4♀ brachypterous, CEIOC). **São Paulo**: Capão Bonito, SP4, Reserva Particular do Patrimônio Natural Parque Taquaral da Mata Atlântica, Cachoeira do Taquaral, 24°05’17.0”S, 47°59’38.1”W, 760 m, 16.xi.2023, L.L. Dumas, J.M.S. Rodrigues & R.P.R. Canejo col. (7♂, 5♀ brachypterous, CEIOC); Capão Bonito, SP7, Parque Estadual Carlos Botelho, Cachoeira do Muriqui, 24°06’44.8”S, 47°59’44.0”W, 802 m, 17.xi.2023, L.L. Dumas, J.M.S. Rodrigues & R.P.R. Canejo col. (1♂, 1♀ brachypterous, CEIOC); Sete Barras, SP12, Parque Estadual Carlos Botelho, Ponte Aristides Manoel, Água da Vaca, cachoeira, 24°09’53.1”S, 47°58’54.6”W, 719 m, 18.xi.2023, L.L. Dumas, J.M.S. Rodrigues & R.P.R. Canejo col. (6♂, 4♀ brachypterous, CEIOC); Iporanga, SP20, Parque Estadual Intervales, Roda D’Água, cachoeira, 24°16’18.4”S, 48°25’29.8”W, 827 m, 21.xi.2023, L.L. Dumas, J.M.S. Rodrigues & R.P.R. Canejo col. (1♀ brachypterous, CEIOC); Iporanga, SP29, Parque Estadual Intervales, Cachoeira do Mirante, 24°16’44.2”S, 48°24’46.8”W, 812 m, 23.xi.2023, L.L. Dumas, J.M.S. Rodrigues & R.P.R. Canejo col. (1♀ brachypterous, CEIOC); Iporanga, SP34, Parque Estadual Intervales, Rio das Contas, 24°18’23.8”S, 48°24’51.8”W, 550 m, 04.xii.2023, L.L. Dumas, J.M.S. Rodrigues, L. Nery & L. Hoehne col. (1♂ brachypterous, CEIOC); Iporanga, SP35, Parque Estadual Intervales, Cachoeira da Água Comprida, 24°17’34.4”S, 48°25’03.4”W, 671 m, 04.xii.2023, L.L. Dumas, J.M.S. Rodrigues, L. Nery & L. Hoehne col. (3♂, 6♀ brachypterous, CEIOC); Ribeirão Grande, SP39, Rio das Almas, cachoeira, 24°09’26.6”S, 48°21’07.6”W, 699 m, 05.xii.2023, L.L. Dumas, J.M.S. Rodrigues, L. Nery & L. Hoehne col. (4♂, 4♀ brachypterous, CEIOC); Sete Barras, SP42, Parque Estadual Carlos Botelho, Cachoeira do Quilombo, 24°12’34.6”S, 48°03’14.8”W, 147 m, 07.xii.2023, L.L. Dumas, J.M.S. Rodrigues, L. Nery & L. Hoehne col. (3♂ brachypterous, 3♂, 4♀ macropterous, CEIOC); Salesópolis, Estação Biológica da Boracéia, Ribeirão Coruja, [−23.65, −45.91], 28.v.1965, C.G. Froehlich col. (1♂ brachypterous, CEIOC); Salesópolis, Estação Biológica da Boracéia, Ribeirão Venerando, [−23.658, −45.745], 30.x.1991, EXC. BIZ 721 col. (1♂ brachypterous, CEIOC); Salesópolis [sic, Bertioga], SABESP, Ribeirão Coruja, [−23.668, −45.897], 25.ix.1987, C.G. Froehlich col. (6♀ brachypterous, CEIOC).

#### Female terminalia.

Tergum VII with average width 3x length at midline; lateral margins sinuous, convex at mid-length; lateral lobe longer than wide, bearing longer setae on posterolateral margin; minute brush-like setae sometimes present; posterior margin sinuous (Fig 8P). Subgenital plate with uniform pilosity; lateral margins parallel in basal 1/3, converging in apical 2/3 to broadly rounded apex; posterior margin sometimes with small concavity. Laterosternite VII convex at mesal margin, bearing single tuft of long setae posteriorly; lateral margin sinuous; apex acuminate (Fig 10P). Mediotergite VIII with lateral margin convex; posterior margin rounded. Laterotergite VIII with length 2.2x greatest width; apical half of lateral margin weakly serrated, bearing minute brush-like setae, converging posteriorly (Fig 12P). Valvifer I with mesal margin convex. Valvula I bearing robust, posteriorly directed setae, setae becoming longer towards apex; apical robust setae slightly shorter than lateral margin (Fig 14P). Valvulae II slightly wider than long; lateral margins convex, bearing long setae; apex broadly rounded, bearing apical dark spot (Fig 16P). Valvula III flattened, sickle-shaped, with long dense pubescence on dorsal surface; apical half medially twisted. Mediotergite IX ogival, shorter than valvula III; lateral margins converging from base to broadly rounded apex; length 1.4x greatest width; width at base less than twice the width of valvula III (Fig 18P).

#### Male genitalia.

Pygophore with elongate setae sparsely distributed over most of surface, except for a dense tuft on median region of posterior margin; anterior and posterior margins straight. Phallosoma straight, with apex slightly bent dextrally (Figs 21M–P). Paramere with anterior margin concave at medial half; mesal angle narrowly rounded (Fig 24D).

#### Comments.

Nieser [15], in his study of the Nepomorpha of Suriname and adjacent regions, examined the female holotype of *L. pauper* and presented a brief redescription of this species. However, based on the information provided in the original description and on the labels associated with the holotype, he was unable to determine the type locality. Nieser [15] suggested that it is located in northeastern Brazil, while Moreira *et al*. [16] noted the department of San Martín, Peru, as a possible origin. However, all geographic records subsequent to the original description are located in southeastern and southern Brazil [16].

Nieser [15] had already noted the morphological similarity between *L. pauper* and *L. nigropunctatus* (described from specimens from Santa Catarina, southern Brazil), but did not propose their synonymy because of the supposed distance between the type localities. Nieser & López-Ruf [2], however, in their review of the genus, maintained *L. nigropunctatus* as a valid species and considered *L. sattleri* as its junior synonym. In the present study, we examined the holotypes of *L. pauper*, *L. nigropunctatus*, and *L. sattleri*, which allowed us to verify a distinct morphological similarity between the types of *L. pauper* and *L. nigropunctatus*, with no significant differences between them. Thus, *L. nigropunctatus* is proposed here as a junior synonym of *L. pauper*. In contrast, *L. sattleri* is clearly distinct from *L. pauper* and is here resurrected as a valid species (see below).

Although *L. pauper* shares some similarities in the female terminalia with the other species from Brazil without a longitudinal row of golden setae on the mesosternum, it can be distinguished based on the following combination of characteristics: the posterior margin of tergum VII sinuous (Fig 7P) and mediotergite IX shorter than valvula III (Fig 17P). No character in the male genitalia presented consistent differences to distinguish this species from similar congeners.

***Limnocoris pusillus* Montandon, 1897**

(Figs 5G, 8A, 10A, 12A, 14A, 16A, 18A, 21Q–T, 25E)

*Limnocoris pusillus* Montandon, 1897: 7–8 (original description).

*Limnocoris mansosotoi* De Carlo, 1951: 45–46 (original description) (synonymized by Nieser & López-Ruf, 2001: 293).

*Limnocoris vianai* De Carlo, 1967: 186–187 (original description) (synonymized by Nieser & López-Ruf, 2001: 293).

#### Material examined.

All specimens brachypterous. **BRAZIL, Espírito Santo**: Sooretama, REBIO [Reserva Biológica de] Sooretama, Afluente do Rio Novo, SO17, –18.9352, –40.2211, 38 m, 11.v.2024, A.P. Pinto, A.P.M. Santos, L. Hoehne, N.O. Paiva, R.P.R. Canejo col. (7♂, 7♀, CEIOC); Sooretama, REBIO Sooretama, Córrego Rodrigues, SO03, 19°01’36.5“S, 40°13’39.3”W 60 m, 12.v.2024, A.P. Pinto, A.P.M. Santos, L. Hoehne, N.O. Paiva, R.P.R. Canejo col. (9♂, 8♀, CEIOC).

#### Female terminalia.

Tergum VII with average width 2.3x length at midline; lateral margins convex; posterior margin straight or shallowly concave; lateral lobe slightly longer than wide, bearing longer setae on posterolateral margin (Fig 8A). Subgenital plate with uniform pilosity; lateral margins parallel in basal 1/3; apical 2/3 convex at mid-length, margins at posterior 1/3 straight, converging to narrowly rounded apex. Laterosternite VII convex at mesal margin, bearing single tuft of long setae posteriorly; lateral margin concave at mid-length; apex acuminate (Fig 10A). Mediotergite VIII with lateral margin sinuous; posterior margin broadly rounded. Laterotergite VIII with length twice greatest width; basal half of lateral margin distinctly convex at base, constricted near mid-length; apical half of lateral margin weakly serrated, bearing large brush-like setae (Fig 12A). Valvifer I with mesal margin convex. Valvula I bearing robust, posteriorly directed setae, setae becoming longer towards apex; apical robust setae slightly shorter than lateral margin (Fig 14A). Valvulae II as long as wide; lateral margin straight, bearing short setae; apex broadly rounded, without apical dark spot (Fig 16A). Valvula III flattened, sickle-shaped, with long dense pubescence on dorsal surface; apical half medially twisted. Mediotergite IX slightly longer than valvula III; lateral margins parallel in basal 1/4, slightly wider at level of anal operculum, converging to narrowly rounded apex; dense setae on dorsal surface longer at apex; length 1.8x greatest width; width at base less than twice width of valvula III (Fig 18A).

#### Male genitalia.

Pygophore with elongate setae sparsely distributed over most of surface, except for a dense tuft on median region of posterior margin; anterior margin concave, posterior margin straight. Phallosoma straight in dorsal view, with apex not surpassing the level of the ventral lobes (Figs 21Q–T). Parameres with anterior margin shallowly concave at medial half; median angle broadly rounded (Fig 24E).

#### Comments.

The female terminalia of this species are most similar to those of *L. illiesi, L. reynosoi* Rodrigues & Sites, 2021 and *L. yanomami* Rodrigues & Sites, 2023. However, *L. pusillus* differs from them in the following characters. In *L. pusillus*, the lateral margins of tergum VII are convex (Fig 8A), whereas they are straight in *L illiesi* and *L. reynosoi* (Figs 7I; 8B), and divergent in *L. yanomami* (Fig 8I). The subgenital plate has the apical 2/3 of the lateral margin convex at midlength in *L. pusillus* (Fig 10A), whereas in *L. illiesi* it is concave at midlength (Fig 9I), and in *L. yanomami* it is nearly straight in apical 2/3 (Fig 10I). Also, mediotergite IX is shorter than valvulae III in *L. illiesi, L. reynosoi* and *L. yanomami* (Figs 17I; 18B; 18I), whereas in *L. pusillus* it is slightly longer (Fig 18A). As for the male genitalia, no structure has consistent differences to distinguish it from the other species.

***Limnocoris reynosoi* Rodrigues & Sites, 2021**

(Figs 5H, 8B, 10B, 12B, 14B, 16B, 18B, 21U–X, 24F)

*Limnocoris reynosoi* Rodrigues & Sites, 2021: 80–82, 88 (original description).

#### Material examined.

All specimens brachypterous. **BRAZIL, Roraima**: Município Iracema, Rio Ajaraní, Reserva Yanomami, 01°59’51“N, 61°30’40”W, 03.III.1999, V. Py-Daniel, U. Barbosa & O.S. Silva col. (3♂, 1♀, MZUSP).

#### Female terminalia.

Tergum VII with mean width 2.2x length at midline; lateral margins straight; posterior margin nearly straight; lateral lobes rounded, longer than wide, slightly diverging (Fig 8B). Subgenital plate quadrate, with uniform pilosity; lateral margins parallel, posterior margin nearly straight. Laterosternite VII convex at mesal margin, bearing two tufts of long setae posteriorly, proximal tuft shorter than distal tuft; lateral margins straight; apex broadly rounded (Fig 10B). Mediotergite VIII with lateral margin straight; posterior margin broadly rounded. Laterotergite VIII with length about twice the greatest width; apical half of lateral margin weakly serrated, bearing large brush-like setae, converging posteriorly (Fig 12B). Valvifer I with mesal margin convex. Valvula I bearing robust, posteriorly directed setae; apical robust setae shorter than lateral margin (Fig 14B). Valvulae II longer than wide; lateral margin straight, bearing long setae; apex broadly rounded, without apical dark spot (Fig 16B). Valvula III flattened, sickle-shaped, dorsal surface with long dense pubescence near apex; apical half medially twisted; broadly rounded. Mediotergite IX ogival, slightly longer than valvula III; lateral margins convex, converging to broadly rounded apex; length 1.5x greatest width; width at base less than twice width of valvula III (Fig 18B).

#### Male genitalia.

Pygophore with elongate setae sparsely distributed over most of surface; anterior margin slightly concave, posterior margin straight. Phallosoma straight (Figs 21U–X). Parameres long, narrow, with anterior margin concave in apical half; mesal angle narrowly rounded (Fig 24F).

#### Comments.

This species has the overall morphology and female terminalia most similar to those of *L. illiesi*, *L. pusillus* and *L. yanomami* (see Rodrigues & Sites [1,4] for other aspects of the morphoplogy). *Limnocoris reynosoi* can be distinguished from all of these species by the distinct shape of the subgenital plate, which is quadrate with straight lateral margins and a very broad, nearly straight, posterior margin (Fig 10B). It also differs by the presence of a short subapical tuft of setae on the laterosternite VII, which is absent in the others (Fig 10B). No consistent differences were found in the male genitalia to distinguish this species. However, the paramere is much narrower than in the similar species (Fig 24F).

***Limnocoris rotundatus* De Carlo, 1951**

(Figs 5I, 8C, 10C, 12C, 14C, 16C, 18C, 22A–D, 24G)

*Limnocoris rotundatus* De Carlo, 1951: 48–49 (original description).

#### Material examined.

All specimens brachypterous. **BRAZIL, Paraná**: Jaguariaíva, PR5, Rio Diamante, 24°21’31.9“S, 49°48’45.4”W, 1127 m, 26.x.2023, R.P.R. Canejo, J.M.S. Rodrigues, M.S.L. Alexandre & L.D. Pereira col. (16♂, 16♀, CEIOC).

#### Female terminalia.

Tergum VII with average width twice length at midline; lateral margin sinuous, convex at mid-length; posterior margin slightly convex; lateral lobes trapezoid, as wide as long; posterolateral margins of lateral lobes converging, bearing longer setae, brush-like setae sometimes present (Fig 8C). Subgenital plate with fringe of longer setae on posterior margin; lateral margins slightly divergent in basal half; apical half broadly convex, converging to semicircular posterior margin. Laterosternite VII convex on mesal margin, bearing single tuft of long setae posteriorly; lateral margin shallowly concave, strongly projected laterally near apex, forming a posterior, concave margin; apex sharply acuminate (Fig 10C). Mediotergite VIII with lateral margin straight; posterior margin broadly rounded. Laterotergite VIII with length twice greatest width; apical half of lateral margin weakly serrated, bearing minute brush-like setae, converging posteriorly (Fig 12C). Valvifer I with mesal margin nearly straight. Valvula I bearing robust, posteriorly directed setae, setae becoming longer towards apex; apical robust setae longer than lateral margin (Fig 14C). Valvulae II slightly wider than long; lateral margin slightly sinuous, with short setae; apex acuminate, without apical dark spot (Fig 16C). Valvula III thickened, baton-shaped, with uniform width; dorsal surface with long dense pubescence; apex broadly rounded, with dense tuft of long setae; posterior region of ventral surface with diagonal indentation. Mediotergite IX ogival, shorter than valvula III; lateral margins convex, converging to broadly rounded apex; length 1.3x greatest width; width at base less than twice width of valvula III (Fig 18C).

#### Male genitalia.

Pygophore with elongate setae sparsely distributed over most of the surface, except for a dense tuft on median region of posterior margin; anterior and posterior margins straight. Phallosoma straight (Figs 22A–D). Paramere with anterior margin shallowly concave in apical half; mesal angle narrowly rounded (Fig 25G).

#### Comments.

The female terminalia of this species share a similarity with those of *L. admontandoni*, *L. insignis* and *L. sattleri*, in that valvula III has the posterior region of ventral surface indented (Figs 17J; 18C, D; 26F). It can be distinguished from *L. admontandoni* by the head not strongly projected anteriorly (Fig 25B). Also, *L. admontandoni* has the valvula III strongly indented, causing it to fold over the posterior margin of the subgenital plate (Figs 25E; 26F). It can be separated from *L. insignis* and *L. sattleri* by the shape of the laterosternite VII, which is strongly projected in the lateral margin in *L. rotundatus,* causing it to form a concave posterior margin with the apex (Fig 10C). In *L. insignis* and *L. sattleri*, it is not as projected to form a posterior margin (Figs 9J; 10D). It also differs from the other three species by the shape of the lateral margins of the subgenital plate, which are semicircular in the posterior half in *L. roduntatus* (Fig 10C); broadly convex in *L. admontandoni* (Fig 26B) and *L. insignis* (Fig 9J), and straight in *L. sattleri* (Fig 10D). No consistent differences were found in the male genitalia of these similar species.

***Limnocoris sattleri* De Carlo, 1966**

(Figs 6A, 8D, 10D, 12D, 14D, 16D, 18D, 22E–H, 24H)

*Limnocoris sattleri* De Carlo, 1966: 113 (original description) **stat. restit.**

*Limnocoris sattleri*: Nieser & Lopez-Ruf 2001: 285 (synonymized with *L. nigropunctatus*).

#### Type material examined.

All specimens brachypterous. HOLOTYPE ♂ (ZSMC), [**BRAZIL**], **Minas Gerais**, Levantina, Ibicatu, 13.8. [19]63, leg. W. Sattler, Sa 497, Limnocoris sattleri De Carlo ♂, Museo Argentino de Ciencias Naturales, Holotypus, Sammlg. H. Weber. PARATYPE: same data as holotype, except: Allotypus ♀ (1♀ ZSMC).

#### Additional material examined.

All specimens brachypterous. **BRAZIL, Minas Gerais**: [Itamonte], 828, Serra Negra, Rio Aiuruoca, Sedimento, Areia, 1500 m, [–22.34, –44.69], 31.iv.1991, J.L. Nessimian col. (1♀, DZRJ); Bocaina de Minas, Serra Negra, 1149, Córrego do Morro Cavado, 1200 m, [–22.31, –44.61], 02.vi.1991, J.L. Nessimian **Rio de Janeiro**: 885, Maringá, Rio Preto, Litter Fundo, [–22.328, –44.580] 20.x.1997, Eq. Entomologia (1♂, DZRJ). **São Paulo**: 5622, Rio Mambucaba (Cachoeira), Estrada para o Pico Tira Chapéu, S.[ão] José do Barreiro, 22°43’38“S, 44°37’58.40”W, 1524 m, 28–31.viii.2015, N. Ferreira-Jr., L.L. Dumas, H. Costa, B. Guimarães col. (1♀, DZRJ).

#### Female terminalia.

Tergum VII with average width twice length at midline; lateral margin sinuous, convex at mid-length; posterior margin nearly straight; lateral lobes trapezoid, as wide as long; posterolateral margins of lateral lobes converging, bearing longer setae, brush-like setae sometimes present (Fig 8D). Subgenital plate with fringe of longer setae on posterior margin; lateral margins slightly divergent in basal half; nearly straight in apical half, converging to broadly rounded posterior margin. Laterosternite VII sinuous at mesal margin, bearing single tuft of long setae posteriorly; lateral margin shallowly concave, projected laterally near apex; apex sharply acuminate (Fig 10D). Mediotergite VIII with lateral margin straight; posterior margin broadly rounded. Laterotergite VIII with length twice greatest width; apical half of lateral margin weakly serrated, bearing minute brush-like setae, converging posteriorly (Fig 12D). Valvifer I with mesal margin nearly straight. Valvula I bearing robust, posteriorly directed setae, setae becoming longer towards apex; apical robust setae longer than lateral margin (Fig 14D). Valvulae II slightly wider than long; lateral margin slightly sinuous, with short setae, setae becoming longer towards apex; apex rounded, sometimes with apical dark spot (Fig 16D). Valvula III thickened, baton-shaped; dorsal surface with long dense pubescence; apex broadly rounded, with dense tuft of long setae; posterior region of ventral surface with shallow diagonal indentation. Mediotergite IX ogival, shorter than valvula III; lateral margins convex, converging to broadly rounded apex; length 1.3x greatest width; width at base subequal to width of valvula III (Fig 18D).

#### Male genitalia.

Pygophore with elongate setae sparsely distributed over most of its surface, except for a dense tuft on median region of posterior margin; anterior and posterior margins straight. Phallosoma straight (Figs 22E–H). Paramere with anterior margin shallowly concave in apical half; mesal angle narrowly rounded (Fig 24H).

#### Comments.

De Carlo (1966) described *L. sattleri* on the basis of specimens from two different localities: Minas Gerais, where the holotype comes from, and Rio Grande do Sul. Later, Nieser & López-Ruf (2001) examined the type series and concluded that the specimens from Rio Grande do Sul actually belonged to a different species, which they then described as *L. decarloi*. In the same study, however, the authors considered *L. sattleri* as a junior synonym of *L. nigropunctatus*. In the present study, after examination of the type specimens of these species, we found that *L. sattleri* is not conspecific with the holotype of *L. nigropunctatus*, as it has several distinct morphological characteristics. Therefore, *L. sattleri* is here resurrected as a valid species.

The female terminalia of this species shares a similarity with *L. admontandoni*, *L. insignis* and *L. rotundatus*: valvula III with the posterior region of the ventral surface indented (Figs 17J; 18C–D; 26F). However, in *L. sattleri*, this indentation is very shallow, and noticeable only in lateral view (Fig 18D). It also differs from the other species by having the lateral margins of the subgenital plate straight in the apical half (Fig 10D). No consistent differences were found in the male genitalia of this species to distinguish it from the others.

***Limnocoris siolii* (De Carlo, 1966)**

(Figs 6B, 8E, 10E, 12E, 14E, 16E, 18E, 22I–L, 24I)

*Sattleriella siolii* De Carlo, 1966: 112–113 (original description).

*Limnocoris siolii*: Nieser & Lopez-Ruf 2001: 265, 297–298, 319 (changed combination).

#### Material examined.

All specimens brachypterous. **BRAZIL, Rio de Janeiro**: [Itatiaia], 5618, Parna [Parque Nacional] Itatiaia, Riacho na Trilha Vinicius de Moraes, [–22.452, –44.605], 18.XI.2021, B. Clarkson, L.G. Golçalves, A.F. Antunes, A.L. Ferreira & M.S.L. Alexandre col. (1♂, 1♀, DZRJ); Nova Friburgo, 4275, Rio São Lourenço, Pedra Solta, [–22.33, –42.58], 04.x.2000, (10♂, 12♀, DZRJ); Teresópolis, 1082, Vale da Revolta, Rio Paquequer, [–22.44, –42.94], 11.1.1990, Eq. Entomologia col. (1♂, DZRJ). **São Paulo**: 5623, São José do Barreiro, P.N. [Parque Nacional] da Serra Bocaina, Ribeirão da Prata, BOC07, Folhiço e areia, [–22.780, –44.611], 01.ix.2012, P.M. Souto, M.R. Souza & J.L. Nessimian col. (4♂, 4♀, DZRJ).

#### Female terminalia.

Tergum VII with average width 2.5x length at midline; lateral margins convex at midline, bearing long setae on posterior half; posterior margin straight; lateral lobe triangular, as long as wide, bearing longer setae on posterolateral margin (Fig 8E). Subgenital plate with uniform pilosity, lateral margins parallel in basal 1/3, converging to rounded apex at disal 2/3. Laterosternite VII convex at mesal margin, bearing a single tuft of long setae posteriorly; lateral margins straight; apex acute (Fig 10E). Mediotergite VIII with lateral margin straight; posterior margin rounded. Laterotergite VIII narrow; length about three times greatest width; apical half of lateral margin weakly serrated, bearing large brush-like setae (Fig 12E). Valvifer I with mesal margin straight. Valvula I bearing robust, posteriorly directed setae, setae becoming longer towards apex; apical robust setae shorter than lateral margin (Fig 14E). Valvulae II as long as wide; lateral margins straight, with short setae; apex broadly rounded, bearing apical dark spot (Fig 16E). Valvula III flattened, with sparse setae throughout dorsal surface. Mediotergite IX triangular, longer than valvula III; lateral margins nearly straight, converging to narrowly rounded apex; length 1.5x times greatest width; width at base about twice width of valvula III (Fig 18E).

#### Male genitalia.

Pygophore with elongate setae sparsely distributed over most of surface, with anterior and posterior margins nearly straight. Phallosoma straight (Figs 22I–L). Paramere with anterior margin straight in apical half; mesal angle broadly rounded (Fig 24I).

#### Comments.

The female terminalia of this species are most similar to those of *L. acutalis*, which is a very similar species in general. The subgenital plate, valvulae II–III and mediotergite IX are nearly identical in these species, and they also have a uniquely shaped laterotergite VIII, which is very narrow and with very large brush-like setae on the posterior margin (Figs 11A; 12E). However, their terminalia can be distinguished as follows: the posterior margin of tergum VII is straight and the lateral lobes are triangular in *L. siolii* (Fig 8E), whereas in *L. acutalis* the posterior margin is strongly convex and the lateral lobes are rounded (Fig 7A). The posterior part of valvula I (beyond the posterior margin of valvifer I) is as wide as, or wider than long in *L. siolii* (Fig 14E), whereas in *L. acutalis* it is longer than wide (Fig 13A). In the male genitalia, the phallosoma of *L. siolii* is broad and short, with the apex barely surpassing the level of the dorsolateral projections of the genital capsule (Figs 22I–L), whereas it is narrow and long in *L. acutalis*, with the ventral lobes surpassing the level of the dorsolateral projections of the genital capsule (Figs 19A–D).

***Limnocoris submontandoni* La Rivers, 1974**

(Fig 6C, 8F, 10F, 12F, 14F, 16F, 18F, 22M–P, 24J)

*Limnocoris submontandoni* La Rivers, 1974: 10–11 (original description).

#### Material examined.

All specimens brachypterous. **BRAZIL, Espírito Santo**: Domingos Martins, Alto Galo, 20°17’15.0“S, 40°38’30.0”W, 602 m, 05.i.2019, H.D.D. Rodrigues col. (4♂, 3♀, CEIOC). **Minas Gerais**: Luminárias, Ribeirão da Ponte, Próximo de Luminárias, 21°31.008’S, 44°52.269’W, 966 m, 03.xii.2016, H.D.D. Rodrigues col. (5♀, CEIOC); Cachoeira do Lobo, Perto do Capitólio, L-1976, 20°37’35.2”S, 45°55’28.5”W, 873 m, 30.xi.2016, H.D.D. Rodrigues col. (1♂, CEIOC); **Rio de Janeiro**: 900, Nova Friburgo, Rio Macaé de Cima, [–22.39, –42.49], 02-II-1992, J.L. Nessimian & L.F. Dorvillé col. (1♀, DZRJ); 1130, Teresópolis, Rio dos Frades, Sedimento, [–22.33, –42.78], 16.vi.1991, Eq. Entomologia col. (1♂, DZRJ); same data, except 1132, Fundo Arenoso Fino, 16.ii.1991 (4♂, 1♀, DZRJ); same data, except 1138, Margem Areia Grossa, L.F. Dorvillé, E.R. Silva, J.L. Nessimian col. (2♂, 1♀, DZRJ); same data, except 1137, Folhiço, Eq. Entomologia col. (1♂, DZRJ); same data, except 1135, E.R. Silva, L.F. Dorvillé & J.L. Nessimian col. (6♂, 3♀, DZRJ); same data, except 1148J.L. Nessimian & L.F. Dorvillé col. (2♀, DZRJ); **São Paulo**: Sete Barras, SP14, Rio Preto, Rede D, –24.1931, –47.8906, 30 m, 19.xi.2023, L.L. Dumas, J.M.S. Rodrigues, R.P.R. Canejo col. (1♂, 1♀, CEIOC).

#### Female terminalia.

Tergum VII with average width twice length at midline; lateral margin sinuous, convex at mid-length; posterior margin slightly convex; lateral lobes rounded, length twice the width; posterolateral margin bearing longer setae (Fig 8F). Subgenital plate with fringe of sparse, longer setae on posterior margin; lateral margins slightly divergent in basal 1/3; apical 2/3 converging to broadly rounded apex. Laterosternite VII convex at mesal margin, bearing single tuft of long setae posteriorly; lateral margin nearly straight; apex rounded (Fig 10F). Mediotergite VIII with lateral margin concave; posterior margin broadly rounded. Laterotergite VIII with length twice greatest width; apical half of lateral margin weakly serrated, bearing minute brush-like setae, converging posteriorly (Fig 12F). Valvifer I with mesal margin nearly straight. Valvula I bearing robust, posteriorly directed setae, setae becoming longer towards apex; apical robust setae longer than lateral margin (Fig 14F). Valvulae II slightly wider than long; lateral margin straight, with long setae; apex acuminate, without apical dark spot (Fig 16F). Valvula III thickened, cone-shaped; dorsal surface with long dense pubescence; apex broadly rounded. Mediotergite IX ogival, shorter than valvula III; lateral margins convex, converging to broadly rounded apex; length 1.8x greatest width; width at base less than twice width of valvula III (Fig 18F).

#### Male genitalia.

Pygophore with elongate setae sparsely distributed over most of surface, with anterior and posterior margins nearly straight. Phallosoma straight (Figs 22M–P). Parameres with anterior margin straight in apical half; mesal angle broadly rounded (Fig 24J).

#### Comments.

This species is very similar to *L. abbreviatus* Nieser & Lopez-Ruf, 2001 and *L. montandoni* La Rivers 1974 [2,12]. Unfortunately, we could not examine any material of the last two species to provide a more useful comparison of characters in the female terminalia. Nonetheless, *L. submontantoni* shares a few similarities in the female terminalia with the congeners from southern and southeastern Brazil, such as valvulae I with the apical setae longer than the lateral margin (Fig 14F), valvula III thickened and mediotergite IX shorter than valvula III (Fig 18F). However, it can be distinguished from the other similar species by the following combination of characters: subgenital plate with fringe of sparse, longer setae on posterolateral margin; lateral margin slightly divergent in basal 1/3, converging in apical 2/3 to a broadly rounded apex; and laterosternite VII with lateral margin straight and apex rounded (Fig 8F). The male genitalia have no distinct structure to distinguish it from any other species examined in this study.

***Limnocoris surinamensis* La Rivers, 1974**

(Figs 6D, 8G, 10G, 12G, 14G, 16G, 18G)

*Limnocoris fittkaui surinamensis* Nieser, 1975: 70–72 (original description).

*Limnocoris surinamensis*: Rodrigues & Sites 2023: 68–70 (status changed).

#### Material examined. Brazil, Amapá.

[Porto Grande], BR-201 [sic, BR-210], Igarapé Munguba, [0.662, –51.858], 23.iii.1991, V.Py-Daniel & U. Barbosa (1♀ brachypterous, MZUSP).

#### Female terminalia.

Tergum VII with average width 3x length on medial line; lateral margins sinuous, convex in basal half; posterior margin sinuous; lateral lobes much longer than wide, divergent, bearing row of dense setae in basal 2/3 of lateral margin; mesal margin and apical 1/3 of lateral margin bearing sparse long setae (Fig 8G). Subgenital plate with tufts of long setae on apical half of lateral margins; lateral margins parallel in basal half; apical halves concave, converging to broadly rounded apex. Laterosternite VII convex at mesal margin, bearing two tufts of long setae posteriorly; proximal tuft as long as distal tuft; lateral margins sinuous; apex narrow (Fig 10G). Mediotergite VIII with lateral margin convex, ventrally folded; posterior margin broadly rounded. Laterotergite VIII with length less than twice greatest width; apical half of lateral margin moderately serrated, bearing minute brush-like setae, converging posteriorly (Fig 12G). Valvifer I with mesal margin convex. Valvula I bearing robust, posteriorly directed setae, setae becoming longer towards apex; apical robust setae shorter than lateral margin (Fig 14G). Valvulae II slightly longer than wide; lateral margin convex, with long setae; apex broadly rounded, with apical dark spot (Fig 16G). Valvula III flattened, sickle-shaped, with long dense pubescence on apical half, which is medially twisted; apex narrowly rounded. Mediotergite IX ogival, longer than valvula III; lateral margins convex in basal half, converging in apical half to broadly rounded apex; length 1.4x the greatest width; width at base twice width of valvula III (Fig 18G).

#### Comments.

The female terminalia of this species are nearly identical to those of *L. machrisi* and *L. volxemi*. However, *L. surinamensis* lacks the cluster of short stout setae on the mesal margin of the apex of valvula I, which is present in the two other species (Figs 13K; 14H). No differences were found in the other structures of the female terminalia.

***Limnocoris volxemi* (Lethierry, 1877)**

(Figs 6E, 8H, 10H, 12H, 14H, 16H, 18H, 22Q–T, 24K)

*Borbocoris volxemi* Lethierry, 1877: 41 (original description).

*Limnocoris volxemi*: Montandon 1897: 2–3 (changed combination).

*Limnocoris maculiceps* Montandon, 1898: 417, 424–425 (original description) (synonymized by Nieser *et al*. 2013: 342).

#### Material examined. BRAZIL, Bahia.

Mucugê, Parque Nacional da Chapada Diamantina, Cachoeira Véu da Noiva, 13°17’17.2“S, 41°16’05.7”W, 776 m, 06.vi.2021, J.M.S. Rodrigues & J.F. Barbosa col. (1♀ brachypterous, 1♀ macropterous, CEIOC); Palmeiras, Parque Nacional da Chapada Diamantina, Rio Preto, 12°36’12.8”S, 41°31’30.0”W, 820 m, 05.v.2021, J.M.S. Rodrigues & J.F. Barbosa col. (2♂, 2♀ brachypterous, CEIOC); [Palmeiras], Parque Nacional Chapada Diamantina, córrego próximo ao Morrão, [–12.53, –41.48], 10.i.1994, A. Jerozolimski col. (2♀ brachypterous, CEIOC); **Minas Gerais**: Ouro Preto, Bicicleta, [−20.3466, −43.4950], 12.ii.2019, G.L.V. Machado col. (3♀ brachypterous, CEIOC); same data, except 27.vii.2018 (2♂, 2♀ brachypterous, CEIOC); Ouro Preto, Jesus, [−20.3622, −43.4886], 27.iv.2018, G.L.V. Machado G.L.V. (1♀ brachypterous, CEIOC); Ouro Preto, Folhinha, [−20.3502, −43.4900], 24.viii.2018, G.L.V. Machado col. (1♂, 1♀ brachypterous, CEIOC); [Guapé], Cachoeira do Lobo, Perto do Capitólio, L-1976, 20°37’35.2”S, 45°55’28.5”W, 873 m, 30.xi.2016, H.D.D. Rodrigues col. (1♂ brachypterous, CEIOC).

#### Female terminalia.

Tergum VII with average width 3x length on medial line; lateral margins sinuous, convex in basal half; posterior margin sinuous; lateral lobes much longer than wide, divergent, bearing row of dense setae on basal 2/3 of lateral margin; mesal margin and apical 1/3 of lateral margin bearing sparse long setae (Fig 8H). Subgenital plate with tufts of long setae on apical half of lateral margins; lateral margins parallel in basal half; apical halves concave, converging to broadly rounded apex. Laterosternite VII convex at mesal margin, bearing two tufts of long setae posteriorly; proximal tuft as long as distal tuft; lateral margins sinuous; apex acute (Fig 10H). Mediotergite VIII with lateral margin convex, ventrally folded; posterior margin broadly rounded. Laterotergite VIII with length less than twice greatest width; apical half of lateral margin serrated, bearing minute brush-like setae, converging posteriorly (Fig 12H). Valvifer I with mesal margin convex. Valvula I bearing robust, posteriorly directed setae, setae becoming longer towards apex; apical robust setae shorter than lateral margin; apex with cluster of short stout setae on mesal margin (Fig 14H). Valvulae II slightly longer than wide; lateral margin convex, with long setae; apex broadly rounded, with apical dark spot (Fig 16H). Valvula III flattened, sickle-shaped, with long dense pubescence on apical half, which is medially reflected; apex narrowly rounded. Mediotergite IX ogival, longer than valvula III; lateral margins parallel in basal 2/3, converging in apical 1/3 to narrowly rounded apex; length 1.7x the greatest width; width at base twice width of valvula III (Fig 18H).

#### Male genitalia.

Pygophore with elongate setae sparsely distributed over most of its surface, except for a dense tuft on median region of posterior margin; anterior margin concave, posterior margin convex. Phallosoma straight, with apex slightly bent dextrally (Figs 22Q–T). Paramere with anterior margin concave in apical half; mesal angle narrowly rounded, curved anteriorly (Fig 24K).

#### Comments.

The female terminalia of this species are nearly identical to those of *L. machrisi*, which is also very similar in general morphology, differing only in body size and embolium shape. They also share an exclusive trait, which is the apex of valvulae I with a cluster of short stout curved setae on mesal margin (Figs 13K; 14H). No consistent differences were found on the male or female genitalia of these two species.

***Limnocoris yanomami* Rodrigues & Sites, 2023**

(Fig 6F, 8I, 10I, 12I, 14I, 16I, 18I, 22U–V, 24L)

*Limnocoris yanomami* Rodrigues & Sites, 2023: 70–74 (original description).

#### Type material examined. BRAZIL, Roraima.

PARATYPE. [Alto Alegre], Parque Indígena Xitei/Xidea, Rio Thirei-ú [2150], [2.60, −63.87], 18.vi.1997, V. Py-Daniel & U. Barbosa (1♀ brachypterous, MZUSP).

#### Additional material examined.

All specimens brachypterous. Same data as type material, (9♂, 1♀, CEIOC).

#### Female terminalia.

Tergum VII with average width 3x length at midline; lateral margins straight, distinctly divergent in apical half; posterior margin straight; lateral lobe quadrate, as long as wide, bearing longer setae on posterolateral margin (Fig 8I). Subgenital plate with uniform pilosity; lateral margins diverging in basal 1/3; apical 2/3 straight, converging to broadly rounded apex. Laterosternite VII convex on mesal margin, bearing single tuft of long setae posteriorly; lateral margin convex near mid-length, strongly projected laterally near posterior end, forming a posterior, concave margin with the apex; apex acute (Fig 10I). Mediotergite VIII with lateral margin convex; posterior margin broadly rounded. Laterotergite VIII with length less than twice greatest width; basal half of lateral margin distinctly convex at base, shallowly concave near mid-length; apical half of lateral margin perpendicular to longitudinal axis, weakly serrated, bearing large brush-like setae (Fig 12I). Valvifer I with mesal margin convex. Valvula I bearing robust, posteriorly directed setae, setae becoming longer towards apex; apical robust setae shorter than lateral margin (Fig 14I). Valvulae II longer than wide; lateral margin straight, bearing long setae; apex broadly rounded, with apical dark spot (Fig 16I). Valvula III thickened, sickle-shaped, dorsal surface with long dense pubescence near apex; apical half medially twisted; broadly rounded. Mediotergite IX ogival, shorter than valvula III; lateral margins convex, converging to broadly rounded apex; length 1.3x greatest width; width at base less than twice width of valvula III (Fig 18I).

#### Male genitalia.

Pygophore with elongate setae sparsely distributed over most of surface; anterior margin concave, posterior margin straight. Phallosoma straight (Figs 22U–V). Paramere with anterior margin concave in apical half; mesal angle narrowly rounded, curved anteriorly (Fig 24L).

#### Comments.

The female terminalia of this species are most similar to those of *L. illiesi* and *L. pusillus*. However, in *L. yanomami* the lateral margins of tergum VII are divergent (Fig 8I), whereas in *L. illiesi* they are parallel (Fig 7I). The lateral margins of the subgenital plate are straight in apical 2/3 in *L. yanomami* (Fig 10I), whereas they are concave at midlength in *L. illiesi* (Fig 9I) and in *L. pusillus* they are convex in apical 2/3 (Fig 10A). As for the male genitalia, *L yanomami* differs from the other two species by having the paramere with the anterior margin concave in apical half, with the mesal angle narrowly rounded and curved anteriorly (Fig 24L), while the other two have the anterior margin straight and the mesal angle broadly rounded and not curved (Figs 23I; 24E).

***Limnocoris curvipenis* Canejo, Rodrigues & Moreira, NEW SPECIES**

urn:lsid:zoobank.org:act:8146C988-53B2-49EE-A73F-CDBEB27F509C

(Figs 32–35)

#### Description. Male – Brachypterous.

HOLOTYPE, length 9.40; maximum width 7.20. Paratypes (n = 27), length 8.40–9.40 (mean = 9.11); maximum width 6.60–7.40 (mean = 6.71). General shape oval; widest across embolia. Overall dorsal coloration yellowish-brown, mottled brown on head, pronotum and hemelytra. Hemelytral membrane with small dark spots. Dorsal surface with fine granulations and punctate throughout. Ventral coloration mostly yellowish-brown, except for most of abdominal sterna dark-brown.

##### Head.

Length 1.50, maximum width 3.10. Yellow, with brown markings medially and sometimes parallel to mesal margins of eyes. Synthlipsis 1.40. Eye not raised above level of vertex or pronotum. Anterior margin between eyes convex. Maxillary plate broad basally, anterior edge triangular. Labrum width subequal to length, pentagonal, distal margin tapered. Labium with three visible articles; article IV brown, extending 1 mm beyond labrum not including extruded stylets. Antenna length 0.84, 4-articulated, exceeding lateral margin of eye; scape bulbous, rounded; pedicel subrectangular, wider distally; flagellomeres slender, with long setae; articles I, II, III and IV lengths: 0.10, 0.24, 0.38, 0.12. Postgenal tubercle not reaching level of probasisternal carina.

##### Thorax.

Pronotum ground color yellow, rectangular area behind eyes darker; shallow sulcus marking anterior border of transverse band at posterior third. Anterior margin concave between eyes; lateral margins convergent anteriorly, evenly convex; posterior margin convex; posterolateral corner narrowly rounded, angulated; greatest width 3.4x length at midline; length at midline 1.80; maximum width at posterolateral corner 6.10. Prothorax ventrally brown, with propleuron yellowish. Propleuron with shagreened area extended posteriorly along lateral margin, almost reaching posterolateral corner; posterior margin concave at midlength; posteromesal corner near prosternellum flat. Median carina of probasisternum quadrate in lateral view, extending anteriorly. Scutellum wrinkled, triangular, brown medially, yellow at corners. Hemelytra brown, lighter at embolium; length 6.80 (chord measurement); lateral margin after embolium narrowed, exposing lateral strip of abdominal terga III–V. Embolium greatest width 1.90, lateral margin convex. Claval and intraclaval sutures absent. Hind wings reaching posterior margin of abdominal tergum I. Region between mesobasisternum and mesoepisternum without longitudinal row of elongated golden setae. Mesosternal carina with median ridge straight to slightly convex; fossa oval, shallow, slightly excavated at posterior margin in lateral view. Metasternal carina oval to teardrop shaped, depressed medially; posterior margin not excavated in lateral view (Figs 33D, F).

##### Legs.

All leg segments yellow, except dark-brown apical part of tarsomere III of middle and hind legs. Procoxa with cluster of stout, brown anteromedial spines. Profemur anterior margin with dense pad of brown setae without associated spines, posterior margin with row of short, brown spines along basal half. Protibia and tarsus with occlusal surface flattened; tarsus one-articulated, immovable; pretarsal claw single, minute, triangular. Meso- and metacoxae partially recessed into thorax. Meso- and metafemora with row of short, browninsh spines on anterior margin. Meso- and metatibiae with ventrolateral, ventromedial, dorsolateral, and dorsomedial rows of stout brownish spines; meso- and metatibiae with two transverse rows of spines distally, one each on lateral and mesal margins. Meso- and metatibiae and metatarsus with long, pale swimming setae, setae profuse on metatibia and -tarsus. Meso- and metapretarsi with paired claws slender, gently curved, with minute basal tooth. Leg measurements as follows: fore leg, femur 2.15, tibia 1.28, tarsus 0.48; middle leg, femur 2.40, tibia 1.70, tarsomeres 1–3, 0.16, 0.40, 0.42; hind leg, femur 3.24, tibia 3.36, tarsomeres 1–3, 0.22, 0.90, 0.82.

##### Abdomen.

Dorsally with lateral margins of segments III–V exposed, dark-brown anteriorly, yellow posteriorly; marginal row of short yellow setae, and group of trichobothria near posterior third. Lateral margin with minute serration. Posterolateral corners of II–V narrowly rounded to right angled, not spinose. Median part of sterna brown; lateral margins dark-brown anteriorly, yellow posteriorly. Sterna covered by golden pubescence, without dispersed elongate golden setae; sternum II without irregular patch or row of elongate golden setae. Sternum V asymmetrical, with posterior margin displaced sinistrally (Fig 33B). Mediotergite VI with accessory genitalic process rounded. Posterior margin of mediotergite VII extended posteriorly, convex to truncate; laterotergite VII slender, surpassing level of laterotergite VI (Fig 35A). Lateral lobe of tergum VIII with lateral margin straight to shallowly concave; left medial lobe angled posterolaterally, posteromesal corner rounded; right medial lobe twisted laterally in apical half (Fig 35B). Pygophore with elongate golden setae densely distributed throughout surface (Fig 35D–E). Phallosoma distinctly curved dextrally in distal third, with apex surpassing level of ventral lobes (Figs 35D–G); ventral lobes membranous. Parameres symmetrical, narrow, anterior margin straight; mesal angle narrowly rounded; setae evenly distributed throughout dorsal surface (Fig 35C).

#### Female – Brachypterous.

Paratypes (n = 14) length 8.70–9.70 (mean = 9.28); maximum width 6.80–7.30 (mean = 7.00). Similar to male in general structure and coloration, except for sternum V symmetrical and terminalia structures described below.

#### Female – Macropterous.

Paratypes (n = 1) length 9.40; maximum width 7.10. Similar to brachypterous specimens in general structure and coloration, except as follows: posterolateral corners of pronotum rounded; hemelytra with claval and intraclaval sutures distinct; hind wings fully developed, extending beyond tip of abdomen.

#### Female terminalia.

Tergum VII width about 2.4x length at midline; dense pubescence extending up to half the length of lateral lobe; lateral margin sinuous, convex in basal half; posterior margin convex; lateral lobe length twice width, divergent at base, convergent at apex; posterolateral margin bearing longer setae (Fig 34A). Subgenital plate distinctly projected posterolaterally in basal half, forming a small lobe; posterolateral margin with longer setae; apex rounded. Laterosternite VII straight at mesal margin, bearing single tuft of long setae posteriorly; lateral margin shallowly concave, almost straight; apex narrowly rounded (Fig 34B). Mediotergite VIII with lateral margin concave; posterior margin rounded or truncate. Laterotergite VIII length twice greatest width; distal half of lateral margin weakly serrated, bearing minute brush-like setae (Fig 34C). Valvifer I with mesal margin convex; anterior region with few setae; posterior region bearing dense pilosity. Valvula I bearing robust, posteriorly directed setae, setae becoming longer towards apex; apical robust setae longer than lateral margin (Fig 34D). Valvulae II slightly wider than long; lateral margin slightly convex to straight, with long setae; apex rounded to truncate, without apical dark spot (Fig 34E). Valvula III thickened, cone-shaped, with dense, long pubescence; apex rounded in lateral view. Mediotergite IX triangular, shorter than valvula III; lateral margins convex, converging to narrowly rounded apex; length 1.5x greatest width; width at base less than twice width of valvula III (Fig 34F).

#### Diagnosis.

Body length 8.40–9.70; body width 6.60–7.40. This species can be easily distinguished from congeners by the curved male phallosoma (Fig 35E) and the female subgenital plate distinctly projected posterolaterally in basal half, forming a small lobe (Fig 34B). Both features are unique within *Limnocoris*.

#### Comparative notes.

This species is morphologically similar to *L. decarloi* in general body structure, size and coloration. They can be easily distinguished by the shape of the phallosoma in males, and the shape of the lateral lobe of tergite VII and the subgenital plate in females.

#### Distribution and habitat.

This species is known from the states of Paraná and São Paulo, eastern Brazil, where it was collected in streams near waterfalls, associated with sandy substrate.

#### Etymology.

The specific epithet is derived from the combined Latin words *curvi* (=curved) and *penis* (=penis, aedeagus), referring to the distinct curved shape of the male phallosoma.

#### Type material examined.

HOLOTYPE, ♂: **BRAZIL, Paraná**: Morretes, PR12, Cachoeira do Jajá, 25°34’34.8“S, 48°49’43.6”W, 244 m, 30.x.2023, R.P.R. Canejo, J.M.S. Rodrigues, M.S.L. Alexandre & L.D. Pereira col. (CEIOC). PARATYPES: same data as holotype (17♂, 7♀ brachypterous, CEIOC); same data as holotype (1♂, 1♀ brachypterous, MZUSP); Paranaguá, PR15, Parque Nacional Saint Hilaire/Lange, Cachoeira Quintilha, 25°38’28.9”S, 48°37’12.5”W, 125 m, 30.x.2023, R.P.R. Canejo, J.M.S. Rodrigues, M.S.L. Alexandre & L.D. Pereira col. (1♂ brachypterous, CEIOC). **São Paulo**: Capão Bonito, SP7, Parque Estadual Carlos Botelho, Cachoeira do Muriqui, 24°06’44.8”S, 47°59’44.0”W, 802 m, 17.xi.2023, L.L. Dumas, J.M.S. Rodrigues & R.P.R. Canejo col. (8♂, 5♀ brachypterous, 1♀ macropterous, CEIOC); Sete Barras, SP42, Parque Estadual Carlos Botelho, Cachoeira do Quilombo, 24°12’34.6”S, 48°03’14.8”W, 147 m, 07.xii.2023, L.L. Dumas, J.M.S. Rodrigues, L. Nery & L. Hoehne col. (1♂ brachypterous, CEIOC).

***Limnocoris sitesi* Canejo, Rodrigues & Moreira, NEW SPECIES**

urn:lsid:zoobank.org:act:1181CF6E-FCB7-482E-A376-34F0773B5B5F

(Figs 32, 36–38)

#### Description. Male – Brachypterous.

HOLOTYPE, length 8.30; maximum width 6.18. Paratypes (n = 5), length 7.70–8.40 (mean = 7.98); maximum width 5.60–6.40 (mean = 5.93). General shape oval; widest across embolia. Overall dorsal coloration brown, mottled yellow on head and pronotum; hemelytra solid brown, except for yellowish-brown markings on embolium and membrane. Dorsal surface with fine granulation, punctate throughout. Ventral coloration mostly dark-brown, except yellowish-brown on propleuron and lateral part of abdominal sterna.

**Fig 37:**
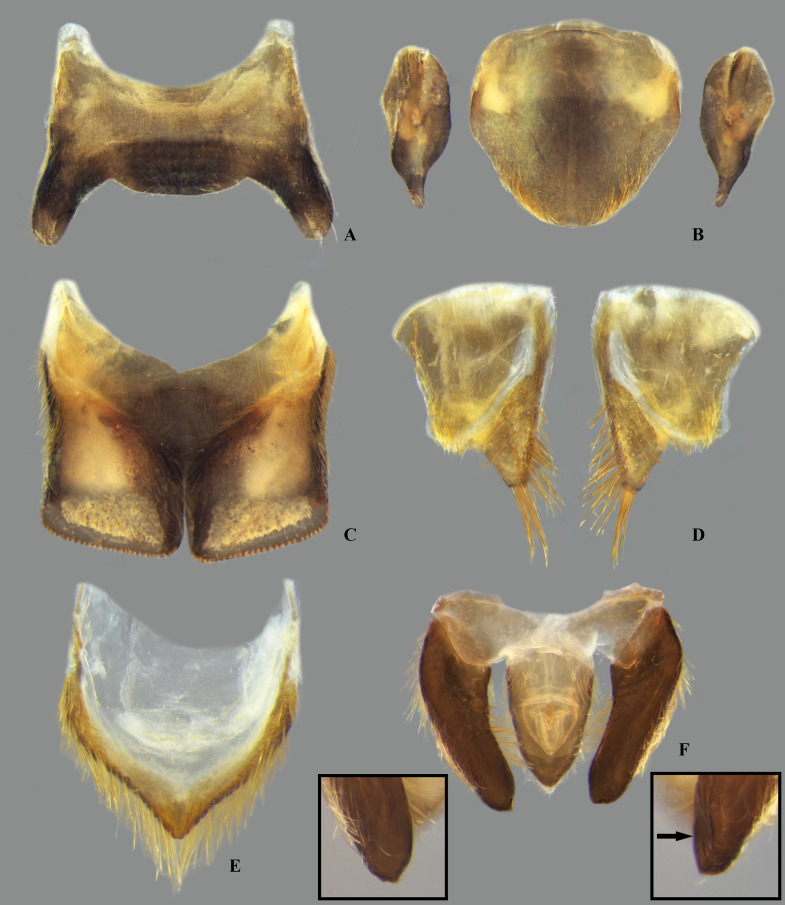
*Limnocoris sitesi* Canejo, Rodrigues & Moreira n. sp. Female terminalia. **(A)** Abdominal tergum VII, (B) abominal mediosternite VII (subgenital plate) + laterosternites VII, (C) abdominal tergum VIII, **(D)** Valvifer I + Valvulae I, **(E)** Valvulae II, **(F)** Valvifer II + Valvulae III + abdominal mediotergite IX, insets showing apex of valvulae III in lateral view, arrow indicates concavity. Images not to scale.

**Fig 38:**
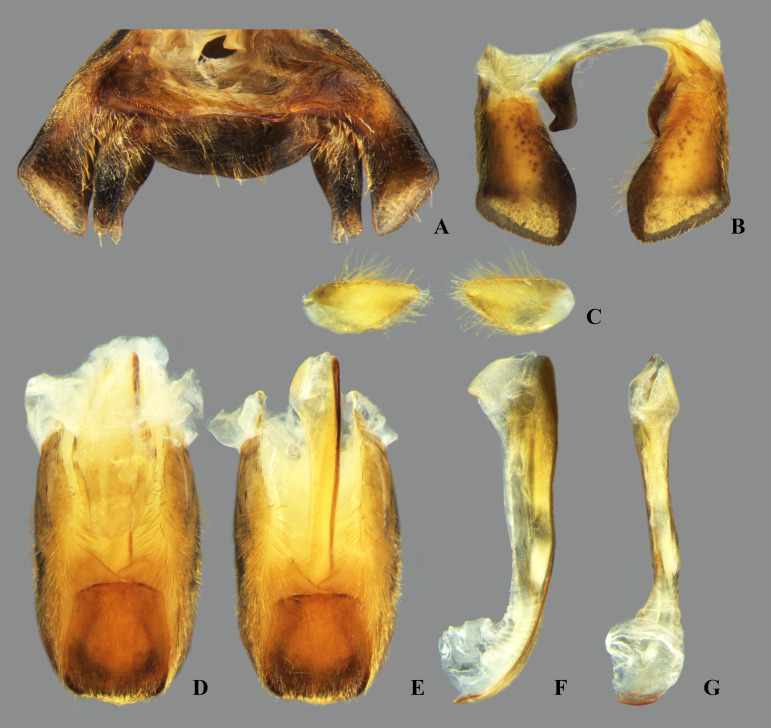
*Limnocoris sitesi* Canejo, Rodrigues & Moreira n. sp. Male terminalia. **(A)** Abdominal terga VI + VII, **(B)** abdominal tergum VIII, **(C)** parameres, **(D)** fenital capsule, **(E)** fenital capsule (proctiger removed) showing phallosoma in dorsal view, **(F)** phallosoma in lateral view and **(G)** phallosoma in ventral view. Images not to scale.

##### Head.

Length 1.45, maximum width 2.80. Yellow with brown markings. Synthlipsis 1.45. Eye not raised above level of vertex or pronotum. Anterior margin between eyes convex. Maxillary plate broad basally; anterior edge triangular. Labrum pentagonal; width 1.75x length; distal margin tapered to broadly rounded. Labium with three visible articles, extending 2.0 beyond labrum not including extruded stylets; article III length equal to article IV; article IV brown. Antenna length 0.90.; 4-articulated, exceeding lateral margin of eye; scape bulbous, rounded; pedicel subrectangular, wider distally; flagellomeres slender, with long setae; articles I, II, III and IV lengths: 0.10, 0.22, 0.34, 022. Postgenal tubercle not reaching level of probasisternal carina.

##### Thorax.

Pronotum yellow; dark-brown markings concentrated at rectangular area behind eyes; shallow sulcus marking anterior border of transverse band in posterior third. Anterior margin concave between eyes; lateral margins convergent anteriorly, evenly convex; posterior margin sinuous, shallowly concave medially; posterolateral corner rounded, not projected posteriorly; greatest width 3.62x length at midline; length at midline 1.55; maximum width at posterolateral corner 5.62. Prothorax ventrally yellowish-brown. Propleuron with shagreened area extended posteriorly 3/4 along lateral margin, narrow at apex (Fig 36C); posterior margin concave at mid-length; posteromesal corner near prosternellum flat. Median carina of probasisternum bifurcated, extending anteriorly in lateral view. Scutellum wrinkled, triangular, brown with a conspicuous yellow spot posteriorly. Hemelytra brown, lighter at embolium; membrane with small, lighter markings; length 5.87 (chord measurement); lateral margin after embolium narrowed, exposing a strip of abdominal terga III–V. Embolium greatest width 0.71, lateral margin convex. Claval and intraclaval sutures absent. Hind wings reaching posterior margin of abdominal tergum I. Region between mesobasisternum and mesoepisternum without longitudinal row of elongated golden setae. Mesosternal carina with median ridge straight (Fig 36E) or convex (Fig 36F); fossa oval, shallow. Metasternal carina oval to teardrop-shaped, depressed medially; posterior margin not excavated in lateral view (Figs 36E–F).

##### Legs.

All leg segments yellowish brown, except for dark-brown apical part of tarsomere III of middle and hind legs. Procoxa with cluster of stout, brown anteromedial spines. Profemur anterior margin with dense pad of brown setae without associated spines, posterior margin with row of short, brown spines along basal half. Protibia and tarsus with occlusal inner surface flattened; tarsus one-articulated, immovable; pretarsal claw single, minute, triangular. Meso- and metacoxae partially recessed into thorax. Meso- and metafemora with row of short, browninsh spines on anterior margin. Meso- and metatibiae with ventrolateral, ventromedial, dorsolateral, and dorsomedial rows of stout brownish spines; meso- and metatibiae with two transverse rows of spines distally, one each on lateral and mesal margins. Meso- and metatibiae and metatarsus with long, pale swimming setae, setae profuse on metatibia and -tarsus. Meso- and metapretarsi with paired claws slender, gently curved, with minute basal tooth. Leg measurements as follows: foreleg, femur 2.21, tibia 1.37, tarsus 0.5; middle leg, femur 2.25, tibia 1.65, tarsomeres 1–3: 0.12, 0.3, 0.4; hind leg, femur 2.84, tibia 2.90, tarsomeres 1–3: 0.18, 1.03, 0.78.

##### Abdomen.

Dorsally with lateral margins of segments III–V exposed; terga III–V dark-brown anteriorly, yellowish posteriorly; marginal row of short yellow setae and group of trichobothria near posterior third. Lateral margin weakly serrated. Posterolateral corners of II–V narrowly rounded to right-angled, not spinose. Median part of sterna brown; posterolateral margins yellowish-brown. Sterna covered by golden pubescence, without dispersed elongate golden setae; sternum II without irregular patch or row of elongate golden setae. Sternum V asymmetrical, with posterior margin slightly displaced sinistrally. Mediotergite VI lacking accessory genitalic process. Posterior margin of mediotergite VII convex; laterotergite VII slender, reaching level of laterotergite VI (Fig 38A). Lateral lobe of tergum VIII with lateral margin straight at anterior half, angled at midlength. Left medial lobe angled posterolaterally; posteromesal corner rounded; right medial lobe twisted laterally in apical third (Fig 38B). Pygophore with elongate setae distributed over most of surface, including a dense tuft at median region of posterior margin; anterior margin convex; posterior margin nearly straight (Fig 38D). Phallosoma straight at right margin, wider distally at left margin; ventral lobes membranous (Figs 38D–G). Paramere with anterior margin straight; mesal angle narrowly rounded; setae evenly distributed throughout dorsal surface (Fig 38C).

#### Female – Brachypterous.

Paratypes (n = 2) length 8.40–8.60 (mean = 8.50); maximum width 6.30–6.40 mm (mean = 6.35). Similar to male in general structure and coloration, except for sternum V symmetrical and terminalia described below.

#### Female terminalia.

Tergum VII width about 2.4x length at midline; lateral margin sinuous, slightly convex at mid-length; posterior margin convex; lateral lobes diverging, longer than wide, rounded at apex (Fig 37A). Subgenital plate with fringe of longer setae on posterolateral margin; lateral margins slightly divergent in basal half, converging to broadly rounded posterior margin. Laterosternite VII convex at mesal margin, lateral margin sinuous, concave at midlength; apex bearing single tuft of long setae, narrowly rounded (Fig 37B). Mediotergite VIII with lateral margin shallowly concave; posterior margin rounded. Laterotergite VIII with length less than twice width; basal half of lateral margin shallowly concave; distal half weakly serrated, bearing minute brush-like setae (Fig 37C). Valvifer I with mesal margin convex; anterior region with few setae, posterior region bearing dense pilosity. Valvula I bearing robust, posteriorly directed setae, setae becoming longer towards apex; apical robust setae longer than lateral margin (Fig 37D). Valvulae II slightly wider than long; lateral margin slightly sinuous, with long setae; apex narrowly rounded to acuminate, without apical dark spot (Fig 37E). Valvula III thickened, wider at base, with long dense pubescence; apex rounded; mesal surfaces of apex asymmetrical, with one valvula convex and the other concave (sides varied in females examined). Mediotergite IX ogival, shorter than valvula III; lateral margins nearly straight, converging to narrowly rounded apex; length 1.6x greatest width; width at base less than twice width of valvula III (Fig 37F).

#### Diagnosis.

Body length 7.70–8.40, body width 5.60–6.40. The shagreened area of the propleuron extends posteriorly for approximately the anterior 2/3 of the lateral margin and is distinctly narrow at the apex (Fig 36C). The posterolateral margin of the female subgenital plate bears a fringe of long golden setae (Fig 37B). Valvulae III are asymmetrical, with the apex concave on one side and convex on the other (Fig 37F).

#### Comparative notes.

This species has the shape of the shagreened area of the propleuron similar to that of *L. pauper*, extending posteriorly for approximately the anterior 2/3 of the lateral margin and distinctly narrow at the apex. Also, the shape of female abdominal tergum VII of this new species is similar to that of *L. decarloi*. However, the overall body shape of *L. sitesi* **n. sp.** is unique, including the asymmetry in valvulae III, with the apex concave medially on one side and convex on the other (Fig 37F).

#### Distribution and habitat.

This species was collected in sandy substrates of shallow streams in the states of Paraná and São Paulo, Brazil.

#### Etymology.

The specific epithet is in honor of our colleague Robert W. Sites (University of Missouri, United States), in recognition of his important contributions to the study of Naucoridae worldwide and his enthusiasm for sharing information about these insects.

#### Type material examined.

All specimens brachypterous. HOLOTYPE, ♂: **BRAZIL, Paraná**: Morretes, PR10, Recanto Rio Cascata, 25°20’02.7“S, 48°53’54.4”W, 805 m, 27.x.2023, R.P.R. Canejo, J.M.S. Rodrigues, M.S.L. Alexandre & L.D. Pereira col. (CEOIC). PARATYPES: same data as holotype (3♂ CEIOC); same data as holotype (1♂ MZUSP); Campina Grande do Sul, Parque Estadual Pico do Paraná, Cachoeira do Arco–Íris, antes da queda, 25°13’16.8”S, 48°51’26.0”W, 960 m, 31.x.2023, R.P.R. Canejo, J.M.S. Rodrigues, M.S.L. Alexandre & L.D.Pereira col. (3♂, 1♀ CEIOC); **São Paulo**: Eldorado, CDD06, Ribeirão Martins, 24°39’40.8”S, 48°16’32.0”W, 73 m, 03.vii.2024, E.A. Joaquim, L.L. Dumas, K.O. Souza & R.P.R. Canejo col. (1♂, 1♀ CEIOC).

## Discussion

The description of the structures of the female terminalia and the male genital capsule revealed many characters that proved to be extremely useful for the taxonomy of *Limnocoris*. With the synonymies proposed here, the resurrection of *L. admontandoni* and *L. sattleri* and the description of two new species, the number of valid species in the genus is increased to 76.

The female terminalia, mostly unexplored for more than a century, showed a pool of diagnostic characters for almost all species examined in the study. In particular, the female abdominal tergum VII is an important structure for species identification, showing high interspecific and low intraspecific variation (Figs 7–8). Although valvula III presents a predominantly uniform morphology among closely related species, modifications were observed in some of them, including a distinct indentation on the posterior region of the ventral surface (Figs 17J, 18C–D, 26F). The valvulae I–II, which form the ovipositor, showed little morphological variation compared to the other structures analyzed. However, considering that some species of *Limnocoris* demonstrate possible habitat specificity, as they are associated with certain substrate types [2,4], these valvulae may provide clues to the oviposition behavior of the genus, an aspect that has not yet been studied.

Male terminalia have been widely used in taxonomic studies of Naucoridae. Even in Neotropical genera with high interspecific variability in these structures, such as *Ambrysus* Stål, 1860 [17–21] and *Maculambrysus* Reynoso & Sites, 2021 [22], the female terminalia are still useful for specific differentiation. Although few structures have been studied in these genera, such as the subgenital plate, their interspecific variability has diagnostic value. The lack of detailed studies on female terminalia may be related to the sufficiency of already known structures to differentiate species and species groups in most taxa. In Neotropical genera where male terminalia are rarely used in taxonomic studies due to their uniformity among species, such as *Cryphocricos* Signoret, 1850 and *Limnocoris*, researchers would be expected to focus on female terminalia in search of diagnostic characters. However, prior to this study, these structures remained unexplored.

In *Limnocoris*, the ventral lobes of the phallosoma are membranous (Fig 3C), which probably explains the low morphological variability observed in this structure compared to other naucorid genera, in which the ventral lobes are sclerotized [15–20]. Nevertheless, we recorded for the first time a male phallosoma with a distinct morphology, characterized by a curvature in its apical portion (Figs 35D–G). Considering that only 28 of the 76 currently valid species of the genus were included in this study, it is possible that other unanalyzed species also exhibit modifications in the male genitalia. Further studies are needed to test this hypothesis.

The identification of diagnostic characters in these previously neglected structures in *Limnocoris* provides a basis for future taxonomic studies, including redescriptions, descriptions of new species, and their inclusion in identification keys. These characters can also be used in phylogenetic studies on this and other naucorid genera.
